# A global assemblage of regional prescribed burn records — GlobalRx

**DOI:** 10.1038/s41597-025-04941-w

**Published:** 2025-07-01

**Authors:** Alice Hsu, Matthew W. Jones, Jane R. Thurgood, Adam J. P. Smith, Rachel Carmenta, John T. Abatzoglou, Liana O. Anderson, Hamish Clarke, Stefan H. Doerr, Paulo M. Fernandes, Crystal A. Kolden, Cristina Santín, Tercia Strydom, Corinne Le Quéré, Davide Ascoli, Marc Castellnou, Johann G. Goldammer, Nuno Ricardo Gracinhas Nunes Guiomar, Elena A. Kukavskaya, Eric Rigolot, Veerachai Tanpipat, Morgan Varner, Youhei Yamashita, Johan Baard, Ricardo Barreto, Javier Becerra, Egbert Brunn, Niclas Bergius, Julia Carlsson, Chad Cheney, Dave Druce, Andy Elliot, Jay Evans, Rodrigo De Moraes Falleiro, Nuria Prat-Guitart, J. Kevin Hiers, Johannes W. Kaiser, Lisa Macher, Dave Morris, Jane Park, César Robles, Rosa María Román-Cuesta, Gernot Rücker, Francisco Senra, Lara Steil, Jose Alejandro Lopez Valverde, Emma Zerr

**Affiliations:** 1 School of Environmental Sciences, University of East Anglia, Norwich, NR4 7TJ UK; 2 Tyndall Centre for Climate Change Research, University of East Anglia, Norwich, NR4 7TJ UK; 3 School of Global Development, University of East Anglia, Norwich, NR4 7TJ UK; 4 School of Engineering, University of California, Merced, CA 95343 USA; 5 National Centre for Monitoring and Early Warning of Natural Disasters, Cemaden, Estrada Doutor Altino Bondensan, 500, Distrito de Eugênio de Melo, São José dos Campos, São Paulo, Brazil; 6 FLARE Wildfire Research, School of Agriculture, Food and Ecosystem Sciences, University of Melbourne, Grattan St, Parkville, 3010 Australia; 7 Centre for Wildfire Research, Swansea University, Swansea, SA2 8PP UK; 8 Centre for the Research and Technology of Agro-Environmental and Biological Sciences, University of Trás-os-Montes and Alto Douro, Quinta de Prados, 5000-801 Vila Real, Portugal; 9 Research Institute of Biodiversity, CSIC-University of Oviedo, Principality of Asturias, Mieres, 33600 Spain; 10 Scientific Services, South African National Parks, Private Bag X402, Skukuza, 1350 South Africa; 11 Department of Agriculture, Forest and Food Sciences, University of Torino, Largo Paolo Braccini 2, 10095 Grugliasco, Italy; 12 GRAF, Catalan Fire Service. 08019 Cerdanyola del Valles, Catalonia, Spain. University of Lleida, Lleida, Spain; 13 Global Fire Monitoring Center, Max Planck Institute for Chemistry and Freiburg University, Georges-Koehler-Allee 75, Freiburg, Germany; 14 MED, Mediterranean Institute for Agriculture, Environment and Development; CHANGE, Global Change and Sustainability, University of Évora-PM, Pólo da Mitra, Apartado 94, 7006-554 Évora, Portugal; 15 EaRSLab, Earth Remote Sensing Laboratory, University of Évora-CLV, Rua Romão Ramalho, 59, 7000-671 Évora, Portugal; 16 IIFA, Institute for Advanced Studies and Research, University of Évora-PV, Largo Marquês de Marialva, Apartado 94, 7002-554 Évora, Portugal; 17 V.N. Sukachev Institute of Forest of the Siberian Branch of the Russian Academy of Sciences - separate subdivision of the Federal Research Center “Krasnoyarsk Science Center SB RAS”, 50/28 Akademgorodok, Krasnoyarsk, 660036 Russia; 18 National Research Institute for Agriculture, Food, and Environment - Research unit 0629 - 228, route de l’Aérodrome, CS 40509, 84914 Avignon Cedex 9, France; 19 Upper ASEAN Wildland Fire Special Research Unit, Kasetsart University, 50 Ngamwongwan Rd, Lat Yao, Chatuchak, Bangkok, 10900 Thailand; 20 Tall Timbers Research Station, 13093 Henry Beadel Drive, Tallahassee, FL 32312 USA; 21 Faculty of Environmental Earth Science, Hokkaido University, N10, W5, Kita-ku, Sapporo, Hokkaido, 060-0810 Japan; 22 South African National Parks, Garden Route Scientific Services, George, South Africa; 23 Brazilian Institute of Environment and Renewable Natural Resources (IBAMA), SCEN – Trecho 2, Ed. sede do IBAMA, 70.818-900 Brasília/DF, Brazil; 24 Centro de Observación y Teledetección Espacial S.A.U. - COTESA, C. de la Antracita, 7, Módulo 17, Arganzuela, 28045 Madrid, Spain; 25 Deutsche Bundesstiftung Umwelt Naturerbe GmbH, An der Bornau 2, 49090 Osnabrück, Germany; 26 Länsstyrelsen i Västmanlands län, 721 86 Västerås, Västmanland, Sweden; 27 Table Mountain National Park, South African National Parks, Cape Town, South Africa; 28 Ecological Advice, Ezemvelo KZN Wildlife, Hluhluwe-iMfolozi Park, Hluhluwe Area, South Africa; 29 School of Life Sciences, University of KwaZulu-Natal, Pietermaritzburg, South Africa; 30 Life and Environmental Sciences, School of Geography, Earth, and Environmental Sciences, University of Birmingham, Birmingham, B15 2TT UK; 31 Forestry Commission, West Midlands, England, UK; 32 Pau Costa Foundation. Av. Mossèn Cinto Verdaguer, 42 Esc. A Bxs 2a, 08552 Taradell Barcelona, Spain; 33 Texas A&M University, Natural Resources Institute, Washington, DC 20006 USA; 34 Klima- og miljøinstituttet NILU, PO Box 100, 2027 Kjeller, Norway; 35 The Queen’s House, Forestry Commission, Lyndhurst, SO43 7NH UK; 36 Banff National Park, Parks Canada Agency, Banff, Alberta T1L1K2 Canada; 37 National Forestry Commission (CONAFOR), Priv. de Almendros 106, Reforma, 68050 Oaxaca de Juárez, Oax. Mexico; 38 Center for International Forestry Research (CIFOR), ICRAF Headquarters. United Nations Avenue. Gigiri, 0100 Nairobi, Kenya; 39 Technische Universitat Munchen (TUM). School of Life Sciences Technical University of Munich Hans-Carl-von-Carlowitz-Platz 2, 85354 Freising, Germany; 40 ZEBRIS Geo-IT GmbH, Lipowskystr. 26, 81373 Munich, Germany; 41 Government of Andalucia, Palacio San Telmo, ES-41004 Seville, Spain; 42 Prevfogo—National Center for Wildfire Prevention and Suppression, Ibama—Brazilian Institute for the Environment and Renewable Natural Resources—SCEN Trecho 2, Edifício Sede, bloco E, Cep, 70818-900 Brasília, DF Brazil; 43 Government of the Principality of Asturias, Plaza de España, 6, ES-3007 Oviedo, Spain; 44 Parks Canada, 7511 Columbia Ave, Radium Hot Springs, BC V0A 1M0 Canada

**Keywords:** Climate-change mitigation, Climate change, Fire ecology

## Abstract

Prescribed burning (RxB) is a land management tool used widely for reducing wildfire hazard, restoring biodiversity, and managing natural resources. However, RxB can only be carried out safely and effectively under certain seasonal or weather conditions. Under climate change, shifts in the frequency and timing of these weather conditions are expected but analyses of climate change impacts have been restricted to select few regions partly due to a paucity of RxB records at global scale. Here, we introduce GlobalRx, a dataset including 204,517 RxB records from 1979–2023, covering 16 countries and 209 terrestrial ecoregions. For each record, we add a comprehensive suite of meteorological variables that are regularly used in RxB prescriptions by fire management agencies, such as temperature, humidity, and wind speed. We also characterise the environmental setting of each RxB, such as land cover and protected area status. GlobalRx enables the bioclimatic range of conditions suitable for RxB to be defined regionally, thus unlocking new potential to study shifting opportunities for RxB planning and implementation under future climate.

## Background & Summary

Prescribed burning (RxB) is a prominent land management tool used globally to accomplish a range of ecological, economic, and societal objectives. In many fire-prone regions such as savannas, shrublands, and dry temperate forests, RxB is used to reduce excessive fuel loads accumulated under fire exclusion and the suppression of Indigenous and traditional fire use, which resulted in increased wildfire extent and severity, loss of native biodiversity, and decreased landscape resilience^1–3^. When applied in strategic locations and at sufficient frequencies and extents, RxB can help reduce the incidence, extent, and intensity of wildfires, thereby aiding fire suppression efforts and reducing damage and losses due to wildfire^4–6^. In fire-adapted ecosystems, RxB can help restore and maintain native flora and fauna habitat, increasing native biodiversity and also protecting against wildfires beyond the adaptation capacity of these species^7–9^. In some ecosystems, RxB may also mitigate carbon emissions from wildfires, reducing the extent and severity of burned area compared to wildfire^10,11^.

Globally, fire is used in both fire-sensitive and fire-adapted ecosystems for agriculture, pastoralism, and managing subsistence resources, such as local food staples and other non-timber forest products^12–14^. RxB can play a key role in maintaining livelihoods that rely on these uses while minimising negative ecological impacts of fire when implemented within an integrated fire management (IFM) framework^15^. IFM calls for ecologically and socially appropriate approaches to managing wildfire risk and fire use^16^. Although not often a formal objective of RxB, the practice has also in some places reinvigorated and recognized local knowledge and contributed to correcting some of the injustices accrued to Indigenous communities in colonial and conservation periods of fire exclusion^17–19^.

There is a growing recognition that RxB, informed by Indigenous and traditional knowledge, can play a key role in restoring native vegetation, maintaining landscape resilience, and sustaining local economies^19^. However, the application of RxB often involves the balancing of multiple and sometimes conflicting land management objectives and potential adverse effects, many of which are not well-understood^20,21^. For example, land managers applying RxB to reduce fuel loads must often balance the interval, season, and pattern of its application with the phenology and life cycles of important plant and animal species to minimise mortality and allow sufficient time and space to recover^22,23^. Similarly, RxB programs must balance the timing and extent local fire use needs (or the lack thereof) with conservation policies and ecosystem-specific fire ecology^24–26^. Crucially, one of the primary constraints on RxB is the occurrence of appropriate weather conditions that can facilitate the fire behaviour necessary to accomplish the desired objectives. These constraints are also complicated by a changing climate, which may diminish the protective effect of RxB against wildfire under increasingly extreme fire weather, and also further reduce the limited opportunities to conduct RxBs^27^.

The continuing and expanding use of RxB highlight the need for continued interdisciplinary research on the objectives, implementation strategies, and the social and ecological effects of RxB, especially under a changing climate^28–30^. Central to addressing these challenges is an improved understanding of the patterns and trends in RxB practices, of which continued, long-term quantitative and qualitative data is a key component.

### Meteorological constraints on prescribed burning

Weather is one of the primary constraints on RxB^31,32^. RxB weather must facilitate fire behaviour such that burns are not so intense that they result in excessive plant mortality^33^, undesirable changes to soil properties^34^, vegetation type conversion^35^, and risk to human lives. On the other hand, RxBs conducted at insufficient intensity may consume too little fuel to effectively mitigate wildfire or provide ecological benefit^36,37^. In many regions, consideration for air quality must also be taken into account, further limiting the days RxBs may be carried out. In many countries, prior to burning, a written and approved plan must define the specific weather and fuel moisture conditions required to facilitate the fire behaviour necessary for achieving the desired management objectives^32,38^. These plans are sometimes legally binding, and burns may not be carried out if weather conditions are not met^39,40^. The period of time during which suitable meteorological conditions and other factors such as air quality and resource allocation are achievable are referred to as the prescription burn window (RxBW)^36,39^.

Agencies and land managers seeking to carry out RxBs determine the RxBW that are suitable based on technical guidelines and regulations, ecosystem-specific knowledge of vegetation and fire behaviour and ecology, meteorological information or forecasts, and practical experience^41^. The weather-related metrics used to regulate RxB or guide decision-making can include temperature, humidity, wind, precipitation, fuel moisture, and fire danger indices that integrate multiple meteorological variables into an overall rating of potential for dangerous fire behaviour^42^. Operational limits can vary across land covers, ecoregions and fuel types. RxBs are generally concentrated in seasons when conditions are more likely to be favourable to fire control, such as in autumn or spring in the extratropics and the early dry season in the tropics.

### Climate change impacts on prescribed burn windows

Climate change is raising temperatures and increasing the frequency of dry extremes globally, leading to increased fire danger^43,44^. Under future climate change, the RxBW may lengthen, shorten, or shift seasonally, meaning a potential for change in the opportunities to conduct RxBs and a need to consider future resource needs^36,45,46^. Previous work has shown that the meteorological window of opportunity to conduct RxBs is shortening in the western US alongside a lengthening of the wildfire season due to climate change^47,48^. Climate model projections also indicate that the historical meteorological window of RxB opportunity is shortening during summer months in the southeast US as extreme fire weather becomes more frequent^46^. Regional changes in the duration of the weather window for RxBs have also been projected in Australia^27,36,49^. However the direction of change varies regionally and trends can differ depending on how RxBWs are defined^45,49,50^.

Until now, there has been no global database of RxBs. Consequently, analyses of shifts in weather windows have been concentrated in a small number of regions with consolidated datasets that are easily accessed (chiefly in North America and Australia). Additionally, comparison of RxB uses and RxBWs within and across countries and ecosystems has been limited. Given the future projections of increased fire-prone weather under climate change, it is increasingly important that agencies and practitioners of RxB are equipped with quantitative and qualitative information about how current practices, resource allocation, and regulations may need to adapt in the future to ensure that RxBs can remain safe and effective.

### Global data to inform analyses of prescribed burns

**Fig. 1:**
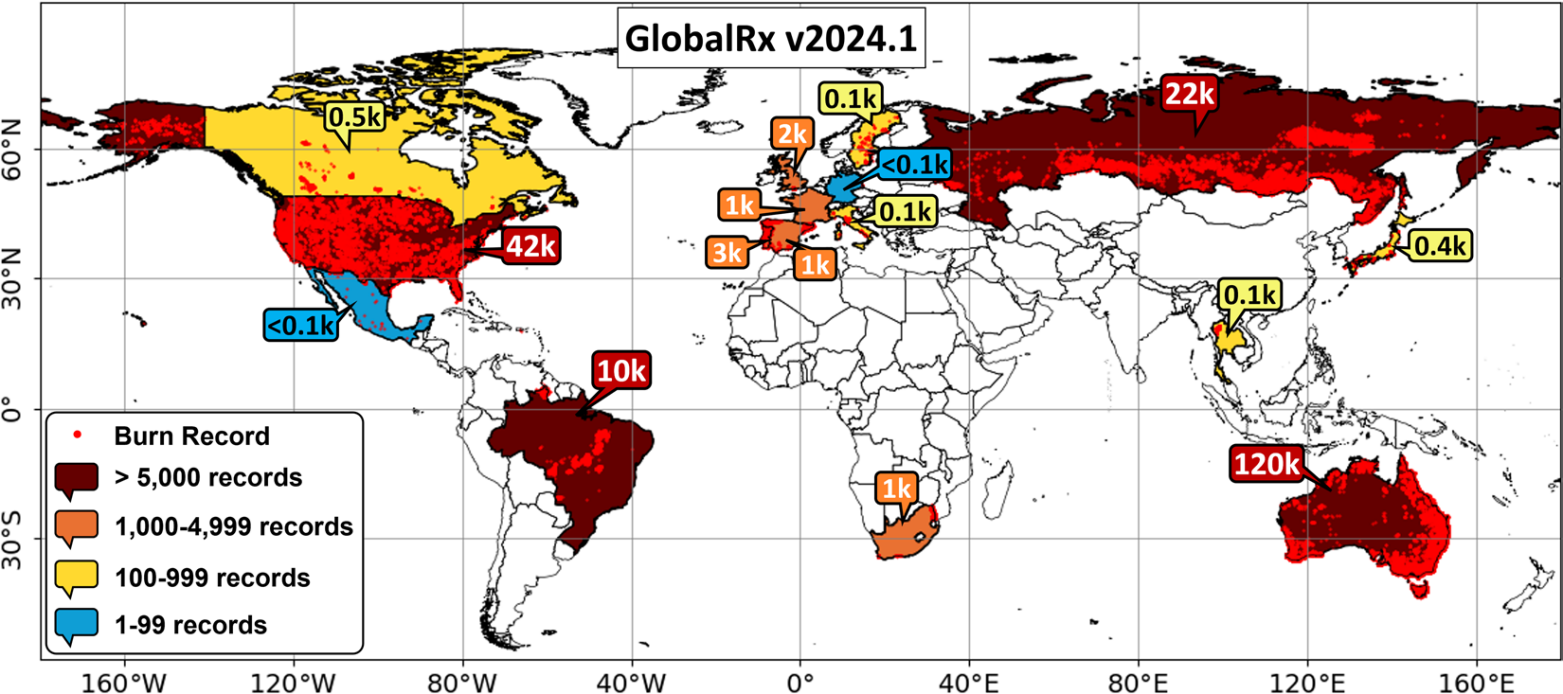
Prescribed burns from the datasets collated in the GlobalRx dataset (Supplementary Table 1), which includes records from 1979–2023 across 16 countries and 209 ecoregions. Countries are coloured in according to the number of records they have within GlobalRx.

**Fig. 2:**
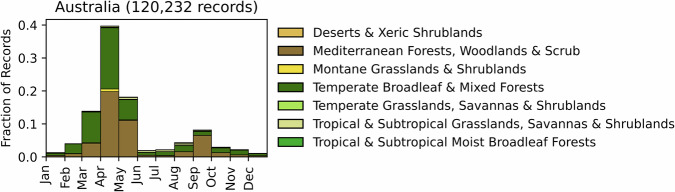
Distribution of RxB records by month of the year for each biome in the country of Australia. The total number of records falling within a biome are indicated in the title. Note that not all records fall within a biome boundary, so that the number of total records for the country may not match the number indicated in the title.

**Fig. 3:**
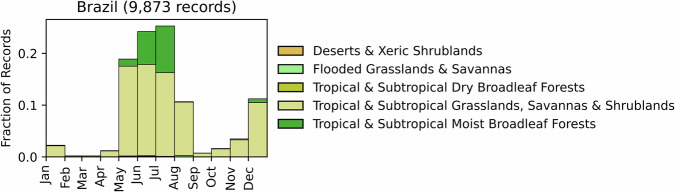
Distribution of RxB records by month of the year for each biome in the country of Brazil. The total number of records falling within a biome are indicated in the title.

**Fig. 4:**
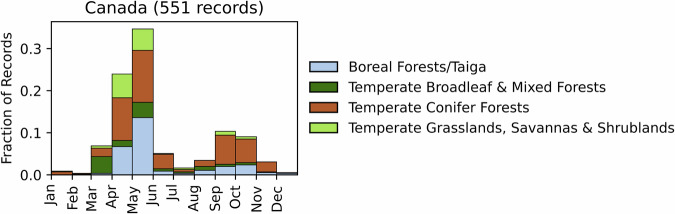
Distribution of RxB records by month of the year for each biome in the country of Canada. The total number of records falling within a biome are indicated in the title. Note that not all records fall within a biome boundary, so that the number of total records for the country may not match the number indicated in the title.

**Fig. 5:**
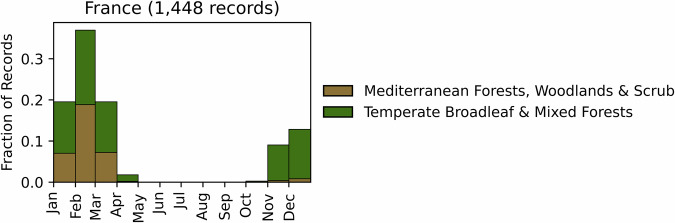
Distribution of RxB records by month of the year for each biome in the eastern Pyrenees of France. The total number of records falling within a biome are indicated in the title.

**Fig. 6:**
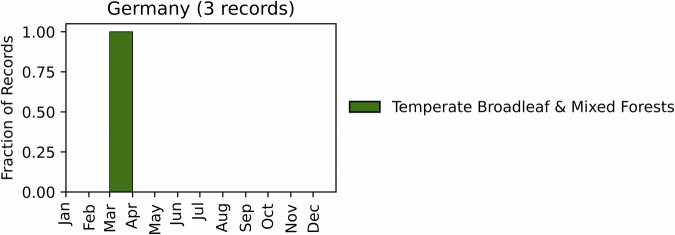
Distribution of RxB records by month of the year for each biome in the country of Germany. The total number of records falling within a biome are indicated in the title.

**Fig. 7:**
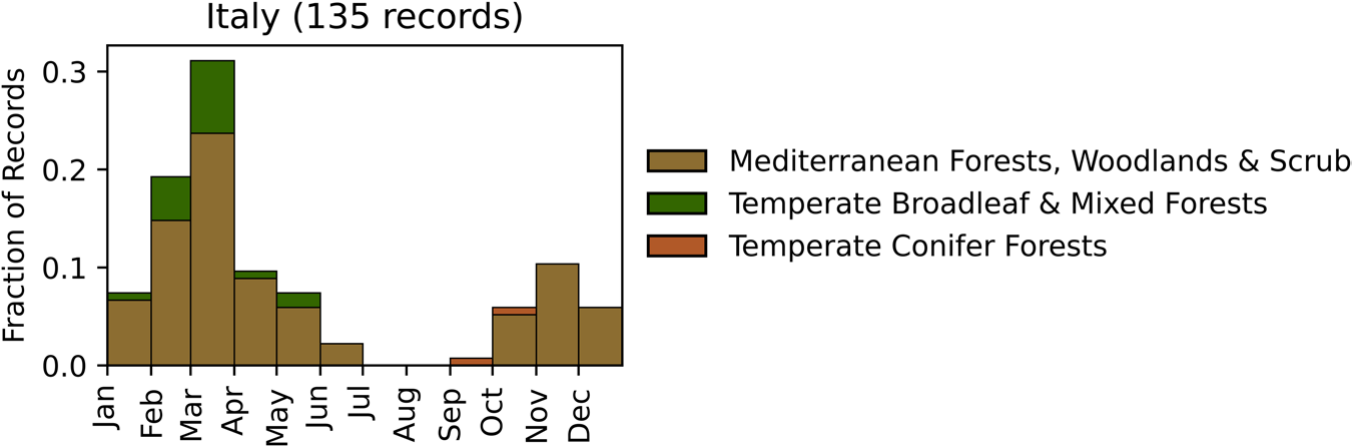
Distribution of RxB records by month of the year for each biome in the country of Italy. The total number of records falling within a biome are indicated in the title.

**Fig. 8:**
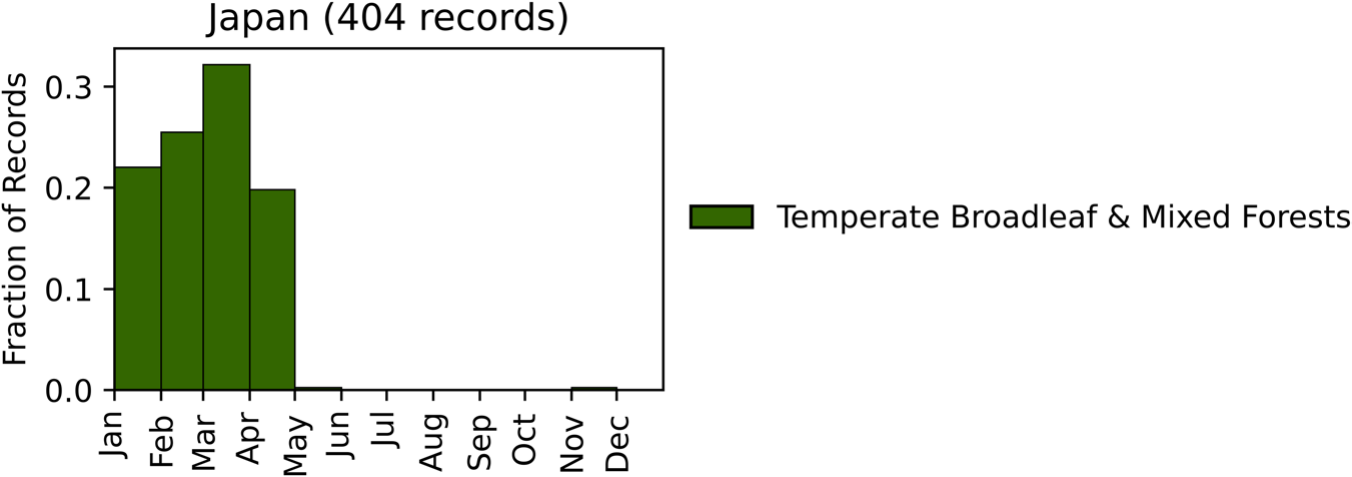
Distribution of RxB records by month of the year for each biome in the country of Japan. The total number of records falling within a biome are indicated in the title. Note that not all records fall within a biome boundary, so that the number of total records for the country may not match the number indicated in the title.

**Fig. 9:**
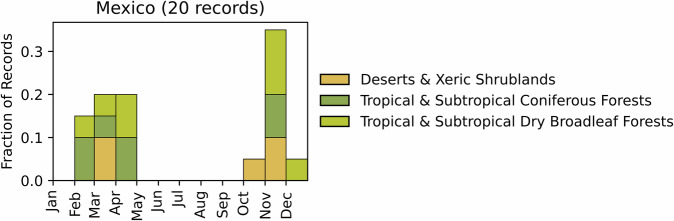
Distribution of RxB records by month of the year for each biome in the country of Mexico. The total number of records falling within a biome are indicated in the title.

**Fig. 10:**
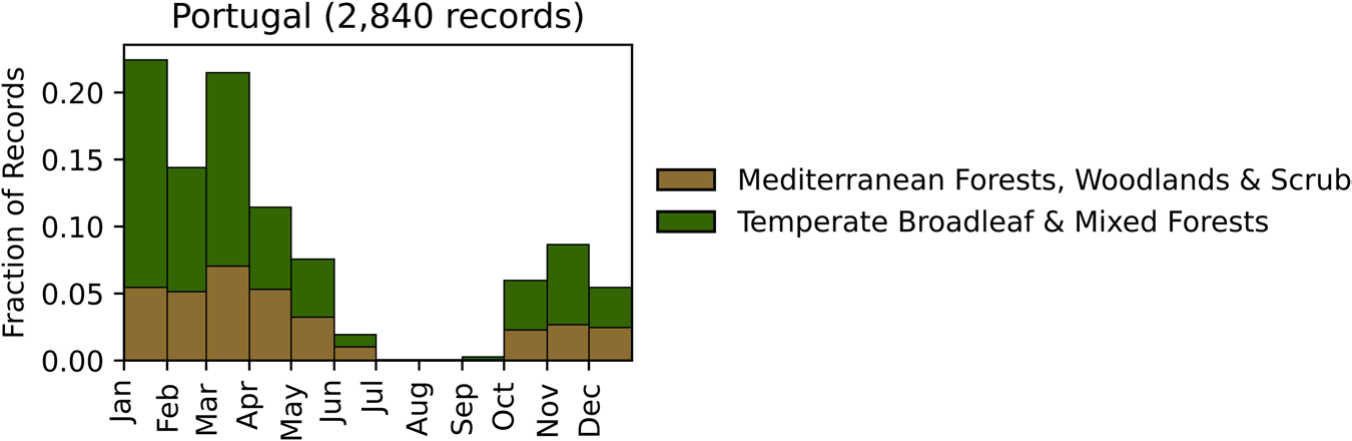
Distribution of RxB records by month of the year for each biome in the country of Portugal. The total number of records falling within a biome are indicated in the title.

**Fig. 11:**
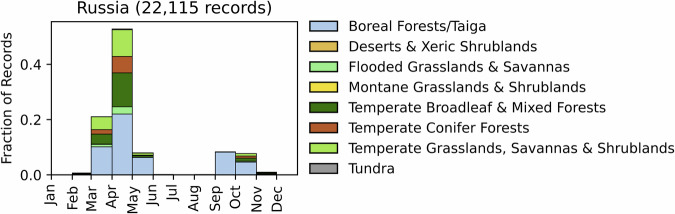
Distribution of RxB records by month of the year for each biome in the country of Russia. The total number of records falling within a biome are indicated in the title. Note that not all records fall within a biome boundary, so that the number of total records for the country may not match the number indicated in the title.

**Fig. 12:**
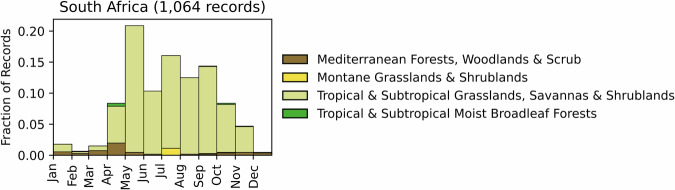
Distribution of RxB records by month of the year for each biome in the country of South Africa. The total number of records falling within a biome are indicated in the title.

**Fig. 13:**
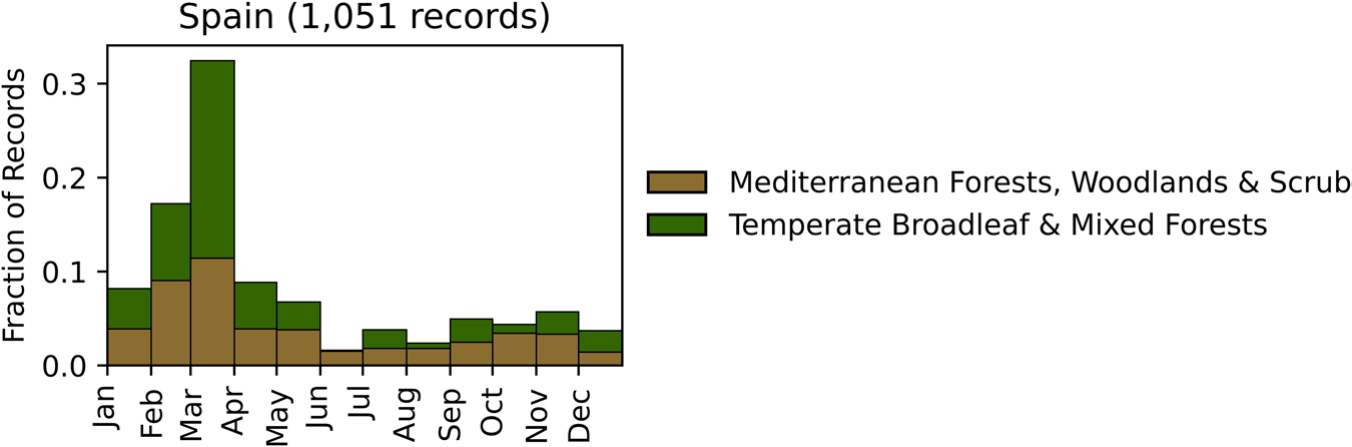
Distribution of RxB records by month of the year for each biome in the country of Spain. The total number of records falling within a biome are indicated in the title.

**Fig. 14:**
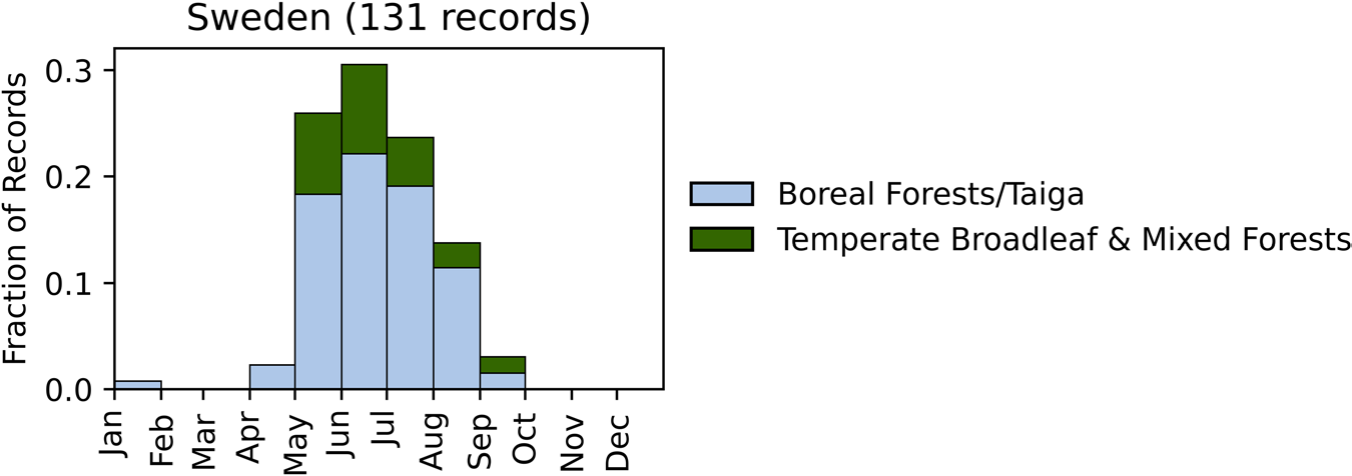
Distribution of RxB records by month of the year for each biome in the country of Sweden. The total number of records falling within a biome are indicated in the title.

**Fig. 15:**
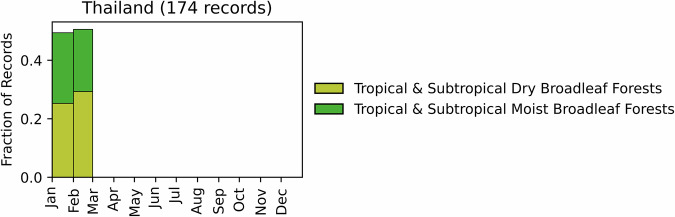
Distribution of RxB records by month of the year for each biome in the country of Thailand. The total number of records falling within a biome are indicated in the title.

**Fig. 16:**
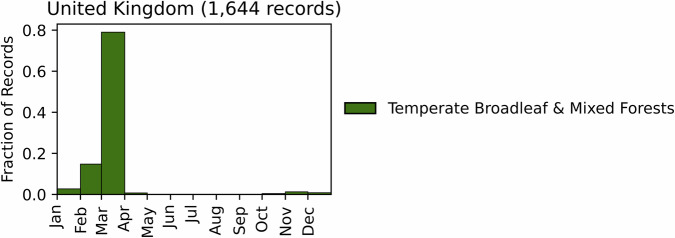
Distribution of RxB records by month of the year for each biome in the country of England. The total number of records falling within a biome are indicated in the title.

**Fig. 17:**
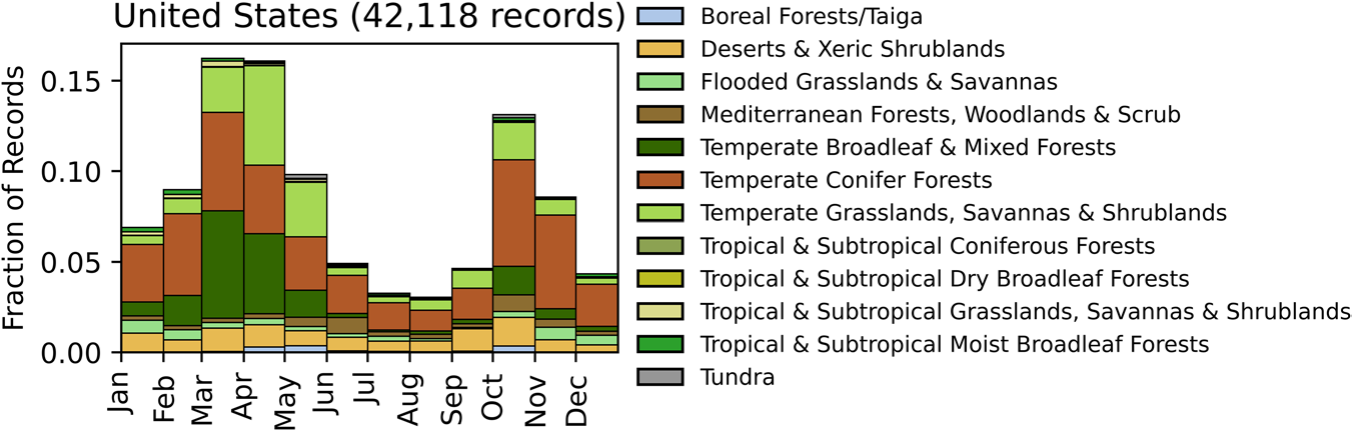
Distribution of RxB records by month of the year for each biome in the country of the United States. The total number of records falling within a biome are indicated in the title. Note that not all records fall within a biome boundary, so that the number of total records for the country may not match the number indicated in the title.

**Fig. 18:**
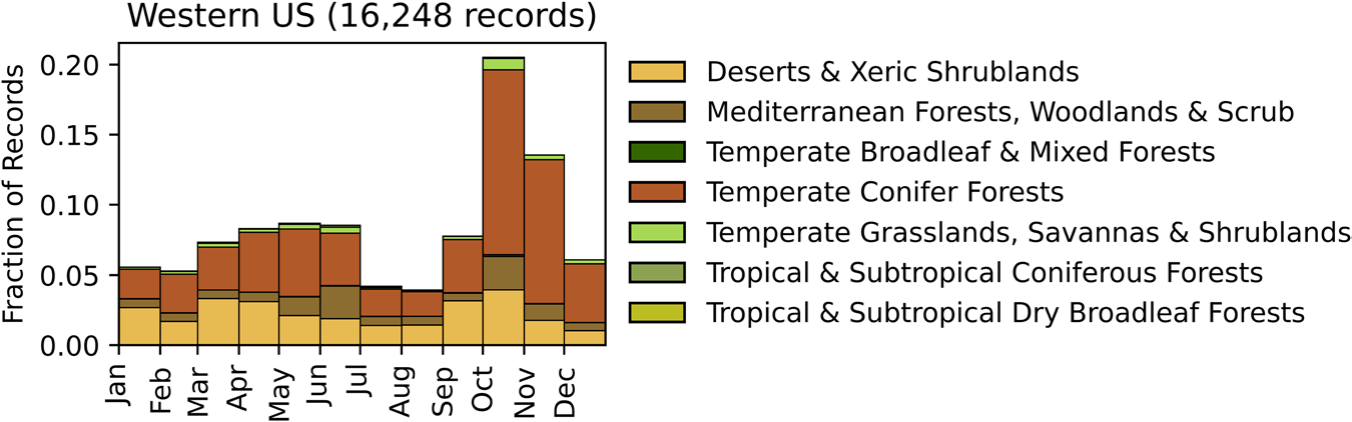
Distribution of RxB records by month of the year for each biome in the Western United States. The total number of records falling within a biome are indicated in the title.

**Fig. 19:**
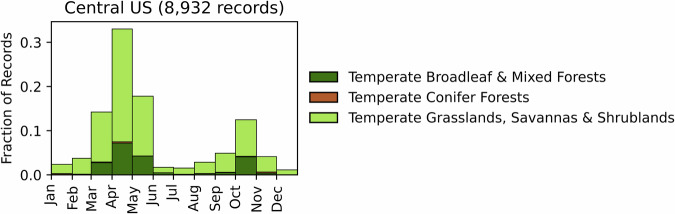
Distribution of RxB records by month of the year for each biome in the Central United States. The total number of records falling within a biome are indicated in the title.

**Fig. 20:**
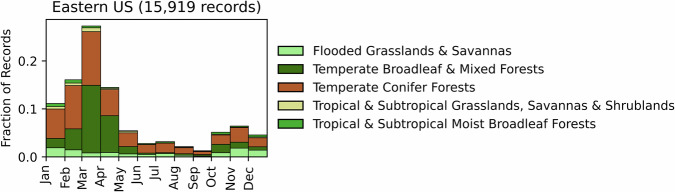
Distribution of RxB records by month of the year for each biome in the Eastern United States. The total number of records falling within a biome are indicated in the title.

**Fig. 21:**
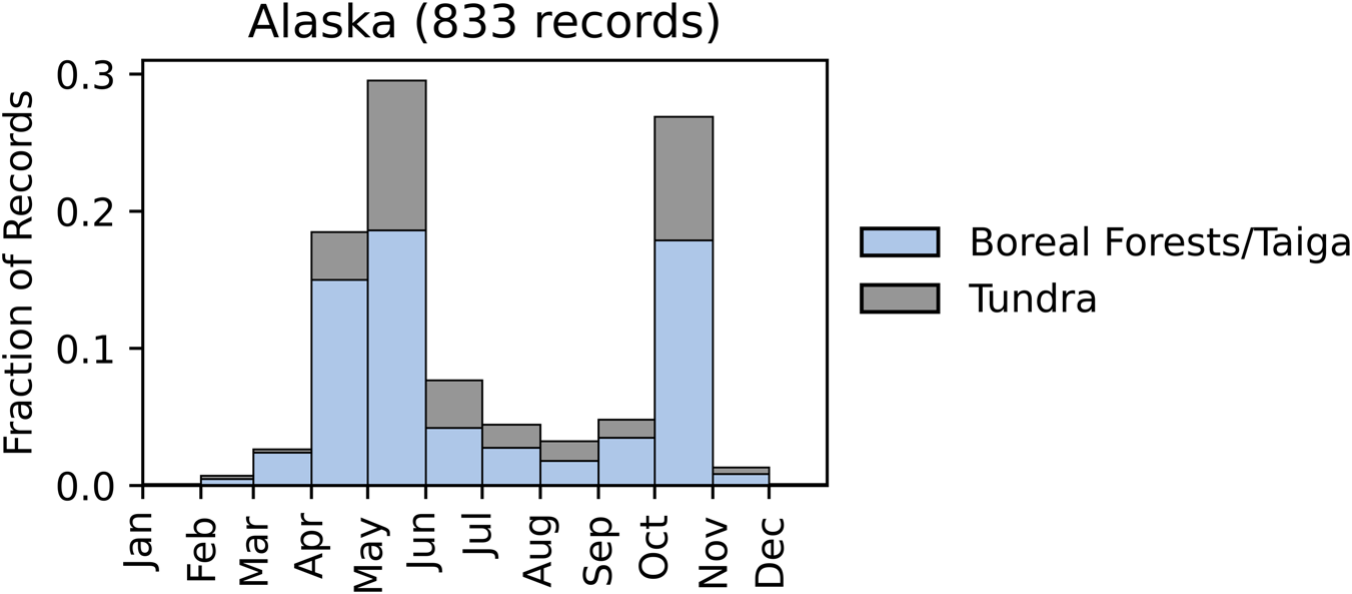
Distribution of RxB records by month of the year for each biome in Alaska. The total number of records falling within a biome are indicated in the title.

**Fig. 22:**
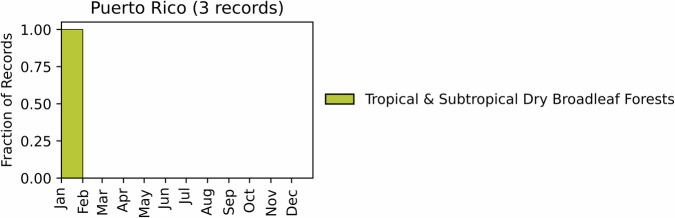
Distribution of RxB records by month of the year for each biome in Puerto Rico. The total number of records falling within a biome are indicated in the title.

Here, we describe a new dataset of 204,517 georeferenced and datestamped RxBs (GlobalRx; see Figs. 1–22) conducted between 1979–2023. GlobalRx is assembled from regional and national databases, described in more detail in Tables 1–3 in the Methods section. The records span 16 countries, 12 biomes, and 209 ecoregions of the world^51^. GlobalRx includes data from public and private repositories maintained by national or state governments, wildland fire management agencies, protected areas such as national parks, and research projects. For each RxB record in GlobalRx, we provide a range of meteorological variables based on the ERA5 reanalysis dataset^52,53^ and information about the environmental setting based on thematic layers (e.g. land cover, ecoregion, protected area status; see Data Records, Tables 2–3). We compiled records starting in the year 1979 so that records could be geolocated (value obtained at location of burn) to meteorological variables in the ERA5 reanalysis dataset. GlobalRx can be used to analyse the proportion of burns falling within RxBWs for different ecosystems and land covers and to compare results across regions and climates.

**Table 1:** Description of variables from burn record information included in GlobalRx (version 2024.1).

Variable Name	Data Type	Unit	Description
ID	String	—	Unique identifier for burn record
Latitude	Numeric	Degrees north	Latitudinal coordinate of the burn, expressed relative to the equator (WGS84 geographic coordinate system)
Longitude	Numeric	Degrees east	Longitudinal coordinate of the burn, expressed relative to the prime meridian (WGS84 geographic coordinate system)
Year	Numeric	Years AD	Date of the burn (Gregorian calendar)
Month	Numeric	Month	
Day	Numeric	Day of the month	
Time	Time	Time of day	Local time of the burn (HH:MM)
DOY	Integer	—	Day of the year (1 through 366)
Date	String	—	String representing date (year, month, and day) on which burn was conducted, in format YYYY-MM-DD
Country	String	—	Country in which the burn was conducted
State/Province	String	—	Administrative region in which the burn was conducted
Agency/ Organisation	String	—	Organisation providing the record
Burn Objective	String	—	Objective(s) of the prescribed burn, if provided (defaults to NaN if not)
Area Burned (Ha)	Numeric	Hectares	Area burned by the prescribed fire
Data Repository	String	—	Link to a public repository hosting the record (if public)
Citation	String	—	Record-specific citation

**Table 2:** Meteorological and fire weather index variables included in GlobalRx (version 2024.1).

Variable Name	Data Type	Unit	Description
T_max, T_mean	Numeric	degrees Kelvin	Daily maximum, mean 2-metre temperature, derived from the hourly 0.25° ERA5 reanalysis product^246^
Wind_max, Wind_mean	Numeric	metres per second	Daily maximum, mean 10-metre wind speed, derived from the hourly 0.25° ERA5 reanalysis product^246^
RH_min, RH_mean	Numeric	%	Daily minimum, mean relative humidity, computed from the hourly 2-metre and dew point temperatures 0.25° ERA5 reanalysis products^246^
PPT_tot	Numeric	metres	Daily total precipitation, derived from the hourly 0.25° ERA5 reanalysis product^246^
BLH_min	Numeric	metres	Daily minimum boundary layer height, derived from the hourly 0.25° ERA5 reanalysis product^246^
FWI	Numeric	Unitless index	Canadian Forest Fire Weather Index (FWI) value, derived from the daily 0.25° ERA5 fire weather product^247^
FFMC	Numeric	Unitless index	The three primary sub-indices of the Canadian FWI (fine fuel moisture code, FFMC; duff moisture code, DMC; drought code, DC), derived from the daily 0.25° ERA5 fire weather product^247^
DMC	Numeric	Unitless index	
DC	Numeric	Unitless index	
FFDI	Numeric	Unitless index	Australian (McArthur) Forest Fire Danger Index (FFDI), derived from the daily 0.25° ERA5 fire weather product^247^
KBDI	Numeric	Unitless index	Keetch-Byram Drought Index (KBDI), derived from the daily 0.25° ERA5 fire weather product^247^
USBI	Numeric	Unitless index	US Burning Index (BI) from the National Fire Danger Rating System, derived from the daily 0.25° ERA5 fire weather product^247^

**Table 3:** Environmental information variables included in GlobalRx (version 2024.1).

Variable Name	Data Type	Unit	Description
Koppen Climate	Numeric	—	One of 30 Köppen-Geiger climate classifications, derived from 1-km Köppen-Geiger historical climate classification maps for 1961–1990 and 1991–2020, depending on the date of the burn record^87^.
Topography	Numeric	Metres	Metres above sea level, derived from the 15 arc-second resolution GMTED2010 global digital elevation model, resampled to 0.0625 degree resolution^92^.
Fuelbed Classification (GFD-FCCS)	String	—	Fuelbed type in the locale, based on the global fuels dataset^81^. The fuelbed code associated with the fuelbed type, found in lookup tables in ref. (^81^), can be used to collect parameters (e.g. fuel loads and their distribution across six fuel strata) used in fire behaviour and emission modelling.
Biome (Olson)	String	—	Biome in which the burn is located, derived from the Olson Terrestrial Ecoregions of the World product^51^.
Ecoregion (Olson)	String	—	Ecoregion in which the burn is located, derived from the Olson Terrestrial Ecoregions of the World product^51^.
WDPA Name	String	—	Name of the protected area from the World Database on Protected Areas in its original language^83^
WDPA Governance	String	—	Governing body type from the World Database on Protected Areas^83^. Examples: national ministry or agency; regional ministry or agency; Indigenous land.
WDPA Ownership	String	—	Individual, organisation, or group that holds legal ownership of the land or under management^83^. Examples: State, Communal, Individual landowners, For-profit organisations, Non-profit organisations
WDPA Designation	String	—	Designation given by the governing body from the World Database on Protected Areas^83^. Examples: national park; nature reserve.
WDPA IUCN Category	String	—	Protected Areas Management Category from the World Database on Protected Areas, as recognised by the International Union for Conservation of Nature (IUCN)^83^. Codes: Ia – Strict nature reserve; Ib – Wilderness area; II – National park; III – Natural monument or feature; IV – Habitat/species management area, V – Protected landscape, VI – Protected areas with sustainable use of natural resources.

GlobalRx can facilitate the exchange of harmonised meteorological and environmental data, streamlining the planning and evaluation of RxB across similar ecosystems under future climate. The database also complements other efforts to quantify and parameterise global anthropogenic fire impacts that have been made through the development of the database of anthropogenic fire impacts (DAFI) and livelihood fire database (LIFE)^54,55^. Together, these databases strive to provide critical new information for evaluating regional variations in fire use practices, evaluating the impacts of climate change on human fire use, as well as for training regional to global-scale fire models to better represent the effects of human fire use on land surface processes^54–56^.

## Methods

### Assembling burn records

#### Data acquisition

The sources of all national or regional datasets contributing to GlobalRx are listed in Supplementary Table 1. We obtained records from public repositories where available (see references in Supplementary Table 1), and otherwise submitted data requests to the providers identified in Supplementary Table 1.

The minimum requirements for inclusion of an RxB record in the GlobalRx dataset were geolocation data (latitude and longitude, along with information regarding the geographic or projected coordinate system) and a record of the day on which the burn was conducted. All data were provided as either a vector dataset (e.g. ESRI shapefiles) or a tabular dataset (e.g. Excel spreadsheets). The data from all sources listed in Supplementary Table 1 were parsed into a common tabular format with fields as described in Tables 1–3.

Two supplementary variables were also parsed from the national or regional records in the cases where they were recorded in the source data: the area burned by the RxB was recorded for 192,179 records spanning all countries except Brazil and the United Kingdom. The primary objective of the RxB was available for 112,397 records spanning select burns in Australia, Brazil, Italy, Portugal, Russia, and the USA (69% of all records).

#### Harmonisation

To ensure consistency in the format of the GlobalRx record across all regions, we applied the following transformations to the data where necessary.

##### Geographical projection

The coordinates of all records were reprojected to the WGS84 geographic coordinate system if necessary using the project tool from the Python GeoPandas package version 0.9.0.

##### Ignition geolocations

In some cases, the data provided by sources in Supplementary Table 1 were retrieved in an ESRI polygon shapefile format mapping the boundary of each RxB. In these instances, the burn geolocation was approximated as the geometric centroid of the area burned by an RxB as derived using the Python GeoPandas package version 0.9.0. The North Australia and Rangelands Fire Information (NAFI) dataset provided records for the Northern Territories in Australia as ESRI line shapefiles, in which case the midpoint of each burnline was selected manually for each fire.

##### Problematic geolocations

All RxBs with coordinates falling outside of their origin country’s borders were individually inspected. We applied corrections to coordinates where the error could be determined, which included cases for which the latitude and longitude were swapped, where there was a missing negative sign, or where the decimal point was placed one place off (e.g., −6.023 instead of −60.23). Burn records were kept if these corrections resulted in the burn falling within the country and subregion listed in the original record. Burns falling into subregions inconsistent with the listed subregion (e.g., correct country but incorrect state) were corrected using the same methods described above and kept only if consistent with the remainder of the burn location information. Burns for which coordinates could not be corrected and which fell within bodies of water were excluded from GlobalRx. In some cases, RxB records originating from within a certain country were located outside of that country’s domain. However, these points were inspected and found to be located in national parks or protected areas spanning national borders. In these cases, the points were kept in the dataset. This applied to burn records from the province of Ontario in Canada, which includes 1 point in St. Lawrence Islands National Park located in New York, and Brazil, which includes 33 points from the Guiana shield region located in Venezuela.

##### Duplicates

Duplicates were filtered out by identifying and eliminating points for which the Latitude and Longitude (rounded to the nearest 0.001°) and date were the same. For the United States USGS dataset (ref. ^57^), which provided ESRI polygons and also included subsets of the Monitoring Trends in Burn Severity (MTBS) and National Fire Plan Operations and Reporting System (NFPORS) datasets, potential duplicates across datasets were also filtered by identifying any records that fell within the mapped burn polygons on the same recorded dates.

##### Burn dates

For multi-day RxBs, the registered date of the burn in GlobalRx is the date of first ignition. RxBs with timings that could not be reconciled with the period of record, such as those erroneously logged as occurring in future years, were also excluded from GlobalRx.

##### Burned areas

All burned area data, where available, were standardised to the common unit of hectares. It should be noted that burned area data may in some cases be an overestimate of the actual area burned, since the recorded burned area value may be for an entire plot approved for burning, though only a portion of a plot may be treated. Similarly, in areas where achieving a mosaic pattern on the landscape is the objective, such as in Australia, the actual burned area will also be less than the total treatment area, since only patches within the entire treatment area are burned.

##### Record selection

When collating data from public fire records (e.g., fire history datasets which include wildfires and intentional fires), all records that were tagged as any kind of intentional fire (“prescribed”, “controlled”, “prescribed fire”, “prescribed burn”, “controlled burn”, “slash”, “agriculture”, and similar variants) were kept.

### Acquisition of meteorological conditions for each burn

We recorded the value of the underlying meteorological components most commonly used to compute the fire danger indices, including 2-metre temperature, relative humidity, daily accumulated precipitation, and wind speed. We also record the value of boundary layer height, as this is commonly used as a metric to ensure adequate smoke dispersion during RxBs. Typically, local noontime of these variables are used to calculate the fire danger indices. However, we accessed the daily maximum temperature, daily minimum relative humidity, maximum wind speed, and minimum boundary layer height, as these minimum and maximum values would set the most conservative limit on prescription burn windows. All variables were also accessed from the ECMWF ERA5 meteorological reanalysis^53^.

In addition to fire weather or fire danger indices, other metrics of the meteorological controls on landscape susceptibility to fire have also been used in wildfire research applications during recent years, including vapour pressure deficit (VPD), which has proven to be an effective predictor of fire incidence in some regions^58,59^, and the Continuous Haines index (CHI), a metric of atmospheric instability and smoke plume transport into the mid- and upper- troposphere^60,61^. These variables were computed from the ECMWF ERA5 meteorological reanalysis and included in the dataset. More detailed descriptions can be found below. For each burn, the meteorological value with the latitude and longitude closest to the burn’s coordinates (determined by minimising the differences between the ERA5 and burn latitudes and longitudes, respectively) were recorded.

### Fire weather and fire danger indices

Fire weather is defined as weather conditions under which fire growth and ignition are favourable - typically when the weather is hot and dry. Fire danger describes the risk of a fire starting and spreading on a landscape, and is typically quantified using measurements of fire weather^62^. Indices of fire weather and fire danger were developed in various world regions as a means of rating flammability of a landscape and to rate daily fire weather and fire danger under the current meteorological conditions. These indices integrate the effects of multiple meteorological variables on the overall readiness of landscape fuels to burn. Fire weather or fire danger indices have occasionally been used in research settings to analyse the window of opportunity for prescribed burning and how it is changing on regional scales^36,46^. The indexes used most widely for research purposes include the Canadian forest fire weather index (FWI), the Australian (McArthur) forest fire danger index (FFDI)^63,64^, and the burning index (BI) of the US national fire danger rating system. All of these indices are functions of fuel moisture and fire weather.

For each RxB, we recorded the value of the most widely-applied indices, including the Canadian FWI, the BI of the US NFDRS, and the Australian FFDI. We accessed these variables from the Copernicus Emergency Management Service (CEMS) historical fire weather indices dataset derived from the ERA5 reanalysis product^52^, which is among the most prominent reanalysis products used in global analyses of fire weather or fire danger^42,43,59^. The fire weather or fire danger ratings were accessed at a spatial resolution of 0.25° and a temporal resolution of 1 day. For each burn, the fire weather index or subcomponent value with the latitude and longitude closest to the burn’s coordinates (determined by minimising the differences between the ERA5 and burn latitudes and longitudes, respectively) were recorded.

#### Canadian fire weather index (FWI)

FWI is the top-level index of the CFFDRS that was developed in the 1970s by the Canadian Forestry Service by unifying various fire danger systems that had been implemented by local or regional wildland fire agencies across Canada. The FWI is calculated from a pyramid of sub-indices. The three primary sub-indices (FFMC, DMC, DC, described below and also included in the dataset) represent the moisture content of specific forest floor layers (fine fuels, duff and organic soil) as a function of temperature, relative humidity, precipitation, and wind speed. Two intermediate sub-indices, the initial spread index (ISI) build-up index (BUI), are calculated by combining the primary sub-indices and wind speed, and represent the potential fire behaviour rate of fire spread and fuel consumption, respectively. The FWI index, calculated by combining the intermediate sub-indices, represents the fireline intensity^65^. The parameters used in the calculation of each sub-index and their combination have been optimised to explain variability in observations. The CFFDRS system was originally developed for application in mature, closed-canopy pine forests. However, all input variables are climactic only, which enables its application on the global scale, regardless of fuel type^66^.

#### Fine fuel moisture code (FFMC)

The Fine Fuel Moisture Code (FFMC) is a numeric rating of the moisture content of litter and other dead fine fuels, such as small twigs, leaves, needles, grasses, or other small diameter material^67^. It is one of the subcomponents used to compute the FWI. The rating is an indicator of the ease of ignition and flammability of fine fuels and is a function of temperature, relative air humidity, wind speed and noontime precipitation. The FFMC is bounded between 0 and 99, and fine fuels are generally considered flammable above a value of 70^68^.

#### Duff moisture code (DMC)

The Duff Moisture Code (DMC) is a numeric rating of the moisture content of loosely compacted, decomposing organic matter. It is one of the subcomponents used to compute the FWI. It assesses fuel consumption in moderate duff layers and medium-size woody material at mid-afternoon and is a function of temperature, relative air humidity, noontime precipitation, and the current month in order to take daylength into account. It reacts more slowly to weather changes compared to the FFMC and is needed to account for the amount of moisture lost daily by slow drying fuels, which is as much dependent on the time available as on noontime atmospheric conditions^66^.

#### Drought code (DC)

The Drought Code (DC) is a numeric rating of the moisture content of the deep layer of compact organic matter. It is one of the subcomponents used to compute the FWI. It assesses the effects of seasonal drought on deep duff layers and heavy fuels and is a function of noontime temperature, precipitation, and the current month. The DC reacts the slowest among the three primary sub-indices to weather changes and captures long-term drought effects^66^.

#### US fire danger rating system burning index (BI)

The BI is one of four outputs from the US National Fire Danger Rating System Burning Index (NFDRS)^68^. The NFDRS was developed in the early 1970s and unified several rating systems in use across the US at the time. The NFDRS is designed to be applicable to every part of the US, but adaptable to the needs of local managers. The system is based on semi-empirical parameterisations that capture the relationships between fuel types, weather, topography and fire behaviour^69^. NFDRS computes three sub-indices representing ignition probability, rate of spread, fireline intensity, and difficulty of control. The sub-indices represent dead and living fuel moisture conditions and are determined by relationships with temperature, relative humidity, precipitation, wind speed, solar radiation, vapour pressure deficit, day length, precipitation, topographic slope, and fuel type. The BI is then computed from the sub-indices in an optimised manner. In contrast to the CFFDRS, the NFDRS is fuel-type dependent and so allows for selection of ecologically-appropriate fuel models to determine the distribution of fuel across different fuel classes.

#### Australian (McArthur) forest fire danger index (FFDI)

The McArthur Forest Fire Danger Index (FFDI) was developed in the 1960s as a measure of fire danger in eucalypt forests of eastern Australia^63^. The FFDI equation is an exponential function of temperature, relative humidity, wind speed, and a drought factor based on the Keetch-Byram Drought Index (KBDI).

#### Keetch-byram drought index (KBDI)

The KBDI is an estimate of soil moisture deficit, defined as the amount of water necessary to bring the soil moisture to its full capacity. It indicates fuel availability for combustion and on any one day is a function of its value on the previous day, temperature, and rainfall^70^.

#### Vapour pressure deficit (VPD)

VPD is a key factor controlling evaporative demand and vegetation drying. It is the difference between the vapour pressure (hPa) held by air at a given temperature and relative humidity, and the vapour pressure (hPa) of saturated air at the same temperature (i.e. vapour pressure at 100% relative humidity). Increased VPD leads to an increased moisture gradient between the atmosphere and vegetation tissues, enhancing evaporative demand and promoting greater rates of transpiration and vegetation drying^71,72^. Hence VPD is a relatively straightforward measure of the impact of temperature and humidity on vegetation dryness and thus readiness for combustion. For some regions without significant limitations to fuel quantity, VPD has proven a strong predictor of wildfire occurrence^27,73^. Unlike other indices, VPD does not depend on antecedent conditions and therefore may not necessarily approximate fuel moisture conditions when observed at a single time point^73^. VPD was calculated at 3-hourly timesteps using 2m temperature and 2m dew point temperature from ERA5 at 0.25° resolution^53^ Actual vapour pressure (e_a_) and saturation vapour pressure (e_S_) were calculated following ref. ^74^ from dew point temperature and temperature, respectively, and VPD was calculated as e_S_-e_a_. The daily maximum value of VPD at the geolocation of each RxB record was appended to the GlobalRx dataset.

#### Continuous haines index (CHI)

The CHI^60^ is an index that measures the potential for dry, unstable air to rise and therefore to promote large, erratic fires^61^. Higher CHI values indicate a higher potential for the uplift of smoke, thus affecting regional air quality^75^, and embers, thus raising the likelihood of downwing spotting ignitions^76^. In addition, higher CHI values indicate a higher potential for plume-driven fire behaviour, including the formation of pyrocumulonimbus, which can also lead to additional ignitions through the occurrence of lightning^77^. Due to the potential for unwanted fire spread and impacts on regional air quality, RxB is typically avoided at high CHI values^78^. The calculation of CHI combines a stability term, the difference in temperature between two atmospheric levels, and a moisture term, the difference between the ambient and dewpoint temperature at the upper atmospheric level^60^. CHI was calculated at 3-hourly timesteps using temperature at 750 and 850 hPa and dewpoint temperature at 850 hPa from ERA5-Land at 0.25° resolution, following ref. ^79^. We use these pressure levels as an approximation of the variables at a global average elevation. The CHI was calculated following the formulas provided by Mills and McCaw 2010 and employed in numerous studies of extreme fire^49,61,80^. The daily maximum value of CHI at the geolocation of each RxB record was appended to the GlobalRx dataset.

### Acquisition of the environmental setting of each burn

For each burn record, we also obtained the value or classification of several thematic layers at the location of the burn. These thematic layers include terrestrial ecoregions and biomes, fuel bed classification, protected area status, climate zone, and topography. These layers are useful for contextualising the environment of the prescribed burn, as well as obtaining information pertaining to the fire weather conditions under which the burns were conducted. More details on the thematic layer data sources, data layer processing and calculation, and geolocation can be found in the sections below.

We highlight that these data derive from global thematic layers and broadly relate to the characteristics of natural vegetation; however, they may not necessarily reflect the specific land cover or ecosystem that was burned.

#### Biome and ecoregion

We identified the biome and ecoregion for the location of each RxB based on the Terrestrial Ecoregions of the World (TEOW) dataset^51^. The dataset was produced by biogeographers, taxonomists, conservation biologists, and ecologists for the World Wildlife Foundation. It maps 14 biomes, which are distinguished by climate (e.g. tropical versus temperate), dominant plant form (e.g. forest versus grassland) and plant traits (e.g. deciduous versus coniferous). Further, the dataset includes 867 ecoregions, which distinguish units of finer-scale floristic or zoogeographic variation within biomes based on existing regional classification systems and consultations with over 1000 regional experts. The biome and ecoregion data from the TEOW were appended to each record in GlobalRx using the spatial join function in the GeoPandas package in Python.

#### Global fuelbed classification

We identified the fuelbed classification of the fuel characteristic classification system (FCCS) for the location of each RxB, based on the global fuel dataset (GFD)^81^. The FCCS distinguishes wildland fuel characteristics and is used in fire behaviour and emission models to predict surface fire behaviour (e.g. spread rates) and crown fire potential. Each fuelbed presents a distinctive structure and composition of wildland fuels and thus shows a distinctive fire behaviour^82^. The GFD maps fuelbeds globally for use in models employing the FCCS system. The fuelbed map was produced by combining biome information from the TEOW dataset^51^ with observations of land cover from the GlobCover 2005 V2.2 product and MODIS vegetation continuous field (VCF) Collection 5 for the year 2005. In addition, the GFD provides FCCS-compliant fuelbed parameters, including fuel loads and their distribution across six fuel strata. Parameters were inherited from regional fuelbed datasets where available, or cross-referenced from regional datasets based on biome, species composition, and tree canopy cover and height. All tiles of the fuelbed classification map were consolidated into a single file, resampled to 0.02 degree, and then saved as a netCDF file resolution using QGIS. The fuelbed classification from the GFD maps were then appended to each record in GlobalRx by determining the fuelbed code with the latitude and longitude closest to the burn’s coordinates (determined by minimising the differences between the GFD and burn latitudes and longitudes, respectively). The fuelbed code was then referenced to the lookup tables provided by ref. ^81^ to determine the fuelbed classification.

#### Protected area status

We identified the land protection status for the location of each RxB with classifications based on the World Database on Protected Areas (WDPA)^83^, which is a joint initiative of the IUCN and United Nations Environment Programme and World Conservation Monitoring Centre^84^. The IUCN categories include a range of strict (i.e. non-use) protection categories (Ia, Ib, II, II) and protection classes that include traditional peoples and Indigenous Communities (IV-VI). See ref. ^85^ for complete definitions of the categories and Table 3 for brief details. The dataset was downloaded from the May 2024 update of WDPA^83^. The data were appended using the spatial join function within the Python GeoPandas package. We included the name of the protected area (in original language), the governance type (e.g. national ministry or agency; regional ministry or agency; Indigenous land), the national or regional designation of the protected area (e.g. national park or nature reserve), and the protected areas management category as recognised by the International Union for Conservation of Nature (IUCN).

#### Climate classification

The Köppen-Geiger climate classification is a well-known and widely used climate classification system developed by Wladimir Köppen and later refined by Rudolf Geiger. The classifications are designed to align with ecosystem and vegetation types world wide, and thus provide a useful insight into the RxB environment^86^. The classification system is based on threshold values and seasonality of monthly air temperature and precipitation, and divides the world’s climate into five major classes and 30 total sub-classes^87^. For each burn, we identified the Köppen-Geiger climate classification using the 1-km Köppen-Geiger historical climate classification maps from 1961–1990 and 1991–2020, depending on the date of the burn record^87^. The classification maps were read into Python as netCDFs, and we extracted the climate classification datapoint closest to the location of the burn.

#### Elevation

Elevation is an important environmental parameter affecting fire regime, vegetation type and distribution, and soil properties^88,89^. As such, it is an important determinant of natural resource management and land use^90,91^. For each burn, we identified the elevation, in units of metres above sea level, from the 15 arc-second resolution GMTED2010 global digital elevation model, resampled to 0.0625 degree resolution^92^. The elevation maps were read into Python as netCDFs, and we extracted the elevation value closest to the location of the burn.

## Data Records

The GlobalRx dataset is available in 3 formats via the Zenodo repository^93^:

1. Comma-separated values (CSV) format (GlobalRx_v2024.1.csv; 175 MB).
2. Microsoft Excel *.xlsx* format (GlobalRx_v2024.1.xlsx; 70 MB).
3. ESRI point shapefile (GlobalRx_v2024.1.shp, with accompanying.shx, .dbf, .prj and .cpg files; 1 GB).

In addition, tabular summaries of the Rx burn counts are available via the Zenodo repository as follows:

1. Per country and biome (summary_table_country_biome_GlobalRx.xlsx; 7 KB).
2. Per country and fuelbed type (summary_table_country_fuelbed_GlobalRx.xlsx; 14 KB).
3. Per country and Rx burn size class (summary_table_country_burnsize_GlobalRx.xlsx; 6 KB).

A description of all variables included in GlobalRx_v2024.1.xlsx is provided in Tables 2, 3. The information contained within the attribute table of GlobalRx_v2024.1.shp is identical to that of GlobalRx_v2024.1.xlsx. The current version of the dataset contains 204,517 records of individual RxBs (Fig. 1).

## Technical Validation

Our technical validation consists of two phases. Firstly, we evaluate the distribution of burns across land covers, ecoregions, and seasons on national and regional bases to assess representation, and compare these patterns with the available regional literature. This informs our qualitative assessment of the representativeness of GlobalRx on national scales and provides opportunities to identify likely data gaps in cases where the distribution and quantity of data are not consistent with the literature. Supplementary Text S1 provides a more detailed description of fire and land management history, fire ecology, and fire regime for each country to further contextualise the role of RxB and other controlled fire uses in relevant regions of that country.

Thereafter, we assess how the values of meteorological variables and fire weather and danger indices in GlobalRx compare with permissible ranges for Rx burns based on the regional legislation or practice guidelines used in burn plans through a series of case studies using burn records from Australia, Portugal, and Sweden. These case studies enable readers to assess how representative the recorded meteorological values are of actual RxB conditions in different environment types and also provide a range of validation plots to suit a range of usage cases, recognising that each end user may have a different motivation for accessing the dataset as well as diverse regional foci.

### Country profiles

#### Australia

GlobalRx contains 120,696 records from Australia spanning 1979–2023. Burn records were collected from each state (New South Wales^94^: 7041; Northern Territory^95^: 344; Queensland^96^: 8,302, South Australia^97^: 1,377; Tasmania^98^: 2,067, Victoria^99^: 89,576; Western Australia^100^: 11,989), from each state-level agency that manages RxB (Supplementary Table 1). All records, excepting those from the Northern Australian Fire Information site, are publicly available on each state agency website. Burns were conducted in every state of the country, covering tropical and subtropical rainforests and grasslands, mediterranean, desert, and montane shrublands, and temperate forests and grasslands. The majority of records (96,711 records, 80%) come from the southeast states (New South Wales, Victoria), where the country’s highest population densities coincide with some of the most flammable landscapes in the world^101^. The majority of these burns was conducted in temperate eucalypt forests and mallee woodlands. The burn objective, which was recorded for Western Australia and Victoria, was predominantly hazardous fuel reduction (Victoria: 82,477; Western Australia: 2,700) followed by burns conducted for ecological management (Victoria: 4,359 burns; Western Australia: 1,056 records) and silviculture (Western Australia: 1,222 records). Burns were conducted predominantly in austral fall (February-May) and spring (August-November), with some regional variation, as indicated in Fig. 2. The majority of ecological, agricultural, and silvicultural burns occur in the spring.

The burn objectives are not available for records outside of Western Australia and Victoria. However, hazardous fuel reduction is the most common purpose for RxB. Ecological management objectives are also prevalent, especially to create or maintain patch mosaics of vegetation that aim to replicate Aboriginal firing patterns that were forbidden and displaced under colonisation^102–104^. In the Northern Territory, RxB is commonly used for hazardous fuel reduction in the tropical savanna biome, and is also a part of a carbon sequestration scheme on predominantly Aboriginal territory^105,106^. These projects also increase Aboriginal stewardship of the landscape, support the economic independence and livelihoods of participating Aboriginal communities, and may serve as a potential template for post-colonial land management^107,108^. Aboriginal fire expertise is also preserved today through Aboriginal fire management programs and companies, such as the FireSticks Alliance and Arnhem Land Fire Abatement (Northern Territory) Limited^107,109^.

#### Brazil

GlobalRx contains 9,873 burn records from Brazil spanning from 2015–2020. All records originate from the Brazilian Institute of Environment and Renewable Natural Resources (IBAMA)/National Center to Prevent and Combat Forest Fires (PREVFOGO)^110^, the Brazilian Ministry of the Environment’s administrative arm which implements laws within federal protected areas and Indigenous territories. RxB is only legal within these units (i.e. of Indigenous lands management under PREVFOGO, and in protected areas and sustainable use reserves, managed by ICMBio) and no preventative burns are legal in the rest of the country’s territory. Records were collected from 7 of the 26 states of Brazil and include burns conducted on public and Indigenous lands, primarily in the fire-prone Cerrado (6,224 burns, 63%) and in the Mato Grosso seasonal forests (1,637 burns, 17%), which constitute the transition region between the Cerrado and the Amazon rainforest. An additional 1,896 burns (19%) come from the Guianan shield region in the northern state of Roraima, where there are active participatory research and governmental efforts to implement an IFM system within the Indigenous territories of Raposa Serra do Sol, Canaima National Park, North and South Rupununi to reform zero-fire policies and recognise Indigenous fire knowledge^17,111,112^. Many burn records listed multiple objectives, and among these hazardous fuel reduction was a listed objective in the majority of burns (68%), primarily to reduce the risk of late dry season fires, followed by silviculture and agroforestry. The majority of burns are conducted during the peak rainy season between April and August (Fig. 3), except in Roraima, where burns are conducted predominantly during the early dry season, between October and January^113^.

#### Canada

GlobalRx contains 557 burn records from Canada spanning 1983–2022. All records come from Natural Resources Canada (130)^114^ and Parks Canada (427)^115,116^ and include burns conducted only on public lands, namely national and regional parks and wildlife areas. We note that most RxB programs are managed on a provincial or regional level in Canada; however, these data were not accessed, though these constitute the vast majority of RxB that occurs in the country. These regional and provincial burns are conducted primarily for hazardous fuel reduction^117–119^. A significant number of RxBs are also conducted for silviculture, but these data are also not included in GlobalRx, as these are not recorded in government databases we accessed^120^. Burn objectives were not available from the records; however, the majority of burns in the dataset were conducted in national parks, where RxB is used for the ecological restoration of forests and grasslands, to enhance habitat for wildlife, and reduce the risk of wildfire in adjacent communities^121^. Records cover every province except Yukon, though the majority come from burns in the ecoregions near the Rocky Mountains. Burns are conducted primarily in the spring (March-May), and to a lesser extent in the fall (August-October) (Fig. 4), as this confers the most benefits for wildlife habitat and forage and also coincides with favourable weather conditions for burning (Fig. 4)^120,122^.

#### France

GlobalRx contains records of 1448 burns in France spanning 1983–2016^123^. Records were obtained through the National Research Institute for Agriculture, Food, and Environment and only include burns conducted in the Pyrénées-Orientales, an administrative region in Southeast France that lies on the eastern, Mediterranean side of the Pyrenees. The Pyrénées-Orientales hosts the oldest RxB team in France, whose formation was spurred by catastrophic fires in 1976 and 1986^32^. RxB in France is managed by local actors and government agencies individually in each administrative division, with occasional support from civil protection units of the French Army^25^. In the Pyrenees region RxBs are used mainly to achieve a combination of pastoral management and hazardous fuel reduction. The Pyrénées-Orientales division accounts for an average of 25% of the RxB area burnt in the country^25,124^. Burns in this region are conducted by the Pyrénées-Orientales burning team from October to April, with the number of burns peaking in the month of February, as indicated in Fig. 5.

#### Germany

GlobalRx contains 3 burn records^125^, all conducted in March 2019 in the Zschornoer Wald nature reserve located in the state of Brandenburg. This nature reserve is located in the Central European mixed forests ecoregion. Records were obtained from the German Federal Real Estate Administration through personal communication. Separately, thousands of records of RxB in Germany, and their specific ecological context (e.g. fire return intervals), burn plans and objective outcomes, are maintained by the Global Fire Monitoring Center (GFMC)^126,127^; The GFMC records may also prove a useful resource to researchers studying RxB, however they could not be integrated into GlobalRx at present because they lack geolocation or timing data.

The use of RxB in Germany goes back to the 1970s^128^. Over the last 30 years, experimental and management RxB were conducted to preserve cultural landscapes such as meadow grasslands and heathlands through the reduction of woody vegetation, predominantly in continental and coastal dune heathlands, and some forest plantations and peat bogs in Nordrhein-Westfalen^32^, Baden-Württemberg^129,130^, Niedersachsen^131,132^, and Schleswig-Holstein^133^. Special RxB procedures were developed on terrain contaminated with Unexploded Ordnance (UXO) with armoured equipment (military tanks converted to ignition and suppression tanks) in Brandenburg State for disposal of UXO and regeneration of heath^134,135^. Permission for RxB operations must meet the requirements of the Federal Conservation Law and the relevant State laws regulating conservation, forestry, wildfire safety, emissions control and waste disposal as well as local rules of protected areas. The seasonality of RxB varies from winter months to minimise thermal effects to the soil biota. in viticulture areas of Southwest Germany (January-February), early spring in heathlands in East Germany (February-March) and summer in North Germany (post bird-breeding season starting mid-July onwards)^129,136^.

Burns were generally conducted to preserve cultural landscapes such as meadow grasslands and heathlands through the reduction of woody vegetation. Burns were generally conducted in the winter to minimise any thermal effects to the soil^32,129^. In the specific case of the 3 burn records, fires were implemented towards the end of the legal burn window in Brandenburg, which ends April 1 (Fig. 6).

#### Italy

GlobalRx contains 135 burn records from Italy^137^ spanning from 2005–2021. Data were requested from the Italian Society of Silviculture and Forest Ecology (SISEF) to regional fire management agencies, and records include burns conducted on public lands only. Burns were conducted primarily in Mediterranean coniferous forests in regions west of the Apennine Mountains, predominantly in the regions of Tuscany and Campania. RxB in Italy is managed individually by each region. Burns were conducted primarily for hazardous fuel reduction (81%), followed by a combination of fuel reduction and habitat conservation (15%) and fuel reduction and grazing management (4%). An analysis of regional burn plans across Italy conducted by ref. (^138^) showed that wildfire hazard reduction was the most common objective for RxB. Additionally, similar to other Mediterranean European countries, RxB is used for multiple objectives^139^. For example, in Northern Italy, RxB is used alongside grazing in collaboration with local shepherds for the conservation of heathland species^138,140,141^. The majority of burns were conducted from late winter through the spring (January-May), with the most burns occurring in the month of March, and to a lesser extent in the autumn (October-November) (Fig. 7). The burn season is dictated partially by fire weather^138^ as well as air quality restrictions, which restrict burning in the autumn (David Ascoli, pers. comm.).

#### Japan

GlobalRx contains 407 burn records from Japan spanning 1979–2021^142^. Records were requested through Hokkaido University through personal communication. All recorded burns were conducted within the Temperate Broadleaf & Mixed Forests biome, within the Nihonkai and Taiheiyo evergreen and montane deciduous forest ecoregions. The majority of recorded burns (283 records, 70%) were located in the Taiheiyo evergreen forest ecoregion. While records appear to be predominantly located in forested ecoregions, the majority of recorded burns were conducted in grasslands in the southwest of the country, in areas often dominated by *Miscanthus sinensis* (Japanese pampas grass), *M. sacchariflorus* (Amur silver grass)^143^, or *Phragmites australis* (common reed). Prescribed burning is uncommon in Japanese forests as they are not generally fire prone, and the majority of preventative fire measures consist of preventing ignitions, preventative logging, and removal of surface fuels^144^. Burn objectives were not available through the original records; however, in these regions, burning is carried out annually, often in the winter or spring (January - March) (Fig. 8), by local residents as a part of a yearly ritual or tradition to prevent woody encroachment, conserve the grasslands, renew pastures, and to prevent wildfires^145–151^.

#### Mexico

GlobalRx contains records for 20 burns from Mexico spanning 2016–2021^152^, all requested from the Comisión Nacional Forestal (CONAFOR), the federal agency that oversees fire suppression forces and also helps develop and implements fire management policies across the country. The records are sparse but cover 16 of 32 states in three of the largest (out of eight) biomes present in the country, including Deserts & Xeric Shrublands, Tropical & Subtropical Coniferous Forests, Tropical & Subtropical Dry Broadleaf Forests. Burn objectives were not available through the original records; however, RxB in Mexico is commonly implemented through Integrated Fire Management (IFM) programs that combine ecological, silvicultural, and fire risk reduction objectives with the agricultural and resource needs of local communities, often Indigenous or ejido communities^24,153^. Many of these programs are based in biosphere reserves in the southern tropical and subtropical regions such as Chiapas, Oaxaca, and Campeche^154^, though traditional fire use and IFM programs are also prevalent throughout northern and central Mexico^154–156^. The majority of burns are conducted in the late autumn between October and December, following the harvest season, and then in early spring, prior to the growing season (Fig. 9). Note that the obtained records are extremely limited and do not reflect the prevalence of fire management programs in the country (pers. comm., César Robles).

#### Portugal

GlobalRx contains 2,840 records of RxBs in Portugal spanning 2002–2022^157^, estimated to account for 75% of the total number of operations within the period. Records were obtained from the Instituto da Conservação da Natureza e das Florestas (ICNF), the agency through which RxB is managed and recorded nationally^158^, and supplemented by the authors. The recorded burns cover 17 of the 18 districts in the country, but are concentrated in the northwest, which is dominated by maritime pine forests and shrublands. Burn records for the years 2021 and 2022 include information about the burn objectives. RxB is conducted overwhelmingly for hazardous fuel reduction in both natural and plantation forests and shrublands, with pastoral, agricultural, and silvicultural burning comprising a far smaller proportion of burning compared to France or Spain^124^. Most of the RxB activity is carried out in communal land co-managed by ICNF and occupied by oceanic-influenced dry heathland typically dominated by *Erica* and *Ulex* species and *Pterospartum tridentatum*. Recorded burns were conducted primarily between October and May, with the majority of burns occuring in the early spring (February-April) (Fig. 10).

#### Russia

GlobalRx contains 22,142 records of fires in Russia spanning 2008–2020^159^ which are classified as RxB by the authorities. Records were obtained from the Forest Fire Monitoring Information System of the Federal Forestry Agency (ISDM-Rosleskhoz). Burn data in the ISDM-Rosleskhoz database was originally collected from MODIS fire detections but has since evolved to include detections of active fires from the Himawari-8, Sentinel 2, Meteor, NPP, and NOAA-20 satellites. The collated data is then reviewed by forest fire service specialists, who validate the data and provide comments on the fire type^160,161^. Burns are planned and at the *Lesnichestvo* level, the Russian territorial unit of forest management, and approved by the *Rosleskhoz*, the Federal Forestry Agency of Russia, which controls and manages Russia’s forests, all of which are state-owned^162,163^. GlobalRx is the subset of the above data classified as RxBs.

Records are most prevalent in the southern border regions of the country, east of Baikal Lake. The records represent mainly fuel reduction burns, which are conducted to prevent the spread of fires from agricultural fields and fires applied around rural settlements into adjacent forests and unforested areas (pers. comm. Elena Kukavskaya). Hence, some burns may also have a fire type classification of *Agricultural*. Agricultural burns are conducted primarily in the spring (March-May), with the greatest number of burns conducted in March and April, and to a lesser extent in autumn (October-November) (Fig. 11). Silvicultural burns (to clear logging slash) and agricultural burns are not included in this data, though these practices are also prevalent^164,165^. The reported RxB data include both controlled burns and traditional burning practices, including “wildfire use fires” (let burn of unplanned wildland fires that meet land and fire management objectives). At present, controlled burns cannot be disaggregated from “wildfire use fires” in the records from ISDM-Rosleskhoz. Decision protocols for RxB application based on scientific evidence of the fire ecology of fire-adapted and fire-dependent forest and non-forest ecosystems in Russia are not yet in common practice^166–169^. However, recommendations have been made for the development of training programs for fire management specialists^170^.

#### South Africa

GlobalRx contains records from 1,065 burns spanning 1979–2021, all requested from the South African National Parks (SANParks) agency^171–173^. All records are from burns conducted in national parks. The majority of burns (975 burns, 92%) were conducted in semi-arid savannas of the Kruger National Park (KNP), where the first RxB experiments were conducted. Burn objectives were not available from the records; however, burns within KNP are primarily conducted for ecological management and research^174^, while burns in savannas both within and adjacent to the park have been used to promote fire’s ecological role, provision of green grazing, and also combat bush encroachment^175–178^. The remaining records are from burns in fynbos and renosterveld ecosystems within the Garden Route and Table Mountain National Parks. RxB within fynbos ecosystems are conducted primarily for ecological conservation, especially that of fire-adapted species^178–180^. The burns in savannas were conducted primarily from austral autumn to early spring (April-November), with the most burns conducted from May to September. Burns in the fynbos shrubland biome were conducted primarily in the austral autumn (March-April) (Fig. 12). Records of RxB use in forestry for reducing wildfire hazard, which are applied in industrial pine plantations, were not available^181^.

#### Spain

GlobalRx contains 1,051 total records of prescribed burns in Spain spanning 1998–2021 and covering four autonomous communities (administrative divisions): Catalonia^182^, Andalusia^183^, Galicia^184^ and Asturias^185^. Records from Catalonia were available publicly through the Catalonian government website^182^. The remainder of the records were obtained through personal communications with contacts in the regional administrations. Nearly half of all burn records come from Catalonia. Burn objectives were not available in the obtained records. However, prescriptions in northwestern Spain (Galicia and Asturias) are carried out for pastoral management (i.e. pasture regeneration and maintenance), and to a lesser extent hazardous fuel reduction objectives^32,124^. In most of Spain prescribed burns are carried out by the Regional Administrations with technical support from the Integral Wildfire Prevention Teams of the Spanish Forest Fire Service (EPRIF). EPRIF is a national program that deploys teams of fire specialists into rural regions of high fire risk to work with local community members to establish a burn program that suits the region’s needs while also minimising wildfire risk. Burns in Catalonia are carried out by Grup de Recolzament d’Actuacions Forestals (GRAF) for hazardous fuel reduction in forests and shrublands, and to a lesser extent for pastoral management^32,124^. Burns were conducted primarily from late winter to spring (January to May), with the most number of burns carried out in March (Fig. 13). However, in recent years, burns in Catalonia are increasingly conducted from September to November due to better results in fuel management and biodiversity (pers. comm., Marc Castellnou).

#### Sweden

GlobalRx contains 134 records of fires in Sweden spanning 2015–2020^186^, covering 2,667 Ha. All records come from burns conducted as a part of the Life Taiga project, a 6-year long EU-funded conservation project active from 2015–2020 to conduct burns in protected areas across Sweden^187^. Records are distributed over the entire country, with the majority of burns occurring east of the Scandinavian Mountains, in temperate conifer and boreal forests/taiga. While records do not contain burn objectives, the Life Taiga project’s objectives are primarily ecological conservation and the protection of biodiversity^187,188^. Burns were primarily conducted from late spring to the summer (May-August), with the majority of burns taking place in May and June (Fig. 14). It should be noted that GlobalRx does not include burns conducted on commercial forest lands, which account for more area burned than that occurring only on natural reserves. Ref. (^189^) examined data from 2011–2015 and found that forestry companies were responsible for 85% of RxB covering 5280 Ha, nearly double that of Life Taiga. However, these burns are not captured in GlobalRx.

#### Thailand

GlobalRx contains 174 records from Thailand, all from the year 2022^190^. All records were obtained from the Department of National Parks, Wildlife and Plants Conservation (Forest Fire Control Division) in the Thailand Ministry of Natural Resources and Environment through personal communication. All burns were conducted in national parks located in the northeast of the country, in the provinces of Chiang Mai and Lamphun, predominantly in the Central Indochina dry forests and Kayah-Karen montane rain forests. These ecosystems consist primarily of Dipterocarp tree species. Burn objectives were not specified for any of the records. However, these burns were conducted primarily to research fire behaviour. RxBs are also commonly conducted for agriculture and resource management by local communities^191^, silvicultural plantation management^192^, and for research on nutrient cycling and emissions^191,193,194^. All burns were conducted in January and February (Fig. 15).

#### United Kingdom

GlobalRx contains 1,644 burn records spanning 1992–2020^195^. All records come from Forestry England, the division of the Forest Commission responsible for managing publicly owned forests in England. All recorded burns were conducted within New Forest National Park, which lies in the English lowlands beech forests ecoregion. Burn objectives were not specified in any records. However, burns are commonly conducted for wildlife habitat management in the heathlands and mires of the park, particularly for ground-nesting birds^196,197^. Burning is also prominent in upland heathlands and moorlands for maintaining different successional stages of *Calluna vulgaris* (heather), which is used to support sheep grazing, maintain game populations of red grouse and red deer, and reduce wildfire risk, primarily on private lands^32,198,199^. Heather burning in the UK is subject to the Muirburn Code in Scotland and the Heather and Grass Burning Act in Wales and England, which define the legal burning season generally from October or November through March or April to protect wildlife during nesting season^200^. This is reflected in the data, with burns beginning in November and being conducted through April (Fig. 16).

#### United States

GlobalRx contains 42,326 records from the United States (US) spanning from 1979–2023. Records were collated from federal databases, including the Monitoring Trends in Burn Severity (MTBS) database^201^, the Fire and Tree Mortality (FTM) database^202^, and the Interagency Fuel Treatment Decision Support System (IFTDSS)^203^. 6,748 RxBs were conducted for hazardous fuel reduction, 79 RxBs were conducted for research, and the remaining records do not specify a burn objective. The US covers a large variety of different environments, with burns occurring in 78 ecoregions and 12 of the 13 Olson biomes (Fig. 17). Owing to this diversity, the history of fire, RxB, and jurisdictional management varies greatly across the US, with notable differences between the Southeast, the Central US, the West, and Alaska^204^.

We note that 85% of all RxBs in the US are managed and conducted at state or regional levels, and more area is prescribed burned in the southeastern US (multiple times over) than the rest of the US combined^204,205^. Although it is known that significant RxB is performed outside of federal land in the US, georeferenced records are not available publicly for these burns and hence they were not included in GlobalRx. Hence, GlobalRx records for the USA are known to be spatially and ecologically skewed towards regions and biomes where the most federal lands are, primarily in the western US.

#### Western USA

43% (16,311) of records come from the Western US (defined as all RxB not falling within the Great Plains and Southeast and Atlantic regions and encompassing all of Arizona, California, Idaho, Nevada, Oregon, Utah, and Washington, and portions of Colorado, Montana, New Mexico, Texas, and Wyoming), spanning 1979–2023. Burns were conducted primarily in Temperate Conifer Forests (56%, 9,085). A significant portion of RxBs in this biome (2,604, 29%) are conducted for hazardous fuel reduction and target ladder and surface fuels, a common practice near the WUI^47,206,207^, particularly after 2003, when the Healthy Forests Restoration Act (P.L. 108–148) was passed, explicitly tying funding for RxBs to hazardous fuels reduction. 59 burns (1%) of burns in this biome were conducted for research, and the remainder of burns had no burn objective specified. However, burns are also commonly conducted for the ecological restoration of fire-adapted species, such as the Giant Sequoia (*Sequoiadendron giganteum*)^208,209^, or entire fire-adapted communities, such as in wetlands, particularly to restore wildlife habitat. Burns are typically conducted from the fall through the winter and into early spring (September-May), in months outside of the typical wildfire season (June-August) (Fig. 18)^23^. Fall prescribed burning in the western USA is primarily pile burning, while spring provides conditions that are more favourable to conduct broadcast burns, but burn days are also limited due to both species protection laws and interannual climatic variability^210^.

28% (4,495) of burns in the Western US occur in Deserts and Xeric Shrublands, predominantly in pinyon-juniper (P-J) woodlands. 27% (1,215) of burns in this biome were conducted for hazardous fuel reduction, to combat increases in tree density that have been observed in this region in the past century^211^. In P-J shrubland and grassland types, prescribed fire may also restore understory communities of shrubs, grasses, and forbs by reducing tree competition^212^, and increase forage production on federal lands widely leased for grazing cattle^213^.

#### Central USA

21% (8,940) of burns in the US occur in the Central US (defined as the regions in Texas, New Mexico, Oklahoma, Missouri, Kansas, Colorado, Nebraska, Iowa, Illinois, Indiana, Wyoming, South Dakota, North Dakota, Minnesota, Montana falling within the Temperate Grasslands, Savannas & Shrublands biome, as well as all of Michigan, Wisconsin, and Minnesota) region. Here, grasslands have been the dominant vegetation for the last 5000–8000 years, with the prevalence of woody plants, particularly Ashe and Eastern redcedar (*Juniperus ashei*, *J. virginiana*), being closely tied with anthropogenic fire^214^. However, the forcible displacement of Plains Indians, fragmentation of the landscape for settlement and agriculture, and overgrazing from the overstocking of domestic livestock, combined with federal fire suppression policies, the Dust Bowl, and human-mediated dispersal and planting of juniper trees from the 1850s–1930s, led to widespread fire exclusion that resulted in radical losses of grasslands^215,216^. In the 1990s, grassroots movements to address the degradation of grasslands and the potential of RxB to restore them led to the formation of the first prescribed burning associations (PBAs). PBAs consist of groups of private landowners and other interested people who form partnerships to pool their knowledge, equipment, and other resources to conduct RxBs^217^.

PBAs are now commonplace across the US, with over 100 across 18 states as of 2022, the majority of which are concentrated in the Plains region^218^. PBAs help facilitate RxBs, particularly on private land, by providing training, resources, and even liability insurance for burns. In the Plains region, preventing juniper encroachment was the most important objective, though burns were also commonly conducted for livestock production, wildlife management, rangeland maintenance^219^. Burns in this region are conducted primarily in the dormant season, in late spring or late fall when lightning ignitions are also less common (Fig. 19), for operational convenience^23^.

#### Eastern USA

38% (15,919) of records in the US come from the Southeastern US (defined as the regions in Alabama, Arkansas, Delaware, Florida, Georgia, Indiana, Kentucky, Louisiana, Maryland, Mississippi, Missouri, New Jersey, North Carolina, Ohio, Oklahoma, South Carolina, Tennessee, Texas, Virginia, West Virginia falling within the Temperate Broadleaf & Mixed Forests or Temperate Conifer Forests biomes), spanning 1984–2023, in areas encompassed by the Atlantic Coastal Plain and southern portion of the Appalachian Highlands. This region is predominantly of the Temperate Broadleaf & Mixed Forests or Temperate Conifer Forests biomes. The majority of wildlands in the Southeast are privately owned, and RxB is widely administered by state and local agencies in partnership with non-governmental organisations (e.g., The Nature Conservancy) and private landowners). Additionally, state-level legislation across the region (such as the 1990 Prescribed Burning Act in Florida) protects landowners’ right to conduct RxBs by mitigating concerns about liability, which is commonly noted as a top barrier to conducting burns^220,221^.

The majority of Southeast burns (82%, 12,982 burns) were conducted in either Temperate Conifer or Temperate Broadleaf and Mixed Forests. RxB is commonly used in conservation and wildlife habitat restoration efforts in longleaf pine (*Pinus palustris*) or mixed longleaf pine/oak stands found in the South Atlantic coastal areas, which include Georgia, Florida, and Alabama, the lowlands of Mississippi and Louisiana, and Texas. 6.2 million hectares of longleaf pine sites, located in “Significant Geographic Areas (SGA)” encompassing protected areas, were burned from 2011–2021 by members of the Longleaf Partnership Council. Hazardous fuel reduction is sometimes an objective of these burns^222,223^. The majority of burns are conducted during plants’ dormant phase, in the fall, winter, and spring (October-April) (Fig. 20), as weather conditions are milder, and it was believed that burning during this period was less likely to impact nesting birds or growing trees^23^.

#### Alaska

2% (833) of records in the US come from Alaska, spanning 2004–2020. The majority of recorded burns were conducted in the Interior Alaska-Yukon ecoregions (84%, 702 burns), primarily in the lowland taiga (505 burns), and to a lesser extent in the alpine tundra (197 burns), located between the Brooks Range in the North and the Alaska Range in the south. South of the Alaska Range, burns were also conducted in the Cook inlet taiga (42 burns), Alaska-St. Elias Range tundra (31 burns), and the Copper Plateau taiga (16 burns). 15% (124) of records listed the burn objective, which were all hazardous fuel reduction. Despite Alaska’s size, there are relatively fewer prescribed burns in the state because fire suppression has been relatively limited, and did not facilitate the fuel build-up that drives hazardous fuel reduction across much of the contiguous US^26^. The burns that do occur are typically conducted to create and maintain fuel breaks in “active suppression zones,” especially in flammable spruce-dominated forests around Alaska native villages, where damage to life and property are greatest^26,224^. The remainder of the records did not specify the burn objective. However, prescribed fires have also been used to manage moose and grouse habitat, as well as for tree regeneration following beetle kill^225,226^. Burns were conducted primarily in fall and spring (Fig. 21), outside the period of higher wildfire activity.

#### Puerto Rico

3 burn records were from Puerto Rico, from burns conducted in 2005 and 2007. All 3 burns were conducted on the south coast of the island, in the Puerto Rican dry forests ecoregion, of the Subtropical Broadleaf Dry Forest biome (Fig. 22). The region’s climate is characterized by the rain shadow of the Cordillera Central mountains^227^. Fire activity is most common in the dry forest ecoregion during the dry season and is exacerbated by exotic grasses, but few native woody species are capable of surviving even low-intensity fire. No burn objectives were listed for these records; however, RxBs have been explored as a way to manage exotic grass patches^228^.

### Prescription window case studies

To verify that the ERA5 meteorological values we geolocated for each burn are representative of the weather conditions under which the burns were conducted, we compiled a range of published RxB weather guidelines from select regions and then examined the extent to which burns fall within these prescription windows. We expect that if the ERA5 meteorological conditions are representative of the burn’s actual weather conditions, then the majority of burns will have been conducted within or close to these prescription windows, since published guidelines represent the optimal window and are sometimes legally required conditions for burning.

**Fig. 23:**
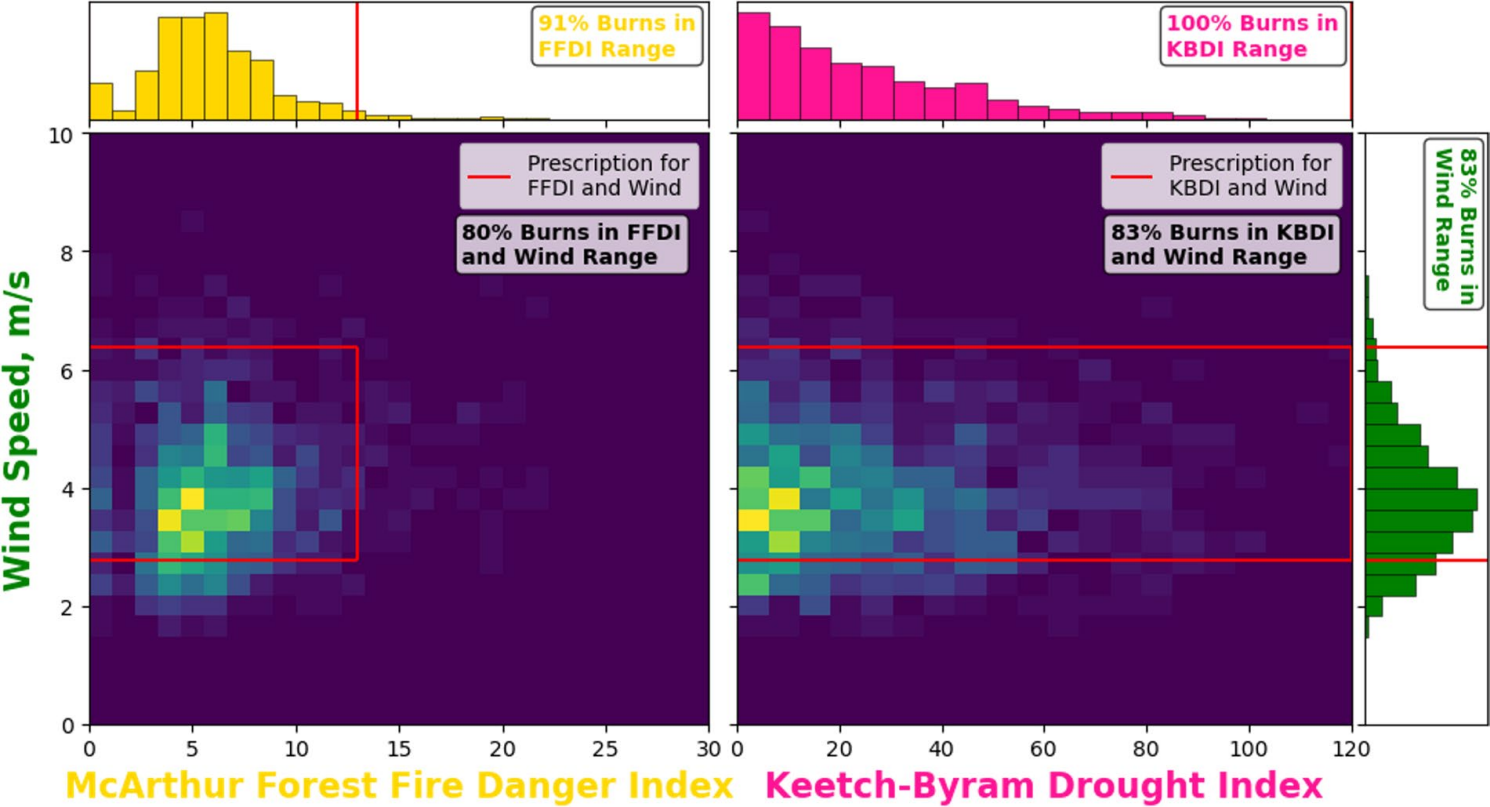
1D and 2D histograms of RxB records from the South Brigalow Belt bioregion under prescription guideline meteorological variables (2-metre wind speed, MacArthur Forest Fire Index (FFDI), and Keetch-Byram Drought Index (KBDI)). 2D histograms indicate the distribution of burns falling under 2-metre wind speed prescription and other meteorological variables’ prescriptions. Prescription guidelines for each variable indicated by red lines and boxes in 1D and 2D histograms, respectively. Histograms set to 20 bins.

We selected prescription windows within regions of Australia, the United States, Portugal, and Sweden, described in Table 4. The majority of prescriptions are based on values of temperature (T), relative humidity (RH), and wind speed (WS), as these variables are particularly influential on fire behaviour^229^. However, several fire weather indices, such as the MacArthur Forest Fire Index (FFDI) and various sub-components of the Canadian Fire Weather Index such as the duff moisture code (DMC) are also used to set prescriptions. We compare these prescription guidelines with values of daily maximum 2m temperature (T_max_), daily minimum relative humidity (RH_min_), daily maximum 10m or 2m wind speed (WS_max_) (converted as appropriate depending on the prescription), daily mean FFDI (FFDI_mean_), drought moisture code (DMC_mean_), fine fuel moisture code (FFMC_mean_), and Keetch-Byram Drought Index (KBDI_mean_) geolocated from the ERA5 dataset, using select variables where they are used in the prescriptions.

**Table 4:** Burn prescription guidelines for select regions.

Prescription Region	T (°C)	RH (%)	WS (m/s)	FFDI (unitless)	DMC (unitless)	KBDI (unitless)	FFMC (unitless)	Source
Australia, Brigalow Belt South	—	—	<6.4* (2.1–5.0)*	**0–13**	—	**<120** (60–90)	—	ref. ^230^
Portugal, shrubland	**(8–20)**	**(20–70)**	**(1.4–4.2)**	—	—	—	—	ref. ^232^
Sweden, boreal forest	—	**32–48**	**0.7–4.8**	—	**28–60**	—	**80–90**	refs. ^237,238,248^

**Fig. 24:**
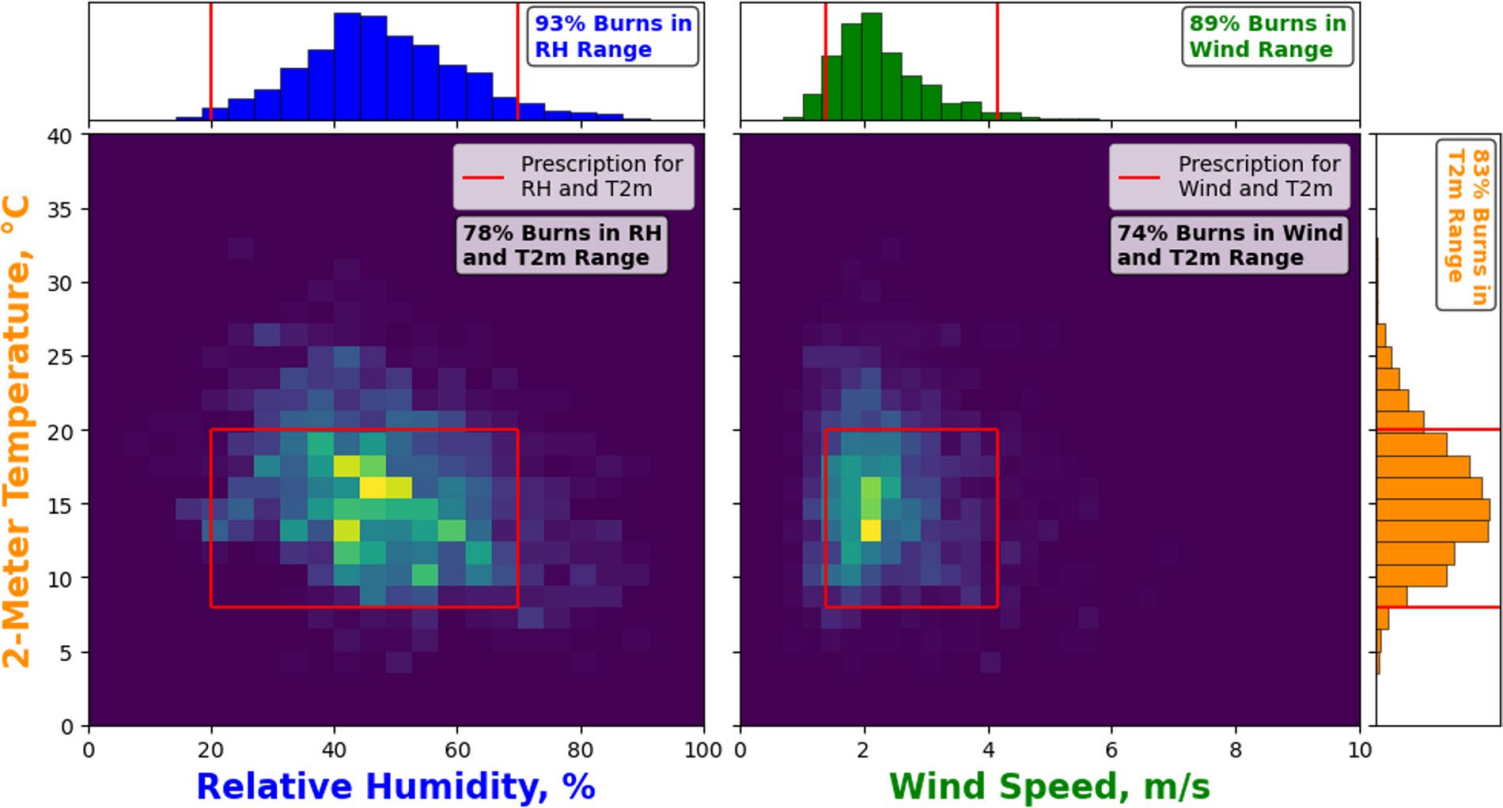
1D and 2D histograms of RxB records from the northern Portugal shrublands under prescription guideline meteorological variables (2-metre temperature, relative humidity, 2-metre wind speed). 2D histograms indicate the distribution of burns falling under 2-metre temperature prescription and other meteorological variables’ prescriptions. Prescription guidelines for each variable indicated by red lines and boxes in 1D and 2D histograms, respectively. Histograms set to 20 bins.

**Fig. 25:**
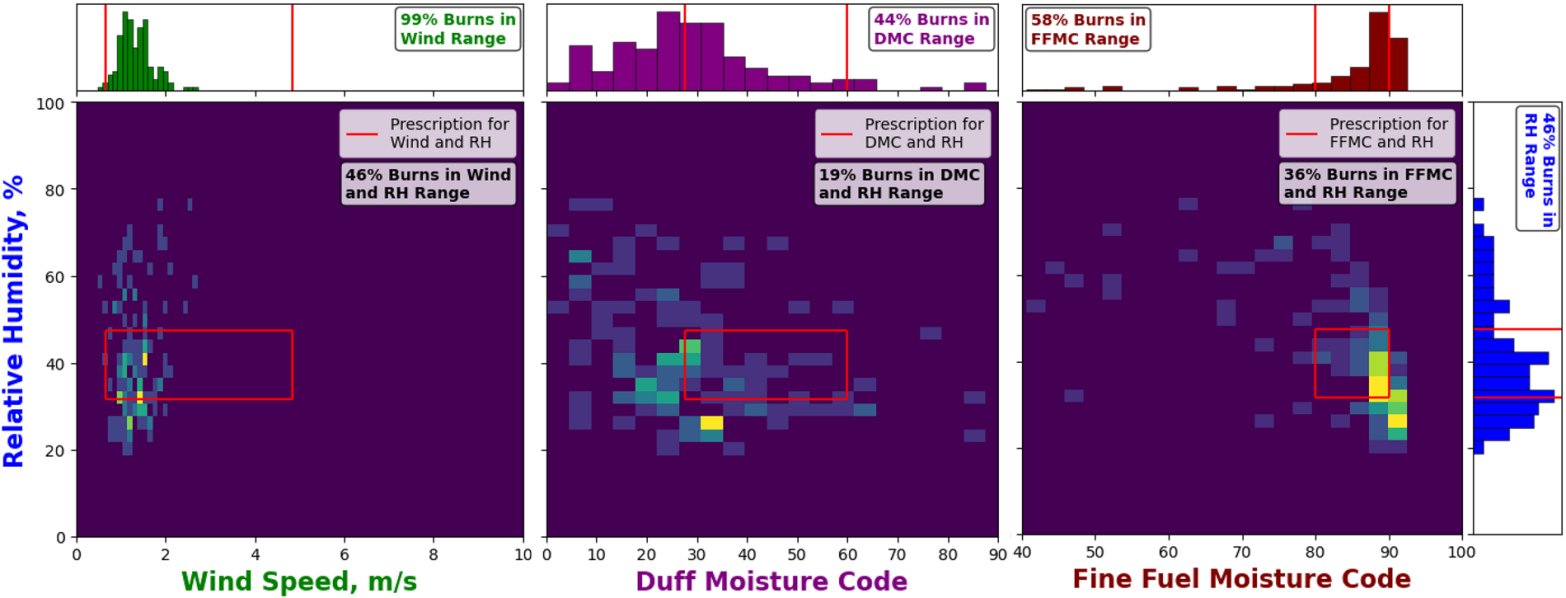
1D and 2D histograms of RxB records in Sweden boreal forests under prescription guideline meteorological variables (RH, 2-metre WS, DMC, and FFMC). 2D histograms indicate the distribution of burns falling under RH prescription and other meteorological variables’ prescriptions. Prescription guidelines for each variable indicated by red lines and boxes in 1D and 2D histograms, respectively. Histograms set to 20 bins.

Optimal ranges are included in parentheses. Bolded values are used in Figs. 23–25.

*10-metre wind speeds.

We selected regions based on a combination of the availability of published RxB weather guidelines and whether sufficient burn records (>100 records) exist for each of these regions. Where possible, we sought regions for which there existed burning prescriptions with high specificity to the vegetation in that region - for example, prescriptions with at least ecoregion level vegetation specificity. This was done to ensure that the chosen prescriptions were the most relevant and therefore the most likely to have been applied to the subsetted records. We aimed to select regions representative of different ecosystem types, including forests, shrublands, and grasslands, as well as burning prescriptions utilising a range of meteorological metrics. Table 4 shows a summary of the regions chosen for analysis with their prescriptions.

For each region, we calculated the percentage of burns falling within each individual variable’s prescription, as well as the percentages of burns falling within a combination of the variables’ prescriptions (e.g., percent of burns within both T and WS prescriptions). Where possible, we compare values and trends with literature and discuss possible reasons for any inconsistencies between the prescriptions and burns’ meteorological values. The ranges of the meteorological values for each case study region is shown in Table 5.

**Table 5:** Range of RxB meteorological values for each case study region.

Prescription Region	T_max_ (°C)	RH_min_ (%)	WS_max_ (m/s)	FFDI (unitless)	DMC (unitless)	KBDI (unitless)	FFMC (unitless)
Australia, Brigalow Belt South	—	—	1.4–8.7* (2.6–5.3)*	0–22 2–10	—	0.1–122 3 - 55	—
Portugal, shrubland	4–33 (10–21)	6–91 (10–21)	0.7–7.1 (1–3)	—	—	—	—
Sweden, boreal forest	—	19–78 (26 - 62)	0.6–3.3 (1.1–2.3)	—	0.3–88 (9– 49)	—	41–92 (73–91)

10^th^ to 90^th^ percentile ranges of meteorological values shown in parentheses.

*10-metre wind speeds.

In general, the majority of the burns’ ERA5 meteorological values fall within the prescription guidelines in all of the selected regions except for Sweden, and decrease in overlap as the recommended burning season ends. Of the regions analysed, the Brigalow Belt South had the highest proportion of burns (79%) falling within acceptable prescription guidelines for all meteorological variables, followed by Portugal shrublands (70%), and then Sweden boreal forest (14%). High overlap between the prescriptions and the burns’ meteorology support the use of ERA5 meteorological values for capturing the general weather conditions on the date of the burns, despite the relatively coarse resolution of the ERA5 data compared to the relatively local scale of the burns. For regions where the overlap between prescriptions and burns’ meteorology is low, we recommend supplementing the ERA5 data provided in GlobalRx with regional meteorological datasets or observations. More detail on each region can be found in the sections below. An additional case study for the Sierra Nevada region in California can be found in the Supplementary Text S1.

### Australia - Queensland, Brigalow Belt

The Brigalow Belt runs between the tropical rainforest of the northern coast and northern New South Wales and comprises two Australian bioregions, the Brigalow Belt North (BBN) and Brigalow Belt South (BBS). It is primarily composed of acacia-wooded grasslands, with Dichanthium grasslands in the north and eucalyptus woodlands towards the south. The eucalypt forest and woodlands comprise the largest regional ecosystem within the bioregion^230^. For our analysis, we focus on the Brigalow Belt South as there were more data points than BBN.

Prescription ranges were taken from guidelines published by the Queensland Parks and Wildlife Service (QPWS) Enhanced Fire Management Team for different ecosystems in the Brigalow Belt bioregion. The chosen prescription guidelines are applicable to Eucalypt forest and woodlands for the objective of maintaining healthy shrubby eucalypt forests and woodlands. The recommended season for burning is austral autumn to early spring, and burning at different times of the year is also recommended to maximise species diversity. Fire severity of these burns is generally low, but occasionally moderate severity will be used to control overabundant trees^230^. The prescription has specifications for only WS, FFDI, and KBDI. We included all burns conducted in the Brigalow Belt South, as defined by the Interim Biogeographic Regionalisation for Australia (IBRA).

79% of all burns in this region fell within all the prescriptions. The percentage of burns falling into each prescription parameter is shown in Fig. 23. Nearly all burns fall within the acceptable recommended KBDI guidelines, with >99% of burns having values <120. Only 8% of burns fall within ideal recommended KBDI conditions between 60–90; however, the majority of burns are conducted at far lower KBDI values, with 84% of burns conducted under a value of 50, corresponding to conditions where the soil and large class fuel moistures are high and do not contribute much to fire intensity, typically during the spring dormant season following winter precipitation^52^. This is consistent with the recommendation to conduct burns under wetter soil moisture conditions to ensure the preservation of a range of ecosystem features, such as retaining a grass base and minimising the loss of habitat features and erosion^230^. Temporally, the highest proportion of burns falling outside of WS and FFDI prescriptions each month also occur close to or outside of the recommended burning season (April to September), indicating that the ERA5 data to some extent captures the temporal variation in burn weather conditions described in the guidelines.

### Portugal, shrubland

Portugal is dominated by forest woodlands and shrublands in the north and evergreen oak woodlands in the south. The majority of RxBs in Portugal are conducted in the shrublands in the north, predominantly in for hazardous fuel reduction, as this vegetation tends to be more flammable^124,231^. Our analysis focuses on the shrublands in the north, as the majority of data fall into this region.

Prescription ranges were taken from the general guidelines for RxB in shrublands in Portugal, published in the Handbook to Plan and Use Prescribed Burning in Europe^232^. The prescription guidelines are applicable to a range of shrubland vegetation types, including atlantic, sub-atlantic and Mediterranean shrublands, as well as areas co-dominant with Kermes oak (*Quercus coccifera*)^232^. We included all burns conducted above a latitude of 40°N in order to select for burns in shrublands. We did not select for burns using the Pettinari and Chuvieco land cover, biome, or ecoregion, as these classifications did not accurately capture shrubland extent as described in the RxB literature^124^. WS_max_ values were multiplied by a factor of 0.67 to convert from 10m to 2m in shrublands^233^.

70% of all burns in this region fell within all the prescriptions and overall, there is a high degree of overlap between the burn weather conditions and the prescription ranges, and burns concentrated in the center-most ranges of all meteorological prescriptions (shown in Fig. 24). T_max_ and WS_max_ values match RxB distributions extracted from field forms described in ref. ^234^. The distribution of RH_min_ values from ERA5 are skewed to lower values compared to the prescription distributions in ref. ^234^, but daily mean RH values in GlobalRx are consistent with these distributions. Burns fell outside of the prescription range most frequently due WS_max_ conditions below the prescription recommendation (8% of burns) and above T_max_ conditions (14%). The first finding is also consistent with ref. ^234^, which identified a similar proportion of burns falling out of prescription due to low WS. Our analysis identifies a higher proportion of burns falling out of prescription due to high T. This may be due to the later period covered in GlobalRx (2005–2023) compared to ref. ^234^; 1979–2011), as Portugal has undergone warming in recent decades^235,236^, though further analysis would be needed to confirm this attribution. Nonetheless, the ERA5 values are generally consistent with the patterns from measurements in ref. ^234^.

### Sweden, boreal forest

Sweden is dominated by boreal forest and taiga, and to a lesser extent temperate broadleaf mixed forests. Forests targeted in prescribed burns are typically dominated by Scots pine (*Pinus sylvestris)*, Norway spruce (*Picea abies*), and aspen (*Populus tremuloides*), with the objective of restoring and conserving fire-dependent tree, bird, and insect species. This is sometimes achieved by inducing spruce mortality to open the canopy and favour either broadleaves or pines^187^.

Prescription ranges were calculated by taking the average of different ranges found in presentations and reports produced within the Life Taiga Project^237–240^. The primary objective for these prescription guidelines is conservation and restoration, primarily through changing the forest composition towards a more broadleaf- or pine-dominated structure by inducing spruce mortality. Burns are generally of low intensity, but higher intensity burns may be used to create bare soil for broadleaf restoration^187^. We included all burns conducted in Sweden for our analysis, as all records originate from the Life Taiga project. WS values were multiplied by 0.33 to adjust from 10m to 2m wind speed in relatively open forest stands^241^.

Only 14% of all burns fell within all the prescriptions for this region, with 46%, 99%, 45%, and 58% of burns falling within the RH, WS, DMC, and FFMC prescriptions, respectively (shown in Figure 25). Only WS_max_ values overlap significantly with WS prescription, and are similar to WS measurements made during RxBs in refs. ^238–240^, between 0.4–4 m/s. 28% and 25% of all the burns fall below the RH and above the FFMC prescriptions, respectively, corresponding to conditions drier than the prescription ranges, and 26% and 17% burns fall above the RH and below the FFMC prescriptions, respectively, corresponding to conditions wetter than the prescriptions ranges.

It is possible that the inconsistency between the ERA5 data and the prescription range is because the chosen prescriptions were not generalizable to all burns. It is also possible that the actual meteorological conditions under which the burns were conducted are not accurately captured by the ERA5 data. For example, there is some evidence that ERA5 2-metre temperature is overestimated in Scandinavian regions, particularly Sweden^242^. This could partially explain the burns falling below the RH prescription (and subsequently above DMC and FFMC prescriptions). Similarly, precipitation in this region has been shown to be overestimated^243^, providing a possible explanation for burns falling above DMC and FFMC prescriptions. Regardless, we acknowledge that further scrutiny of the ERA5 meteorology in Sweden, perhaps with more regional data or measurements, is needed to confirm whether it is representative of burning conditions, and we recommend the ERA5 data provided in GlobalRx be used with these considerations in mind.

## Usage Notes

We encourage the use of GlobalRx for further research on RxB use and trends, with consideration to its limitations in spatial coverage and meteorological accuracy. For regional analyses that require a high level of accuracy or resolution of meteorological conditions, we recommend supplementing the ERA5 data provided in GlobalRx with regional meteorological datasets or observations.

It should also be noted that some records within GlobalRx, especially those that were filtered from public fire records, may contain errors propagated from the original records. For example, a very small fraction (<0.1%) of records marked as prescribed fires contain large burned areas (e.g., records labelled as controlled burns in Australia with burned areas in excess of 100,000 ha), and it is possible that either the fire type or the burned area was mislabeled in the original record. We have retained these records within the dataset because choosing an appropriate threshold for removing records would involve making arbitrary choices that are challenging to validate. Nonetheless, we advise users to carefully inspect and consider filtering these records as required for their specific application.

We emphasise that GlobalRx is also only a subset of all burns conducted with governmental notice or approval. Where data are not nationally monitored and centralised, data reporting can vary significantly, thus affecting the overall data coverage. Additionally, prescribed burns are often planned, managed, and monitored at a regional or sub-regional scale, and thus many burns are only recorded at this scale. While GlobalRx contains regional records from several countries, our data acquisition was also limited by access to and labour intensity of scraping individual regional and sub-regional databases. While GlobalRx is by no means a complete global record of prescribed fires, it is the most comprehensive global record to our knowledge. In this regard, we seek additional data for future versions of GlobalRx and welcome contributions from any additional providers, especially from underrepresented regions. We also note that GlobalRx does not include information about the success of burns with respect to objectives set out in burn plans because no underlying dataset provided such information. However, we welcome records of this kind and they will be incorporated if they become available in future. Please get in touch with the corresponding author if you are interested in contributing data.

We have defined RxB to be a form of controlled burning that is conducted under published regional, state, federal governmental, or other institutional approval and prescription standards which are defined in terms of scientific metrics, such as meteorological quantities and fire weather indices. All data in GlobalRx were acquired through contacts who have either governmental, educational, or other institutional affiliations. Thus, GlobalRx only includes burns for which there exists an institutional record. As such, GlobalRx does not include other forms of controlled fire use, which may apply prescriptions based on any combination of experiential, generational, Indigenous or traditional ecological knowledge, and scientific knowledge^244,245^ but for which no documented institutional records exist. Lastly, we acknowledge that the extent of records in GlobalRx may not necessarily reflect the prevalence of fire management programs or RxB in a country or region, due to the limitations described above as well as limited access to existing data and databases.

## Supplementary information

Supplementary Information for A global assemblage of regional prescribed burn records — GlobalRx

## Data Availability

All code used to add global layers to GlobalRx (meteorology, ecological features) and produce all figures are archived in our Zenodo repository (https://zenodo.org/records/13379463). Code used to preprocess global layers, as well as preprocessed global layer datafiles, are also included, where relevant.
