# Supplementary material for: A global assemblage of regional prescribed burn records — GlobalRx
Source: Sci Data. 2025 Jul 1;12:1083. doi: 10.1038/s41597-025-04941-w (PMC12217793; doi:10.1038/s41597-025-04941-w)
Supplement: Supplementary file 1 — Supplementary Information for A global assemblage of regional prescribed burn records — GlobalRx [file 41597_2025_4941_MOESM1_ESM.docx]

Supplementary Material

# Supplementary Tables

**Supplementary Table 1:** Datasets assembled in GlobalRx (version 2024.1). Public datasets were available via open access links (see reference column). Private datasets were made available upon request.

| **Country** | **State/Region** | **Agency/Organisation** | **Dataset Name** | **Access** | **Format** | **# Records** | **Years included** | **Reference** |
| --- | --- | --- | --- | --- | --- | --- | --- | --- |
| Australia | New South Wales | New South Wales Department of Planning, Industry and Environment (NSW-NPWS) | Fire History - Wildfires and Prescribed Burns | Public | Shapefile (point) | 7051 | 1979-2023 | ([NSW-NPWS, 2024](https://datasets.seed.nsw.gov.au/dataset/fire-history-wildfires-and-prescribed-burns-1e8b6))[^1^](https://www.zotero.org/google-docs/?NXZICe) |
| Australia | Northern Territory | Darwin Centre of Bushfire Research | North Australia and Rangelands Fire Information (NAFI) | Private | Shapefile (line) | 344 | 2013-2021 | (NAFI, 2021)[^2^](https://www.zotero.org/google-docs/?U9HHpl) |
| Australia | Queensland | Queensland Parks and Wildlife Service (QPWS) | Fire History | Public | Shapefile (point) | 8302 | 1979-2023 | ([QPWS. 2024](https://www.data.qld.gov.au/dataset/fire-history-queensland-parks-and-wildlife-service))[^3^](https://www.zotero.org/google-docs/?jEKsZV) |
| Australia | South Australia | Government of South Australia Department for Environment and Water (SA-DEW) | Fire History | Public | Shapefile (point) | 1377 | 1979-2023 | ([SA-DEW, 2024](https://data.sa.gov.au/data/dataset/fire-history))[^4^](https://www.zotero.org/google-docs/?1luxvX) |
| Australia | Tasmania | Tasmanian Department of Primary Industries, Parks, Water and Environment (TAS-DPIPWE) | ListData - Fire History | Public | Shapefile (point) | 2067 | 1979-2023 | ([TAS-DPIPWE, 2024](http://listdata.thelist.tas.gov.au/opendata/))[^5^](https://www.zotero.org/google-docs/?DtroeP) |
| Australia | Victoria | Victoria Department of Environment, Land, Water and Planning (VIC-DELWP) | Fire History Records of Fires primarily on Public Land | Public | Shapefile (point) | 89576 | 1979-2023 | ([VIC-DELWP, 2021](https://discover.data.vic.gov.au/dataset/fire-history-records-of-fires-across-victoria))[^6^](https://www.zotero.org/google-docs/?avUATa) |
| Australia | Western Australia | Government of Western Australia Department of Biodiversity, Conservation and Attractions (WA-DBCA) | Fire History (DBCA-060) | Public | Shapefile (point) | 11989 | 1979-2023 | ([WA-DBCA, 2024](https://catalogue.data.wa.gov.au/dataset/dbca-fire-history))[^7^](https://www.zotero.org/google-docs/?LeJais) |
| Brazil | Roraima, Amazonas, Mato Grosso, Mato Grosso do Sul, Goías, Tocantins & Maranhão | IBAMA (Brazilian Institute of the Environment and Renewable Natural Resources) / National Center to Prevent and Combat Forest Fires (PREVFOGO) | Record of prescribed burns on public land | Private | Spreadsheet | 9878 | 2015-2020 | (IBAMA/ PREVFOGO, 2021)[^8^](https://www.zotero.org/google-docs/?cj45IW) |
| Canada | Alberta | Banff National Park | Prescribed burning database | Private | Spreadsheet | 79 | 1983-2019 | (Parks Canada, 2020)[^9^](https://www.zotero.org/google-docs/?2qJx2B) |
| Canada | National | Government of Canada, Natural Resources Canada (NRC) | CWFIS Datamart- Fire History Data | Public | Spreadsheet | 130 | 1993-2021 | ([NRC, 2022](https://cwfis.cfs.nrcan.gc.ca/datamart))[^10^](https://www.zotero.org/google-docs/?AUB8FW) |
| Canada | National | Parks Canada | Record of prescribed burns on national park land | Private | Spreadsheet | 348 | 1985-2019 | (Parks Canada, 2020)[^11^](https://www.zotero.org/google-docs/?GkJmh7) |
| France | East-Pyrenees | National Research Institute for Agriculture, Food, and Environment (INRAE) / East-Pyrenees Prescribed Burn Team | Record of prescribed burns on public and private ands of the East-Pyrenees Prescribed Burning Team | Private | Spreadsheet | 1448 | 1984-2016 | (INRAE, 2021)[^12^](https://www.zotero.org/google-docs/?aM5KK9) |
| Germany | Brandenburg | German Federal Real Estate Administration | Record of prescribed burns on public land | Private | Spreadsheet | 3 | 2019 | (German Federal Real Estate Administration, 2021)[^13^](https://www.zotero.org/google-docs/?T7OnaR) |
| Italy | Piedmont | Italian Society of Silviculture and Forest Ecology | Record of prescribed burns on public land | Private | Spreadsheet | 135 | 2005-2021 | (Italian Society of Silviculture and Forest Ecology, Fire Management, 2021)[^14^](https://www.zotero.org/google-docs/?S3qVmT) |
| Japan | National | Hokkaido University | Record of prescribed burns on public land from regional authorities | Private | Spreadsheet | 407 | 1973-2021 | (Yamashita, Y. / Hokkaido University, 2021)[^15^](https://www.zotero.org/google-docs/?AyQz9m) |
| Mexico | National | National Forestry Commission (CONAFOR) | Records from the federal prescribed burn program | Private | Spreadsheet | 20 | 2017-2021 | (CONAFOR, 2021)[^16^](https://www.zotero.org/google-docs/?wvVQQA) |
| Portugal | National | Institute for Nature Conservation and Forests (ICNF) | Record of prescribed burns on public and private land | Private | Spreadsheet | 2841 | 2002-2022 | (ICNF, 2023)[^17^](https://www.zotero.org/google-docs/?KYvHkF) |
| Russia | National | Wildfires Monitoring Information System of the Federal Forestry Agency (ISDM-Rosleskhoz) | Record of prescribed burns in Russia | Private | Spreadsheet | 22142 | 2008-2020 | (ISDM-Rosleskhoz, 2021)[^18^](https://www.zotero.org/google-docs/?Sg9slz) |
| South Africa | Kruger National Park | South Africa National Parks (SANParks)* | Record of prescribed burns in Kruger National Park | Private | Shapefile (polygon) | 992 | 1979-2020 | (SANParks, 2021)[^19^](https://www.zotero.org/google-docs/?a9MnDm) |
| South Africa | Garden Route National Park | South Africa National Parks (SANParks)* | Record of prescribed burns in Garden Route National Park | Private | Shapefile (polygon) | 28 | 1984-2021 | (SANParks, 2021)[^20^](https://www.zotero.org/google-docs/?HQdoOK) |
| South Africa | Table Mountain National Park | South Africa National Parks (SANParks)* | Record of prescribed burns in Table Mountain National Park | Private | Shapefile (polygon) | 45 | 1975-2019 | (SANParks, 2021)[^21^](https://www.zotero.org/google-docs/?GMi1Ac) |
| Spain | Andalucía | Regional Government of Andalucia | Record of prescribed burns on public land | Private | Spreadsheet | 79 | 2013-2021 | (Government of Andalucia, 2021)[^22^](https://www.zotero.org/google-docs/?0hNTny) |
| Spain | Asturias | Regional Government of Asturias | Record of prescribed burns on public land | Private | Spreadsheet | 254 | 2006-2021 | (Principality of Asturias, 2021)[^23^](https://www.zotero.org/google-docs/?ImUNaG) |
| Spain | Galicia | Regional Government of Galicia | Record of prescribed burns on public land | Private | Spreadsheet | 200 | 2018-2021 | (Government of Galicia, 2021)[^24^](https://www.zotero.org/google-docs/?M6qg78) |
| Spain | Catalonia | Pau Costa Foundation / Government of Catalonia (GenCat) Department of the Interior | Prescribed burning by firefighters of the Generalitat de Catalunya since 1998 | Public | Shapefile (point) | 518 | 1998-2017 | ([GenCat, 2021](https://interior.gencat.cat/ca/serveis/informacio-geografica/bases-cartografiques/cremes-prescrites-dels-bombers/))[^25^](https://www.zotero.org/google-docs/?DKfd0L) |
| Sweden | National | LifeTaiga Project (EU H2020) | Record of prescribed burns during the LifeTaiga Science Project (EU Commission) | Private | Spreadsheet | 134 | 2015-2020 | (LifeTaiga Project., 2021)[^26^](https://www.zotero.org/google-docs/?WtPBle) |
| Thailand | National | Thailand Ministry of Natural Resources and Environment, Department of National Parks, Wildlife and Plants Conservation (Forest Fire Control Division) | Record of prescribed burns on national park land | Private | Spreadsheet | 174 | 2022 | (Thailand Ministry of Natural Resources and Environment, Department of National Parks, Wildlife and Plants Conservation (Forest Fire Control Division), 2022)[^27^](https://www.zotero.org/google-docs/?jUF3S0) |
| UK | Hampshire | Forestry England | Record of prescribed burns in New Forest National Park | Private | Spreadsheet | 1644 | 1996-2020 | (Forestry England, 2021)[^28^](https://www.zotero.org/google-docs/?IsJjjK) |
| USA | National | National Interagency Fire Center (U.S. Geological Survey, USDA Forest Service) | Monitoring Trends in Burn Severity from 1984-2021 | Public | Shapefile (polygon) | 1979 | 1984-2023 | ([NIFC 2021](https://www.mtbs.gov/))[^29^](https://www.zotero.org/google-docs/?JWwx3X) |
| USA | National | US Department of Agriculture Forest Service | Fire and Tree Mortality database (FTM) | Public | Spreadsheet | 79 | 1981-2016 | ([Cansler et al., 2020](https://www.fs.usda.gov/rds/archive/catalog/RDS-2020-0001))[^30^](https://www.zotero.org/google-docs/?mNjFFQ) |
| USA | National | US Department of Interior | The National Fire Plan Operations and Reporting System (NFPORS) Hazardous Fuels Reduction (HFR) Module | Public | Shapefile (point) | 9132 | 2000-2021 | ([US Department of Interior 2021](https://iftdss.firenet.gov/))[^31^](https://www.zotero.org/google-docs/?Dj5xq7) |
| USA | National | US Geological Survey | Combined wildland fire datasets for the United States and certain territories, 1800s-Present: U.S. Geological Survey data release | Public | Shapefile (polygon) | 31136 | 1979-2020 | ([Welty and Jeffries 2021](https://www.usgs.gov/data/combined-wildland-fire-datasets-united-states-and-certain-territories-1800s-present))[^32^](https://www.zotero.org/google-docs/?GCMphg) |
| *data release under SANParks project number SS375. | | | | | | | | |

#

# Supplementary Case Study Regions

**Supplementary Table 2**: Burn prescription guidelines for case study region. Optimal ranges are included in parentheses. Bolded values are used in Figures 23-26.

| **Prescription Region** | **T (°C)** | **RH (%)** | **WS (m/s)** | **FFDI (unitless)** | **DMC (unitless)** | **KBDI (unitless)** | **FFMC (unitless)** | **Source** |
| --- | --- | --- | --- | --- | --- | --- | --- | --- |
| Sierra Nevada Forests | **3-30**  (18-21) | **18-68**  (20-30) | **1-7***  (3-4)* | — | — | — | — | ref. ([^33^](https://www.zotero.org/google-docs/?vqWIYE)) |

*10-metre wind speeds

**Supplementary Table 3**: Range of RxB meteorological values for case study region. 10^th^ to 90^th^ percentile ranges of meteorological values shown in parentheses.

| **Prescription Region** | **T_max_ (°C)** | **RH_min_ (%)** | **WS_max_ (m/s)** | **FFDI (unitless)** | **DMC (unitless)** | **KBDI (unitless)** | **FFMC (unitless)** |
| --- | --- | --- | --- | --- | --- | --- | --- |
| Sierra Nevada Forests | -6 - 36  (8 - 27) | 4 - 88  (14 - 50) | 0.9 - 11*  (2 - 5)* | — | — | — | — |

*10-metre wind speeds

### **United States - California, Sierra Nevada Forests**

The Sierra Nevada ecoregion covers approximately 53,000 km² in predominantly California (98%) and Nevada. The ecoregion has a predominantly Mediterranean climate, with alpine climates at higher elevations[^34^](https://www.zotero.org/google-docs/?oXBSo7). Vegetation varies with topography and elevation, with mixed forests of oaks and coniferous species at lower elevations, and higher proportions of coniferous species at higher elevations such as Douglas fir (*Pseudotsuga menziesii*), ponderosa, Jeffrey, and lodgepole pines (*Pinus ponderosa*, *Pinus jeffreyi,* and *P. contorta*), red and white fir (*Abies concolor*, *Abies magnifica*)[^35^](https://www.zotero.org/google-docs/?oLXQV4). These biotic zones are fire-adapted, with high frequency, low severity fire at lower elevations that decrease in frequency and increase in severity with elevation. RxBs in these zones are most commonly conducted at low severity to clear understory fuels and rejuvenate forbs, especially in lower zones dominated by Ponderosa and Jeffrey pine.

Prescription ranges for this region were calculated from RxB plans collected from RxBs provided by ref ([^33^](https://www.zotero.org/google-docs/?BaCWT9)). We selected the burn plans in the Sierra Nevada range, totaling four plans for burns in the summer, fall, and winter. We then averaged the acceptable and optimal T, RH, and WS ranges across these plans, resulting in the ranges shown in **Table 5**. Burn records for this ecoregion were filtered by extracting all burns occurring within the Sierra Nevada forests ecoregion, as defined by the Olson ecoregion, and which had an elevation value above 900m, as defined by the GMTED2010 global digital elevation model. This yielded 1,594 records.

74% of all burns in this region fell within all the prescriptions. The percentage of burns falling into the WS, T, and RH prescriptions is shown in **Supplementary Figure 1**. The WS_max_ and T_max_ distributions are centered within the prescription ranges, indicating that ERA5 meteorology for these parameters is generally representative of actual prescription ranges. The majority of RH_min_ values are within the prescription range but skewed towards lower values. While it is possible that burns may have been conducted outside RH prescription (e.g., ref ([^36^](https://www.zotero.org/google-docs/?XIik6d)) found RH to be the most limiting parameter compared to T and WS in California RxBs) given the legal requirements of burn plans, we assume that this would only affect a very small portion of burns[^37^](https://www.zotero.org/google-docs/?ZFNIKX). It is also possible that these RxBs were not conducted during the time of day during which the RH_min_ occurred. **Supplementary Figure 2** supports this explanation, since no daily mean RH values for the burns fall below the RH prescription. Hence, nearly all burns fall within prescription if it is assumed that the burns were conducted under conditions between the daily minimum and mean RH values.


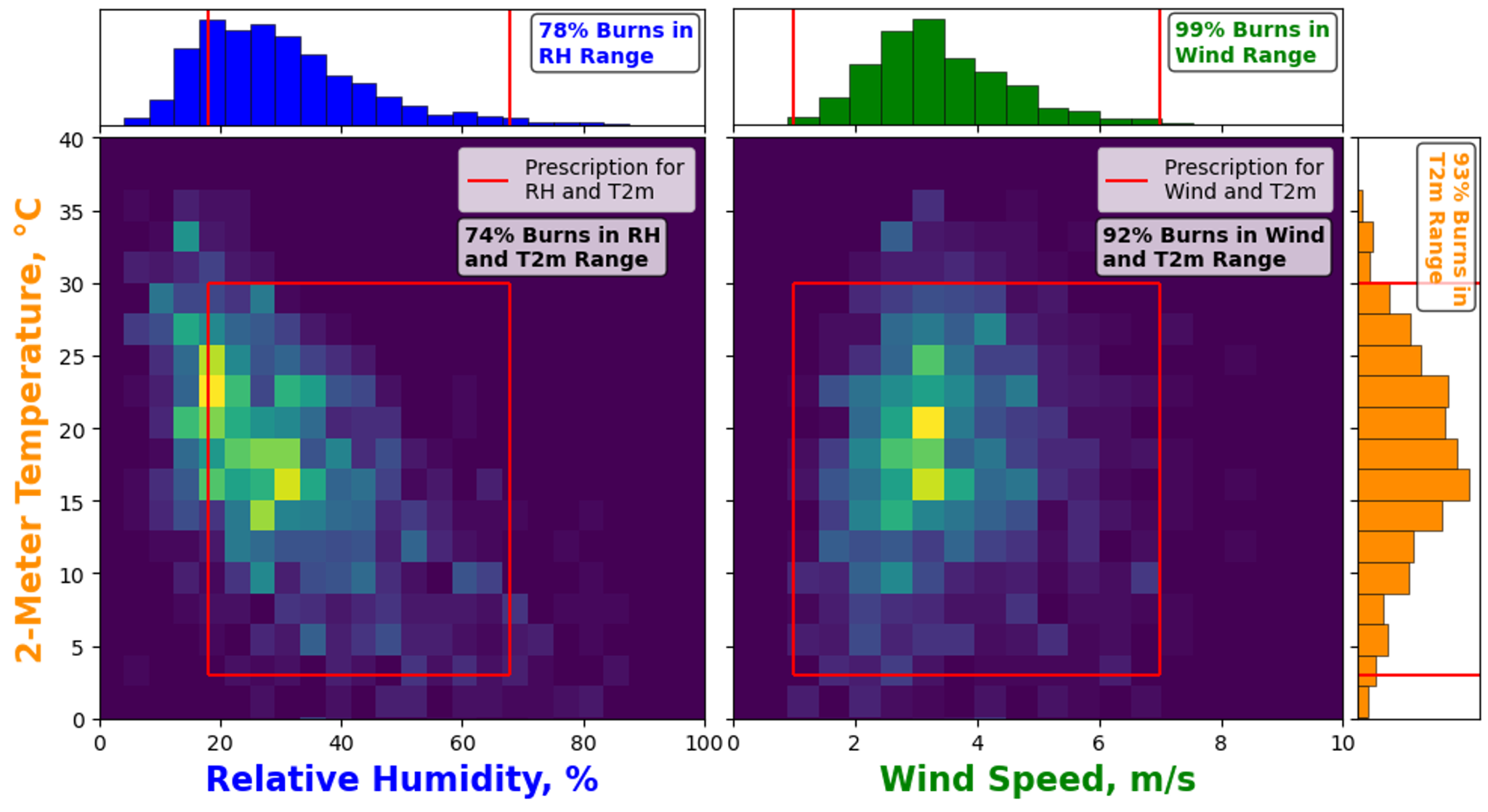


**Supplementary Figure 1**: 1D and 2D histograms of RxB records from the Sierra Nevada forests under prescription guideline meteorological variables. 2D histograms indicate the distribution of burns falling under 2-metre T prescription and other meteorological variables’ prescriptions. Prescription guidelines for each variable indicated by red lines and boxes in 1D and 2D histograms, respectively. Histograms set to 20 bins.


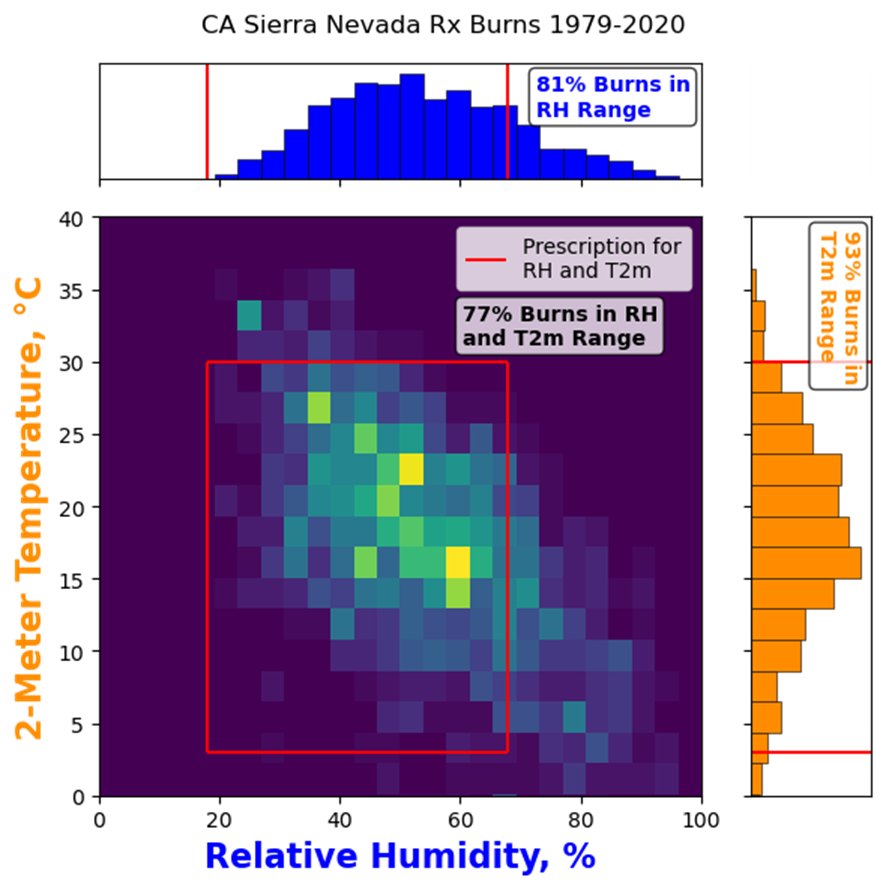


**Supplementary Figure 2**: Values of daily mean relative humidity and daily maximum 2m temperature for the Sierra Nevada Forests ecoregion.

# Extended Historical Context and Analysis for Prescribed Burning in GlobalRx Countries

## ***Australia***

The majority of prescribed burns in Australia are conducted in the southeast region (New South Wales, Victoria) of the country, where the country’s highest population densities coincide with some of the most flammable landscapes in the world. Southeast Australia is dominated by wet and dry sclerophyll forests and eucalypt woodlands interspersed with rainforest and other forest types, grasslands, heathlands, and shrublands[^38^](https://www.zotero.org/google-docs/?3B3gC7). The region is one of the most fire-prone areas in the world, owing to a combination of climate that brings in dry, northerly desert winds, steep topography, and the prevalence of extremely flammable eucalypt species, such as Eucalyptus regnans (mountain ash), Eucalyptus obliqua (stringybark), and Eucalyptus delegatensis (alpine ash)[^39^](https://www.zotero.org/google-docs/?ou42Tj). The vegetation in the region possesses numerous traits that make it prone to burning, including flammable, resin-filled foliage, open crown structures, as well as fire adaptations, such as the ability to develop lignotubers, regenerate prolifically from seed, or resprout post-fire. However, some wet sclerophyll eucalypt species, such as Eucalyptus regnans, are obligate seeders, and hence vulnerable to changes to fire return intervals[^40^](https://www.zotero.org/google-docs/?Z34QLY).

These ecosystems have long been managed by Aboriginal people, including but not limited to the Dja Dja Wurrung and Wurundjeri peoples of modern-day Victoria, and the Barkindji, Bundjalung, Dharug, Ngiyampaa, Muthi Muthi, and Wiradjuri peoples of modern-day NSW. These people have occupied the area for more than 50,000 years and burned the landscape to hunt and attract game such as kangaroos, facilitate movement, promote the growth of food staples such as seeds, fruits, and tuberous roots, and out of moral obligation to “cleanse” the land of senescent vegetation[^41–43^](https://www.zotero.org/google-docs/?bywOGa). Burning typically took place in dry sclerophyll forests and grasslands during high to late summer, at an interval of 2-5 years[^43^](https://www.zotero.org/google-docs/?QFm5Ib). It is now widely recognized by the western scientific community that Aboriginal burning created a mosaic of varying post-fire successional stages and burnt and unburnt vegetation that favored both faunal and floral species richness, maintained an open forest structure, and likely evolutionarily selected for communities of species that benefited from their fire regime[^43^](https://www.zotero.org/google-docs/?Vv2Ez9).

European colonization, which began in the 1700s, brought about new land use systems with new grain crops, the introduction of domestic farm animals, and the concept of land ownership and townships. While settlers used fire for agricultural clearing and converting forests, woodlands, and shrublands into pastures and croplands, European settlement generally coincided with a decrease in annual burned area but an increase in damaging and intensive fires in the Southeast, especially as Aboriginal populations were increasingly decimated and displaced by growing settler populations spurred by gold rushes in 1850[^38^](https://www.zotero.org/google-docs/?WAgThA). The thickening of forest growth and increasing wildfire intensity was noted as early as 1890 by Alfred Hewitt, who attributed this change to the decline of Aboriginal influence on the landscape[^38^](https://www.zotero.org/google-docs/?lsUDoR). Beginning in the 1900s, national industrial development and the growing demand for timber by the rapidly expanding settler population led to the establishment of governmental forest management agencies, which formalized European forest and land management in the region.

Initial forest management was based on fire suppression, but the development of the forestry industry in the 1960s led to corresponding developments in forest and fire science. During this time, prescribed burning was developed as a management tool to reduce the risk of wildfire in timber assets, regenerate harvested eucalypt stands, and prepare areas for the establishment of softwood plantations, and government agencies advocated strongly for its use for wildfire mitigation[^38,44^](https://www.zotero.org/google-docs/?T2Xw5E). Increases in funding for prescribed burning research and technological developments, particularly that of airborne incendiary machines that enabled broadscale application of prescribed fire, greatly expanded the prescribed burn area between the 1960s-1980s. However, prescribed burning has come under increasing political scrutiny since the 1980s, brought in part by a new public environmental consciousness and the inherent uncertainty in fire science[^42^](https://www.zotero.org/google-docs/?L4JsMG).

Nevertheless, prescribed burning is still prevalent in the southeast, with over 300,000 ha burnt annually in Victoria and NSW combined for hazardous fuel management and ecological management[^38,45^](https://www.zotero.org/google-docs/?bbo4Pj). In Victoria, prescribed burning on public lands is managed by the Department of Sustainability and Environment (DSE), which sets annual burn targets[^42^](https://www.zotero.org/google-docs/?jxqHm1). Burns are carried out in designated fire management zones, with the interval, objectives, and extent of the burn dependent upon the zone designation. Burn objectives primarily involve reducing wildfire risk and intensity and ecological management[^46,47^](https://www.zotero.org/google-docs/?wuqmjW). In NSW, prescribed burning is common on public lands and on forestry land[^48^](https://www.zotero.org/google-docs/?HXYMl7) for wildfire hazard abatement, as well as nature reserves and national parks for ecological management[^38^](https://www.zotero.org/google-docs/?qADNIM).

South and southwestern Australia is a similarly temperate and fire-prone region dominated by eucalypt forests, with dry sclerophyll forests in the uplands (predominantly jarrah (Eucalyptus marginata) and marri (Corymbia calophylla)) and wet sclerophyll forests (dominated by karri (Eucalyptus diversicolor)) in more fertile regions. A Mediterranean-type climate brings warm, dry summers, and vegetation is dry enough to burn for 6-8 months of the year. This effect is exacerbated by climate change, which has reduced rainfall by at least 20% since the 1970s and extended the fire season to as late as April[^49^](https://www.zotero.org/google-docs/?Z8PYFu). A high proportion of forested lands in this region is publicly owned and is managed for conservation, sustainable timber production, and water catchment protection by Western Australian government agencies. Prior to colonization, the various Indigenous groups, such as the Noongar and Ngadju peoples, used fire typically during dry summer months, maintaining a patch mosaic of different successional stages of vegetation that limited the impact of intense bushfires[^50,51^](https://www.zotero.org/google-docs/?eYK3mZ). Fire exclusion policies introduced in the 1920s later resulted in extensive wildfires, and prescribed fire has been used for fuel management since the 1960s. Hazardous fuel reduction is the most common purpose for prescribed burns, though burning for ecological management, such as for quokka habitat or certain obligate seeder species, is also common. Prescribed burns are conducted using a similar zoning approach as those found in Victoria, with the interval and fire regime determined by the objective for that zone. Prescribed burn targets are upwards of 200,000 ha, and over 90% of this was achieved in 2021-2022[^7,49^](https://www.zotero.org/google-docs/?LcTdeE). However, similar to the southeast, this annual target is becoming increasingly difficult to achieve due to the increasing population size at WUI and the increasing complexity of prescribed burning planning, risk management, and decision making brought on by the politicization of the topic[^49^](https://www.zotero.org/google-docs/?T3eh3M).

In Western Australia, Aboriginal peoples used fire extensively throughout the Western Desert spinifex grasslands to clear senescent vegetation, hunt small animals such as monitor lizards, and maintain various successional stages of vegetation for resource management[^52^](https://www.zotero.org/google-docs/?wyFqcR). This pattern of burning maintained a landscape mosaic of burnt and unburnt patches that prevented large, late dry season fires, prevented the fragmentation and contraction of acacia woodlands, and protected fire-sensitive species[^52,53^](https://www.zotero.org/google-docs/?QkZNNB). Following colonization and the subsequent displacement of Aboriginal people from the Western Desert, populations of native mammals and fire-sensitive plant communities declined substantially, with a third of Western Australian desert mammals becoming endangered or extinct since the 1950s-1970s[^53^](https://www.zotero.org/google-docs/?PCsyh2). Since the 1980s, the Aboriginal Martu people have begun to return to their ancestral homelands and renewed the complex social-ecological systems that mutually benefited the coexistence of humans and non-humans of the Western Desert[^54^](https://www.zotero.org/google-docs/?RAfHrL). Prescribed burning in the region today is managed similarly as other regions of Australia, wherein areas are divided into different management categories with different affected assets[^55^](https://www.zotero.org/google-docs/?G3ZJur). Burns are conducted primarily to manage fuel and bushfire risk and extent, and secondarily for maintaining ecosystem health[^56^](https://www.zotero.org/google-docs/?Vmof9p), informed in part by Aboriginal fire knowledge[^57^](https://www.zotero.org/google-docs/?s17b76).

In the north, the vegetation consists primarily of tropical savannas with eucalyptus woodland, interspersed occasionally with monsoon rainforest and heathlands[^58^](https://www.zotero.org/google-docs/?EijXRX). Aboriginal peoples burned throughout every part of the year, for reasons including hunting and attracting game, pest control, enhancing habitat for important fowl species, protecting valuable plant resources, or enhancing production of important food resources, depending upon the time of the year. Frequent burning created spatiotemporal mosaics of burned and unburned vegetation that maintained high floral and faunal diversity. These burning practices also protected fire-sensitive ecosystems in the region, such as monsoon vine-forests and rainforests [^59^](https://www.zotero.org/google-docs/?OU62Rk). As with other parts of Australia, colonization and the subsequent removal and displacement of indigenous peoples led to an increase in the incidence of late-dry season fires, as well as decreases in species richness. Presently, fire in this region is used for Aboriginal land management and cultural practices, by the pastoral industry, and for conservation management, and prescribed burning is commonly used by managers of all sectors to control late-dry season fires[^60^](https://www.zotero.org/google-docs/?VhZWSB). In addition to hazardous fuel reduction, prescribed burning is conducted widely by and on Aboriginal communities in savannas as part of a carbon sequestration scheme. This carbon reduction program has been implemented since 2006 and aims to contribute to Australia’s Paris Agreement emissions reduction target of 26-28 percent below 2005 levels by 2030. Presently, there are 30 Aboriginal savanna burning projects covering 17.9 million Ha[^61,62^](https://www.zotero.org/google-docs/?HCXoSG).

## Brazil

A significant proportion of prescribed burns occur in the Brazilian Cerrado, the second largest ecoregion in Brazil after the Amazon, which covers 2.1 million km^2^ and is characterized by a gradient of grasslands to savanna woodlands. Like savannas in other regions of the world, recurrent fires have been a key evolutionary force in the ecoregion for millenia: the continuous grass and herbaceous layer, when desiccated during the dry season, easily carries and propagates fire[^63–65^](https://www.zotero.org/google-docs/?u8VDVC). As a result, much of the Cerrado vegetation have developed a number of fire adaptations, including thick, corky bark in trees, resprouting ability, fire-induced flowering, fruiting, and seed production and release[^66–68^](https://www.zotero.org/google-docs/?ukWHcN). In contrast, fire has been a historic but infrequent disturbance in the Amazon; much of its closed-canopy tropical rainforests are fire-sensitive, with even low-severity fire causing significant plant mortality[^69–71^](https://www.zotero.org/google-docs/?dnqDUc), and repeated fire threatening the persistence of the rainforest[^72^](https://www.zotero.org/google-docs/?CF2Xej).

Since 4000 to 5000 yr BP, Indigenous peoples of the Cerrado and Amazon, including the Xavante, Krahô, and Kayapo, have used fire in their daily subsistence to manage food resources and shelter, to hunt, or for rituals and communication[^73,74^](https://www.zotero.org/google-docs/?5SOaI7). Many of these groups independently developed and maintained advanced and incredibly biodiverse agroforestry systems along the forest-savanna border through shifting cultivation, directing forest succession to their needs: fire was used to clear small patches in the forests to create orchards or plant edible roots and bulbs, create fire breaks around these areas, and fertilize the acidic soil (creation of “terra preta”), and specific fire regimes were applied to induce regrowth, flowering, or fruiting of certain species[^68,75^](https://www.zotero.org/google-docs/?wkNTGI). Importantly, the relatively sparse population and mobile lifestyle enabled sufficient fallow periods necessary for the practice to be sustainable[^68,76^](https://www.zotero.org/google-docs/?6DLYxw).

Fire activity in Brazil is closely tied with land use and expansion[^77,78^](https://www.zotero.org/google-docs/?2aDQTC). Cattle ranching was introduced to the country under Portuguese colonization in the 1500s, greatly expanded during the the 1970s during the Brazilian Military Dictatorship, and it remains the dominant land use, as well as the top reason for land clearing in both the Cerrado and the Amazon today; it is estimated that over 70% of areas cleared in both regions are used for livestock grazing[^79–82^](https://www.zotero.org/google-docs/?o3txAE). In the Cerrado, pastoral fires are often set annually or biennially to induce new grass regrowth during the dry season[^68^](https://www.zotero.org/google-docs/?8Qahkz). Fire is also closely tied with land clearing for agriculture: between the 1960s and 1980s, federal policies incentivizing the colonization of the Cerrado ushered in large-scale agriculture based on soybean, sugarcane, eucalyptus, and coffee[^83,84^](https://www.zotero.org/google-docs/?mE267W). Consequently, the highest fire activity occurs within the MAPITOBA region (the southern parts of Maranhão, Tocantins, and Piaui, and western Bahia), the most active part of the agricultural frontier bordering the Cerrado and the Amazon[^83^](https://www.zotero.org/google-docs/?dIsz0i).

The same state-led agrarian policies based on a modernization paradigm drastically intensified settlement in the Amazon[^81^](https://www.zotero.org/google-docs/?siFbOY), while the expansion of infrastructure, such as the Trans-Amazonian Highway, increased access to the forest and reduced transport costs, further escalating deforestation for mining, logging, and large-scale monocropped agriculture[^85,86^](https://www.zotero.org/google-docs/?0glmxj). In the Amazon, land clearing typically proceeds first by logging and timber extraction, followed by regular fires to prevent woody regrowth. The process of logging opens up the canopy, leading to decreased soil moisture and increased fire susceptibility, especially from adjacent pastures[^68,72,85^](https://www.zotero.org/google-docs/?Y3CKi3).

The expanding development frontier has led to environmental degradation as well as the displacement, marginalization, and violence against many Indigenous peoples, as well as other traditional populations and smallholders such as caboclos and quilombolas in the Amazon and Cerrado[^84,87–89^](https://www.zotero.org/google-docs/?TpDSTc). Additionally, much of the environmental legislation enacted to protect the forests, such as the Forest Code and Protected Areas (PAs), are typically based on anti-fire discourse and fire suppression, and neglect to provide fire users with viable alternatives: thus, they are often misaligned with the local reality, customs, and technical capacity, and are consequently ineffective at reducing fire use and activity[^90,91^](https://www.zotero.org/google-docs/?m5v8S6).

Meanwhile, environmental legislation has focused far less on the Cerrado compared to the Amazon: less than 10% of the Cerrado are in PAs, compared to over 25% of the Amazon[^84^](https://www.zotero.org/google-docs/?SDvtVe). Even existing PAs are delimited such that they do not infringe on agricultural expansion areas; thus, the most stringent PAs tend to fall in regions into which traditional populations have been displaced. This has resulted in conflict between traditional populations and conservation policies that severely limit resource use, especially given their anti-fire focus[^84^](https://www.zotero.org/google-docs/?3QuYhC). Furthermore, anti-fire and fire suppression policies have led to fuel accumulation in Cerrado, which threaten the biodiversity, structure, and functioning of the savanna, and has also resulted in an increase in large, human-caused fires in the past 50-60 years, especially in the late dry season (August to October)[^68,83,92^](https://www.zotero.org/google-docs/?XHJiOY).

Through the 1990s and 2000s, research and advocacy led to the increasing recognition that fire suppression policies were ineffective and ecologically detrimental to the Cerrado ecosystem, and in 2014, the conception of the Cerrado-Jalapão Project signified a federal paradigm shift from fire suppression to fire management in PAs and Indigenous territories. The project seeks to create an Integrated Fire Management (IFM) system based on patch mosaic burning, using participative planning involving both scientific and traditional knowledge to reintroduce fire to the Cerrado. It aims to reduce late dry season fires, protect fire-sensitive vegetation, and decrease conflicts between PA and local communities[^93,94^](https://www.zotero.org/google-docs/?4mYcjK). The project is implemented by ICMBio and IBAMA-PREVFOGO, who have jurisdiction in federal protected areas and Indigenous or Quilombola lands, respectively. The first burns were conducted in national parks in Maranhão and Tocantins, and then later in several Indigenous lands and other protected areas[^93,94^](https://www.zotero.org/google-docs/?BbbxNJ). All records in GlobalRx come from IBAMA-PREVFOGO, and are thus predominantly within Indigenous territories.

Brazil’s prescribed burns occur primarily along the so-called Arc of Deforestation - a region encompassing the transition between the Cerrado and Amazon, and which runs through the states of Pará, Mato Grosso, Rondônia, Amazonas, and Acre, and along the western edges of Maranhão and Tocantins, in which deforestation rates are highest[^95,96^](https://www.zotero.org/google-docs/?9y7rhv). 64% (6,187) of burns were conducted in the Cerrado ecoregion, and an additional 17% (1,630) in the Mato Grosso seasonal forests, an ecoregion characteristic of the transition area. The majority of these burns (65%, 4,003 burns; 66%, 1,080 burns) are conducted for some form of hazardous fuel reduction.

1,688 (17%) of burns were conducted in the state of Roraima, in the ecoregions that constitute the Guiana Shield, which spans across northern Brazil, southern Venezuela, Guyana, Suriname, and French Guiana. The region is home to the Pemón, Arekuna, Kamarakoto, and Taurepang Indigenous peoples from Venezuela, and the Makushi and Wapishana Indigenous peoples from Brazil and Guyana. This region consists of both fire-dependent grasslands and savannas, in which the majority of burns (94%, 1,580) were conducted, and fire-sensitive tropical humid gallery forests. In this environment, more than 90% of fires are caused by humans, where it is commonly used by the Indigenous groups for agriculture, hunting, managing edible plants, and controlling savanna fuel loads[^92^](https://www.zotero.org/google-docs/?AQjh52). Here, there are active participatory research and governmental efforts to implement an IFM system within the indigenous territories of Raposa Serra do Sol, Canaima National Park, North and South Rupununi to reform zero-fire policies[^92,97^](https://www.zotero.org/google-docs/?5qOGpG).

## Canada

While all burns represented in the GlobalRx dataset do not specify a burn objective, most Canadian prescribed burns conducted in national parks are done so for the purposes of ecological treatment, fuel load management, and hazard abatement[^98^](https://www.zotero.org/google-docs/?n6NtzX). We note that Canada does not appear to maintain a single centralized system for reporting prescribed fires. Thus, there are many prescribed burns managed provincially or at a district level, primarily for fuel load management and hazard abatement[^99–101^](https://www.zotero.org/google-docs/?hOGcvq) that do not appear in GlobalRx.

Across Canada, a significant portion of prescribed burns are conducted for silvicultural purposes after clear-cut logging, particularly in British Columbia (BC) and Ontario. Burning objectives in these forests can include increasing forest productivity through eliminating plant competition, renewing soil nutrients, and pest control. For example, in BC, prescribed burns are conducted for silvicultural purposes to rehabilitate “non-satisfactorily restocked lands (NSR)”. This has been applied to stands of spruce sup-alpine fir and lodgepole pine, which constitute a large portion of the North Central Rockies forest ecoregion[^102,103^](https://www.zotero.org/google-docs/?2hcLgT).

258 burns covering 60,093 ha in GlobalRx were recorded in the Temperate Conifer Forests biome, which includes the Alberta Mountain forests, Alberta-British Columbia foothills forests, and North Central Rockies forests. These ecoregions were historically of mixed-severity fire regimes, which are primarily characterized by strong landscape heterogeneity that are uneven-aged and multi-layered, though historical fire suppression has led to a transition to a fire regime characterized by stand-replacing fires[^104^](https://www.zotero.org/google-docs/?v8klyg). The Flathead and Pend d'Oreille tribes and Kootenai people maintained this heterogeneous landscape by burning at a moderate frequency (<10 year fire return interval) primarily in early spring or fall, for purposes of stimulating grasses for grazing, collecting firewood, maintaining trails and campsites, and to influence game movement[^105^](https://www.zotero.org/google-docs/?OeKUt3). Prescribed burning is commonly conducted in Banff National Park, located in these ecoregions, for ecological conservation, including to induce spruce and fir mortality (pers. comm., Crystal Kolden), conserve whitebark pine (Pinus albicaulis) from white pine blister rust[^106,107^](https://www.zotero.org/google-docs/?IAPbDj), and increase elk forage[^108^](https://www.zotero.org/google-docs/?Am2892). Burns are also conducted to reduce fuel loads near structures and campsites and decrease the probability of crown fire in lodgepole pine stands[^109^](https://www.zotero.org/google-docs/?UZ3yse). Prescribed burns have also been conducted to benefit red and white pine forests in the eastern forest–boreal transition ecoregion[^110^](https://www.zotero.org/google-docs/?6znaeA). The majority of burns in this biome were conducted between March and June, though another significant period for burning occurs between September and December.

161 of the burns occurred in the Boreal Forests/Taiga biome, which include Central Canadian Shield forests, Eastern Canadian forests, Mid-Continental Canadian forests, Midwestern Canadian Shield forests, Muskwa-Slave Lake forests, Northern Canadian Shield taiga, and Northwest Territories taiga. While the number of burns in this region is fewer than other biomes, the total area burned in this biome (91,956 Ha) greatly exceeds that of other biomes. This fact is reflected in the wildfire activity of the region, as this biome is responsible for the majority of area burned and largest share of large fires (>200 ha) in Canada[^111^](https://www.zotero.org/google-docs/?bXUDkU). The boreal fire regime is often characterized by large and infrequent high-intensity crown fires that are often stand-replacing, with the fire season typically spanning from May to September and peaking in June and July[^112^](https://www.zotero.org/google-docs/?q2SpEC). While lightning is the primary cause of ignition for these large fires[^113^](https://www.zotero.org/google-docs/?q8KVIm), Indigenous peoples in the boreal also influenced the landscape, conducting more frequent and smaller, lower to moderate intensity burns on the landscape for “maintenance of meadows, opening up grasslands, burning deadwood, extending the growing season, obtaining firewood, improving settlements and campsite areas, making and maintaining trails, opening up animal habitat, increasing berry production, reducing pests, religious reasons, and esthetic benefits[^114^](https://www.zotero.org/google-docs/?AiPcqn)". These fires were conducted primarily in the spring, after snow cover melted from the meadows, though burns were occasionally conducted in late autumn as well[^115^](https://www.zotero.org/google-docs/?g9SmuT). GlobalRx burns were most frequently conducted between April and June, though some burns were also conducted from August through November.

75 of the prescribed burns occurred in the Temperate Grasslands, Savannas & Shrublands biomes, covering 51,250 Ha in Canadian Aspen Forests and Parklands and Northern Short Grasslands ecoregions. Before white settlement, the Parkland Cree people burned in Canadian Aspen Forests and Parklands with low to moderate fires at high frequency (a fire return interval of 3-15 years) to manipulate game movement, increase soil fertility, and reduce the fire hazard imposed by dead brush and grasses[^116^](https://www.zotero.org/google-docs/?cpomoO). There is also evidence that annual, more severe fires prevented woody encroachment of aspens onto the grasslands, allowing grasses and small brush to flourish[^117^](https://www.zotero.org/google-docs/?lgQGEC). In the Northern Short Grasslands, Northern Plains tribes maintained the diversity and health of the grasslands using frequent, fast-burning (<1 day) fires of small to moderate scale to manipulate movement of bison, remove dead grass, and improve forage for horses[^118–121^](https://www.zotero.org/google-docs/?gFrT3S). These fires likely occurred during nearly every month of the year but occurred the most frequently in the spring and fall, peaking in April and October, when drier weather coincided with the presence of dried, cured grasses. Larger, lightning-caused fires dominated summer months, particularly from June to August[^118,119^](https://www.zotero.org/google-docs/?npgzZR). Similarly, burns recorded in GlobalRx were conducted almost exclusively between mid-March and mid-June, with some fires conducted from September to October. Today, burning in these grasslands is facilitated by prescribed fire associations such as the Canadian Prairies Prescribed Fire Exchange, which utilizes prescribed burning for conservation, traditional practice, and public safety in both public and private lands[^122^](https://www.zotero.org/google-docs/?6KPlZU).

62 of the prescribed burns occurred in the Temperate Broadleaf & Mixed Forests biome, covering 2,995 Ha. This biome includes Eastern Great Lakes lowland forests, Eastern forest-boreal transition, New England-Acadian forests, Southern Great Lakes forests, and Western Great Lakes forests. White pine and red pine, tree species commonly found in this biome, are known to be fire dependent, requiring moderate intensity fires every 30-60 years to improve the seedbed, kill off competitive vegetation, and allow time for seedlings to mature enough to develop a cambial thickness capable of surviving moderate intensity fires[^110,123,124^](https://www.zotero.org/google-docs/?ToHf4T).

Today, Indigenous fire practices face many colonial barriers to reimplementation, including but not limited to the lack of understanding of cultural burning as an indigenous community practice, power imbalances in knowledge frameworks, decontextualized attempts at engagement with Indigenous experts that further marginalize Indigenous communities, the lack of and inaccessibility to accreditation for cultural burning, and bureaucratic and jurisdictional hurdles to obtaining authorization for cultural burns[^125^](https://www.zotero.org/google-docs/?MVpBU7). However, there is a growing recognition of the need to return to indigenous fire stewardship, especially in the wake of the increasing number of megafires resulting from nearly a century of colonial forest mismanagement[^126^](https://www.zotero.org/google-docs/?UIg9Lc). For example, in British Columbia, cultural burning is being revitalized by Yunesit’in and Xeni Gwet’in First Nations on Yunesit’in community and Tsilhqot’in Title lands by the [Gathering Voices Society](https://www.gatheringvoices.com/). In northern Saskatchewan, the establishment of the Prince Albert Grand Council Wildfire Task Force allows First Nations to exert jurisdiction and authority over how fires are managed on their traditional territories.

## France

Fire use for rangeland management has long been used in the French Pyrenees for centuries, despite forestry laws banning its use dating back to the 19th century. Farmers and shepherds practiced seasonal transhumance, moving animals to higher elevation pastures in the summer, while utilizing mid-elevation pastures in the fall and spring. These mid-elevation pastures were commonly the target for burning to prepare forage for the spring between January and May, and regular burning was typically conducted every 3-5 years to prevent woody encroachment[^127^](https://www.zotero.org/google-docs/?8FwRcc). High stocking rates of cattle and sheep coupled with periodic fire use and mechanical clearing generally prevented shrub encroachment. However, rural abandonment and the decline of traditional agriculture and stockbreeding, especially in mountain areas, resulted in shrub encroachment into the lowlands, rendering previous burning practices difficult to control. Fires, often set illegally and without proper controls, threaten the mountain forest and also pose a serious risk to tourists and its inhabitants. Prescribed burning was first considered as an efficient, low cost method in the 1980s to address the problem of fuel accumulation from shrub encroachment and to prevent uncontrolled fires in the region[^128^](https://www.zotero.org/google-docs/?EaP0b2).

In France, prescribed burning policies and implementation is managed regionally through each administrative region, or département. The importance of certain burn objectives differ by département, but in the Pyrenees are primarily related to pastoral burning for stock breeders to improve or maintain pasture quality, and hazardous fuel reduction to combat fuel accumulation from shrubland encroachment resulting from a decline in mountain agriculture and stock breeding beginning in the 20th century[^128,129^](https://www.zotero.org/google-docs/?gHPhU6). Prescribed burning programs and policies also vary by region, ranging from the use of an official, centralized burning team of specialists in the Pyrénées-Orientales on the Mediterranean side of the Pyrenees to more localized burning committees comprising stock breeders and farmers in the Pyrénées-Atlantiques on the Atlantic side.

There are active fire management programs in Pyrénées-Orientales, Hautes-Pyrénées, Ariège, and Pyrénées-Atlantiques, though with variable success and effectiveness, dependent upon the economic and ecological landscape as well as the regional climate. For example, in the Pyrénées-Orientales, stock breeding and agriculture declined more significantly relative to other parts of the Pyrenees, resulting in high fuel accumulation in shrublands. These hazardous fuel conditions also coincided with a strong cold and dry northerly wind (tramontane). The risk associated with burning was thus conducive to the establishment of a specialist prescribed burning group, from whom local stock breeders, associations, and municipalities can request intervention. Additionally, the Mediterranean climate brings frequent dry periods during the winter that are conducive to burning. The longer window for burning affords the burn team the flexibility to conduct throughout the winter, thus allowing a single team to address multiple requests for burning interventions. In contrast, in Ariège, which has a more humid, sub-Atlantic climate, burning is limited to fewer days. To meet the burning needs of stock breeders would thus require burns to occur simultaneously over the few good burning days, which would be difficult for a centralized team to accomplish[^128^](https://www.zotero.org/google-docs/?RDSLHM). In Hautes-Pyrénées, the decline in stockbreeding has not been as significant, and thus the local knowledge to implement controlled burning has persisted in this region. Following an ineffective fire ban, a policy based more on local management was implemented, leading to the formation of prescribed burning committees which have facilitated continued, safer controlled fire use, and an increase in the number of burns that are declared[^128^](https://www.zotero.org/google-docs/?rcDcXs).

The majority of burns are conducted at mountain level, likely accounting for the 65% of burns in the dataset that were conducted in the Temperate Broadleaf & Mixed Forests biome in the Pyrenees conifer and mixed forests ecoregion. This encompasses the Pyrenees mountain range and contains the mountain pastures where most pastoral activity and stock grazing occurs. Nearly half of all fires occurred on cropland. Some burns also occur in shrubland areas close to farms or in low summer pasture areas, likely accounting for the 35% of burns occurring in the Mediterranean Forests, Woodlands & Scrub[^130^](https://www.zotero.org/google-docs/?yLuR4g).

All burns were conducted between October and April, with 76% (1,104) of those burns occurring between January and March. Burns are typically conducted in dry periods during the winter and early spring, before the Mediterranean summer fire season[^129^](https://www.zotero.org/google-docs/?V8kdq0). The dataset reflects the Pyrénées-Orientales prescribed burning team operations, which occur between November and March, with heavy equipment operations occurring primarily between January and March.

## Germany

Like much of central Europe, Germany’s landscape has long been shaped by anthropogenic fire through swidden agricultural systems and animal husbandry. In combination with grazing, mowing, and cutting of the native vegetation, these land uses resulted in vast areas of Calluna dominated heathlands and open grasslands. However, such cultural landscapes are threatened by rural abandonment as farming and livestock rearing have become less profitable, leading to the secondary succession of trees and shrubs at the expense of a more open vegetation structure[^131,132^](https://www.zotero.org/google-docs/?kq08mb).

Today, prescribed burning in Germany is limited, conducted primarily on an experimental basis in meadow grasslands, heathlands, and some forest plantations and peat bogs in Nordrhein-Westfalen[^130^](https://www.zotero.org/google-docs/?703vzs), Baden-Württemberg[^131,133^](https://www.zotero.org/google-docs/?fjd8y3), and Niedersachsen[^134,135^](https://www.zotero.org/google-docs/?9AMCx4). Broadcast burning was prohibited under the Federal Nature Conservation Act in 1975[^136^](https://www.zotero.org/google-docs/?YkCfzh), with the exception of its use for military training. This resulted in the further expansion of woody vegetation[^131^](https://www.zotero.org/google-docs/?NqKFIO). However, it may presently be implemented with special permission from municipal authorities and under strict guidelines[^130,131^](https://www.zotero.org/google-docs/?WKazKn). Burns were generally conducted in the late winter (February) to minimize any thermal effects to the soil[^130^](https://www.zotero.org/google-docs/?brYi4Q). Results from the experimental burns were generally positive, with results ranging from successful short-term woody reduction and no effects on species diversity in meadow grasslands[^131^](https://www.zotero.org/google-docs/?AdbG5z) to better-than-expected outcomes observed in heathlands for improving bird, insect, and arachnid populations, increasing plant species diversity, reducing birch encroachment, and reducing senescent Calluna stands[^130^](https://www.zotero.org/google-docs/?KKZXBq).

## Italy

Fire has long been used in Italy for agriculture, silviculture, natural resource and pastoral management since the Greco-Roman era. Broadleaf forests, which dominate in Italy, have a long history of management, with over half of them having been maintained through understory burning, coppicing, or pollarding for hundreds of years prior to modern times[^137^](https://www.zotero.org/google-docs/?7JdRdi). However, like much of southern Europe, industrialization, urbanization, and rural depopulation of mountain areas beginning in the 1950s (post-WWII era) led to the decline of many traditional agricultural, silvicultural, and pastoral practices. At the same time, the development of the timber industry led to increased fire suppression and the prohibition of agro-pastoral fire[^138^](https://www.zotero.org/google-docs/?HCZoOd). This has resulted in the expansion of forests and shrubland at the expense of agricultural land, grasslands, and pastures, resulting in higher incidences of large fires[^139^](https://www.zotero.org/google-docs/?Yd3wlb).

Presently, prescribed burning in Italy is managed on a regional basis, with each region setting their own objectives and regulations for its practice as a part of a compulsory wildfire risk management plan, often in collaboration with the forest sector[^140^](https://www.zotero.org/google-docs/?JrvAjJ). 14 of the 20 regions of Italy list prescribed burning as a management practice, with wildfire hazard reduction as the most commonly stated objective. Many of the first experimental prescribed burns were conducted in the 2000s, primarily for this purpose in even-aged plantation Mediterranean pine forests consisting mostly of *Pinus pinaster*, *Pinus halepensis*, *Pinus pinea*, and *Pinus canariensis*, in regions in which there exists a regional legal framework regulating prescribed burning[^138^](https://www.zotero.org/google-docs/?QImkLk).

Pastoral and stubble burning are some of the most common public uses of fire across Italy, especially in grasslands and oak woodlands, and are common sources of ignitions resulting in wildfires[^138^](https://www.zotero.org/google-docs/?8K6GVd). Thus, the former is often worked into burn plans in collaboration with local shepherds so that local uses for fire may be carried out in a more closely monitored fashion. In Northern and Alpine Italy, prescribed burning and grazing are used in collaboration with local shepherds for the conservation of meadows and grasslands (Friuli-Venezia Giulia, Liguria), and heathland species such as Calluna vulgaris (heather) (Piedmont), which are threatened by invasive grasses and encroaching forests and shrublands due to the decline in traditional grazing and pastoral fire practices[^141–143^](https://www.zotero.org/google-docs/?4TAr37).

## Japan

Japan’s forests are not generally fire prone - the country’s climate is typically wet and mild, with high precipitation during the wet season (June-July, end of September), and its forests are dominated by broadleaves, which do not burn easily. Lightning fires are rare, as lightning strikes typically coincide with precipitation; hence most forest fires are caused by human ignitions. The most flammable regions of Japan are located in the western portion, where secondary forests consisting primarily of Japanese red pine (*Pinus densiflora*), and to a lesser extent Japanese black pine (*Pinus thunbergii*) and Okinawa pine (*Pinus lutchensis*), provide a more flammable landscape. The decline of forest management and utilization due to the privatization of forests and the decreasing economic returns of domestic timber resources starting in the late 19th century has contributed to the proliferation of forest fires due to surface fuel accumulation[^144,145^](https://www.zotero.org/google-docs/?m5aPf0). The most fire-prone region of Japan is located in the Seto Inland Sea region, located in the south of Japan, where climate conditions during the summer may resemble Mediterranean conditions, with long, hot, and dry summers[^144^](https://www.zotero.org/google-docs/?wh6DHY).

Prescribed burning is uncommon in Japanese forests, and the majority of preventative fire measures consist of preventing ignitions, preventative logging, and removal of surface fuels[^144^](https://www.zotero.org/google-docs/?89Ippi). The majority of recorded burns were conducted in grasslands in the southwest of the country, in areas often consisting of Japanese pampas grass (*Miscanthus sinensis*) or Amur silver grass (*M. sacchariflorus*)[^146^](https://www.zotero.org/google-docs/?aurIxf). In many areas, burning is carried out annually, often by local residents as a part of a yearly ritual or tradition. For example, in Wakakusa-yama (Nara prefecture, 66 records), dead grass atop Mount Wakakusa is burned every year in January as a part of a festival known as Yamayaki[^147^](https://www.zotero.org/google-docs/?bbTGB0). In Akiyoshidai Quasi-National Park (Yamaguchi prefecture, 46 records), annual burnings are carried out in February to conserve the grasslands[^148^](https://www.zotero.org/google-docs/?rOMieN). In the Sengokuhara Pampas Grass Field (Kanagawa prefecture, 30 records) controlled burning is used to clear senescent vegetation every spring[^149^](https://www.zotero.org/google-docs/?Czqcxm). In Mount Sanbe (Shimane prefecture), fire is applied every spring following snowmelt to maintain the grass cover and reduce wildfire risk[^150^](https://www.zotero.org/google-docs/?eHhFjc). Grassland burning had been conducted traditionally for swidden agriculture, cattle grazing, and to prevent woody encroachment and conserve grasslands, which have been increasingly abandoned since the 1960s due to land development[^151–154^](https://www.zotero.org/google-docs/?9rsWEg).

## Mexico

Mexico is home to an incredibly diverse range of ecosystems, at least 50% of which are either fire-dependent (adapted to high frequency fires) or fire-influenced (adapted to low frequency fires). Of the fire-adapted ecosystems, pine and oak forests along the East, West, and South Sierra Madre mountains constitute the majority of area, followed by shrublands in central Mexico, grasslands and savannas, wet prairies, and palm lands. The fire-sensitive ecosystems lie primarily east of the Sierra Madre Oriental and along the Gulf of Mexico, in the Tropical & Subtropical Moist Broadleaf Forests biome[^155^](https://www.zotero.org/google-docs/?gajS2Y).

Fire was historically and is still commonly used today primarily for swidden agriculture, often following the traditional milpa system, a system in which maize, beans, and corn are cultivated in rotation and secondary succession is allowed to occur on fallowed fields[^156^](https://www.zotero.org/google-docs/?w1y5RN). Fire was also used for livestock forage, hunting, and clearing brush[^157^](https://www.zotero.org/google-docs/?JMW2Wv), and the forest structure maintained by this pattern of burning and land management has been linked to floral and faunal diversity due to the presence of multiple successional stages[^156,158,159^](https://www.zotero.org/google-docs/?CcaBQ8). Following the introduction of cattle, sheep, and other livestock brought about by Spanish colonization, fire was also used to produce livestock forage[^160^](https://www.zotero.org/google-docs/?1dBt0P). Following the Mexican Revolution, much of the country’s lands were divided into ejidos, parcels of land upon which community members have usufruct rights (the right to cultivate but not own the land). Ejidos today constitute approximately half of the country’s land area[^161^](https://www.zotero.org/google-docs/?FczR3l), and many land management decisions are made at this scale[^162–164^](https://www.zotero.org/google-docs/?z3WiwZ).

Today, agriculture constitutes over 80% of the burned area in the country[^160^](https://www.zotero.org/google-docs/?9STzf4). Through the 1900s, official government policies did not recognize the benefits of fire use on the landscape, and uniformly viewed fire - especially agricultural fires - as a destructive force, and those who set them - predominantly rural farming communities - as irresponsible, irrational, and immoral[^165^](https://www.zotero.org/google-docs/?VCcuLk). Additionally, the changes in land tenure, which displaced Indigenous populations and shifted land use from subsistence living to market-oriented activities, such as cattle farming, resulted in reduced overall anthropogenic fire but an increase in more destructive fire use[^160,166,167^](https://www.zotero.org/google-docs/?uUETUT). Beginning in the early 2000s, and brought about in part by the record-breaking 1998 wildfire season, a movement to reformulate fire management was undertaken by federal agencies, NGOs, and academics based on fire ecology and integrated fire management (IFM) that coupled ecological, silvicultural, and fire risk reduction objectives with the agricultural and resource needs of local communities[^160^](https://www.zotero.org/google-docs/?PtwYHN).

Prescribed burning in Mexico is primarily managed by CONAFOR and Comisión Nacional de Áreas Naturales Protegidas (CONANP), often in partnership with NGOs, academic institutions, and local municipalities and communities[^168^](https://www.zotero.org/google-docs/?iF0z1D). Many IFM programs are based in biosphere reserves that encompass fire-sensitive tropical regions, primarily in the southern states such as Chiapas, Oaxaca, and Campeche[^155^](https://www.zotero.org/google-docs/?ZwvSRY). However, forest and fire management is also implemented by ejido communities throughout Mexico (pers. comm., FIRE-ADAPT UNSIJ). Such IFM programs work with *ejido* communities to develop a system of fire use that maintains traditional agroecosystem and swidden agriculture practices[^162^](https://www.zotero.org/google-docs/?oPSAHx). Note that data in GlobalRx do not adequately reflect the distribution or number of burns in the country.

## Portugal

Like other Mediterranean regions, Portugal’s landscape has a long history of human-induced fire, which was used for agricultural cultivation, plant domestication, pasture renewal, and land clearing for thousands of years. Many plant species likely evolved to be fire adapted[^169^](https://www.zotero.org/google-docs/?Z6m04l), including commercially important trees, such as the cork oak. Between the 19th and 20th centuries, timber demand led to vast reforestation and afforestation efforts, resulting in the progressive occupation of uncultivated lands, and the expansion of pine forest area primarily consisting of Pinus pinaster. These afforestation efforts also coincided with accelerating rural industrial development, ultimately resulting in rural abandonment, decreases in grazing and wood gathering, and thus an increase in accumulated fuel[^170^](https://www.zotero.org/google-docs/?h4Q6xF). Additionally, the pine forests were cultivated at the expense of grazed shrublands and cultivated fields, resulting in increased wildfire risk[^171–173^](https://www.zotero.org/google-docs/?EfZ8Uy).

In the 1970s, the idea of reintroducing fire to the landscape gained traction following visits from American forest technician Edwin Komarek, and in 1982 a fuel reduction program using prescribed fire was implemented in over 55% of the communal forest area in the region, primarily in Pinus pinaster stands[^173^](https://www.zotero.org/google-docs/?vbfaBt). The use of prescribed fire was further revived in the 2000s following devastating wildfires in 2003 and 2005, with designated legislation prescribed burning existing since 2006 (Regulamento do Fogo Técnico). Prescribed burns are now nationally managed and recorded by the ICNF, though other agencies such as the Agência de Gestão Integrada do Fogo Rural (AGIF) and Fire Use and Analysis Group (GAUF) also provide technical and logistical support.

As it is particularly prone to severe forest fires, prescribed burns in Portugal are carried out overwhelmingly for hazardous fuel reduction purposes, accounting for over 80% of burns conducted between 2006-2016[^129^](https://www.zotero.org/google-docs/?z4Se9m). Prescribed burning for hazardous fuel reduction in Portugal began in 1982 but did not gain sustained, widespread use until 2004, when forestry experts first developed guidance and training for prescribed burning. Subsequent legislation in 2006 that stipulated its use for preventing forest fires also contributed to its wider adoption. Prescribed burns are now nationally managed and recorded by the Instituto da Conservação da Natureza e das Florestas (ICNF), though other agencies such as the Agência de Gestão Integrada do Fogo Rural (AGIF) and Fire Use and Analysis Group (GAUF) also provide technical and logistical support[^129,130^](https://www.zotero.org/google-docs/?EBL01v).

In GlobalRx, 54% of burns occur in the Temperate Broadleaf Forest biome, and 46% occur in the Mediterranean Forests, Woodlands & Scrub biome. Pinus pinaster (maritime pine), which constitutes 23% of the forested area in Portugal, is native to the ecoregions in both of these biomes and is commonly targeted in prescribed burns due to its fire tolerance[^173^](https://www.zotero.org/google-docs/?ea4ncL), high flammability[^174^](https://www.zotero.org/google-docs/?WWPVCg), and prevalence due to afforestation efforts in the 1950s[^171^](https://www.zotero.org/google-docs/?jKXWtM). Indeed, GlobalRx burns coincide with regions where Pinus pinaster is present[^175^](https://www.zotero.org/google-docs/?f0fDjY).

The majority of the GlobalRx burns occur in Northwest Portugal between October and May, before the summer wildfire season, with 52% of the burns occurring between February and April. Burns in both biomes exhibit the same seasonal burn pattern. This period is favorable for burning in pine stands, as it tends to be colder and drier. Precipitation and high fuel moisture are the predominant limitations to burning during the winter, while low winds due to the Iberian high pressure system can also limit a team’s ability to stay within the fire prescription[^176^](https://www.zotero.org/google-docs/?Qw3DBv).

## Russia

Some of the earliest records of fire use in Russia date back to the 6th Century, when cured grass burning was used by cattle breeders in the steppe regions to increase grazing land productivity[^177^](https://www.zotero.org/google-docs/?6Fw0IV). The Indigenous peoples of Yakutia had extensive ecological knowledge on fire use, using it to maintain pastures for horse breeding, create lakes from permafrost, and clear vegetative debris[^178^](https://www.zotero.org/google-docs/?qtE4IH). In the 16th-18th Centuries, the Russians colonized Siberia and the Far East, and parcels of both forested and non-forested land were allocated to settlers. The settlers transformed the landscape by practicing slash-and-burn agriculture, and fire was used for clearing and fertilizing the land, sowing crops, and grazing cattle. Peasants in the taiga regions also burned in Scots pine stands (Pinus sylvestris) to increase production of red whortleberry (Vaccinium vitis-idaea) and in deciduous broadleaf forests to improve conditions for beekeeping[^179^](https://www.zotero.org/google-docs/?aRalTd). The 19th-20th Centuries brought about the commercial exploitation of Russia’s forests, and the first uses of prescribed burning by forestry professionals in Russia date back to the early 1900s, with some of the earliest studies reporting fire’s positive influence on Scots pine stand regeneration[^179^](https://www.zotero.org/google-docs/?RQmWDL). Prescribed burning was also used to clear logging slash, and later as a form of pest control in Siberia against pine looper and Siberian moth. Studies published from the 1950s-1980s also describe the benefits of prescribed burning on forest regeneration and hazard reduction. However, broadcast burning was prohibited on logging sites in 1993 (though pile burning was still permitted)[^180^](https://www.zotero.org/google-docs/?MqO2ur), and agricultural fires are also forbidden, though satellite evidence suggests it is still a widespread practice[^181^](https://www.zotero.org/google-docs/?S9Mjr5).

However, research efforts among scientists and forestry authorities from Europe, North America, and Russia beginning in 1993 as a part of the Fire Research Campaign Asia-North (FIRESCAN) sought to replace fire exclusion policies that were in place at the time and implement integrated fire management approaches that included the use of natural fires and prescribed burning. Between 1997 and the present, FIRESCAN conducted numerous prescribed experiments in various ecoregions of Siberia - including plains and mountain dark conifer forests, Scots pine stands, and the forest-steppe zone - and also commenced the 200-year Bor Forest Island Experiment, which will run from 1992-2193 to evaluate the long-term effects of prescribed fire[^179^](https://www.zotero.org/google-docs/?E1TXdO). Legislation outlining guidelines for prescribed burning was introduced in 2019, though its practice is restricted to trained specialists[^182^](https://www.zotero.org/google-docs/?NLQst9).

Overall, 52% (11,534 burns) of burns occur in the Boreal Forests/Taiga biome, 18% (4,071 burns) in Temperate Broadleaf and Mixed Forests, and 16% (3,587 burns) in Temperate Grasslands, Savannas & Shrublands. 40% of prescribed burns occur in oblasts falling within the Lake Baikal Basin (Zabaykalsky Krai, Buryatia), a region that is heavily exploited for logging, both legally and illegally[^183,184^](https://www.zotero.org/google-docs/?GGLeUo). The burns in these regions occur predominantly in needleleaf deciduous forests (65%, 5,702 burns), which are composed of Dahurian and Siberian larch (Larix gmelinii, Larix sibirica), Siberian and Scots pine (Pinus sibirica, Pinus sylvestris), Siberian spruce (Picea obovata), and Siberian fir (Abies sibirica), and to a lesser extent mosaic forest-shrubland/grassland (18%, 1,610 burns)[^185^](https://www.zotero.org/google-docs/?1d8oNY). Prescribed burns along the southern border of Russia generally occur along the Trans-Siberian Railroad, indicating some correlation with prescribed burn location and major population centers. Many of these areas also coincide with areas where forestry is the main driver of tree cover loss; thus, it is possible that many of these burns are related to logging activity[^186,187^](https://www.zotero.org/google-docs/?KKoylh). 14% of burns occur in the Sakha Republic (Yakutia) just east of Siberia, which is the most heavily forested region of Russia[^188^](https://www.zotero.org/google-docs/?lkkwUQ). This region also frequently experiences some of the highest wildfire activity[^189^](https://www.zotero.org/google-docs/?ewIcu4). This region is predominantly Boreal Forest/Taiga, with 3,133 burns in this oblast (>99%) occurring in this biome. Forests in this region consist mostly of larch in the north and pine and fir in the south. Unlike the other oblasts, the majority of burns in Sakha occur in fall, with 67% of burns occurring in September and October.

The vast majority (82%) of fires burned between March and May, with 63% of those fires occurring in April. The majority of the remaining fires were conducted between September and November. This was true for all biomes. The seasonality of the prescribed fires reflects the inverse of the wildfire season, which generally spans from May to September and peaks between June and July. The ecoregions where most of the burns occur have a subarctic climate, which is characterized by long and cold winters with little precipitation and short, warm to cool summers.

The role of agricultural burning in Russia is likely understated by GlobalRx. Only 97 fires (0.004%) were classified as agricultural burns. However, the prevalence of agricultural burning in Russia is well-documented, with some estimates indicating that Russia is responsible for nearly a third of agricultural burning emissions globally[^181,190,191^](https://www.zotero.org/google-docs/?IvJphv).

## South Africa

Prescribed burning in South Africa is conducted primarily in savannas, located in the northeast of the country, and the fynbos shrublands, located along the southern coast of the country. Savanna and shrubland burning was a common practice for thousands of years, with both indigenous peoples and later European colonists using this practice to improve forage for cattle, sheep, and other livestock through the early 1900s, despite its illegality[^192^](https://www.zotero.org/google-docs/?cf0Rdi). Fire was also historically a frequent and important disturbance in the fynbos shrubland ecosystem[^193,194^](https://www.zotero.org/google-docs/?Dloz0p). However, the condemnation of fire use by the Drought Investigation Commission in 1926 under the guise of water resource protection, and the subsequent establishment of the Fire Protection Committees under the 1946 Soil Conservation Act, resulted in the subsequent institution of fire suppression policies until the mid-1950s[^195^](https://www.zotero.org/google-docs/?7rwSIg). However, a growing recognition of the importance of fire in the ecological cycles in both savanna grasslands and the fynbos shrublands led to the reintroduction of fire in the form of managed fires and prescribed burning in the 1950s-1960s.

The first prescribed burning experiments in South Africa were conducted in Kruger National Park (KNP) in 1957, and these experiments have since continued and evolved in strategy: initially managed with 3-year fixed-rotation burning, management strategies now include managed wildfires and patch mosaic burning to reflect evolving ecological research paradigms recognizing the role of heterogeneity in promoting biodiversity[^196^](https://www.zotero.org/google-docs/?9vBxpo). 975 burns (92%) in GlobalRx were conducted in KNP, primarily in the Zambezian and Mopane woodlands ecoregion, or in adjacent Montane Grasslands & Shrublands. These burns primarily serve the purpose of ecological management and research, with investigations on the effects of prescribed fire on floral and faunal distribution and composition, soil, emissions, and fire behavior[^196^](https://www.zotero.org/google-docs/?RC6MRu). Prescribed burns in savannas both within and adjacent to KNP have also been used to address the issue of bush encroachment, resulting from a combination of fire suppression and overgrazing[^195,197,198^](https://www.zotero.org/google-docs/?FsRe4H).

Prescribed burning was introduced to the fynbos ecosystems in the 1970s, after the recognition that a policy of complete fire suppression was not only impractical but also ecologically detrimental to many endemic and fire-adapted fynbos plant species, particularly the rare and iconic species Serruria florida (blooming bride) and Orathamus zeyheri (marsh rose)[^192,193^](https://www.zotero.org/google-docs/?GWBhKH). Prescribed burning objectives (set under the 1970 Mountain Catchment Act) initially included water resource management and nature conservation[^192,194^](https://www.zotero.org/google-docs/?q0rTzW). Wildfire hazard reduction was initially also explored as an objective, but subsequent studies indicated that prescribed burning did not reduce the incidence or extent of wildfires, since fires in fynbos shrublands are ignition rather than fuel limited due to rapid post-fire fuel accumulation rates. This finding is consistent with studies in other Mediterranean shrublands[^195^](https://www.zotero.org/google-docs/?cpTbpn). 63 burns (6%) were conducted in fynbos and renosterveld ecoregions.

Prescribed burning is used to combat but also simultaneously severely complicated by the presence of alien species, especially in the fynbos ecosystems, which have some of the highest levels of endemism in the world. Of particular concern to prescribed burning are the eucalyptus and pine species that were introduced in timber tree plantations in the early 1900s[^199,200^](https://www.zotero.org/google-docs/?oCr6vx), and acacia and hakea species, which were introduced as ornamental plants[^201^](https://www.zotero.org/google-docs/?lg1Ehz). These species are all fire adapted and regenerate vigorously after fire, thus requiring intensive and consistent post-fire treatment in order to be effective[^195^](https://www.zotero.org/google-docs/?Ty4Ewe).

## Spain

Spain’s fuel condition is severely complicated by a long legacy of extensive reforestation and afforestation efforts, spanning over a century from the late-19th to late-20th centuries (and perpetuated into the 1990s by the Common Agricultural Policy), which together covered some 5 million ha of land, mostly with pines (most Pinus pinaster, Pinus sylvestris, Pinus nigra), Eucalyptus, and other hardwoods (namely Quercus). Much of the land acquired by the state and used for such efforts consisted primarily of grasslands, pastures, and agricultural fields; this not only served to homogenize the landscape and increase fuel continuity, but also accelerate rural depopulation[^202^](https://www.zotero.org/google-docs/?sbUemV). The result of these factors has led to a marked change in the fire regime, with wildfires increasing drastically in both frequency and size beginning in the 1970s[^203,204^](https://www.zotero.org/google-docs/?qjlMdn).

Prescribed burning research was first explored in plantations in the 1980s, but its use was limited until the establishment of programs and teams of fire experts who could specialize in applying fire in more localized contexts. Official prescribed burning in Spain started in 1998 in the northern regions following devastating wildfires seasons in 1994 and 1998. Burning objectives in Spain are regionally dependent, but often involve some component of hazardous fuel reduction. In northern regions, pasture and shrubland management for grazing is a major reason for burning. Like France, prescribed burns in this region serve as a surrogate for traditional burning practices in areas where it has declined due to rural depopulation or where current fuel conditions present a considerable hazard to its practice. In central and southern Spain (Anadalucia) as well as the Canary Islands, prescribed burning is used for fuel reduction and habitat management[^129^](https://www.zotero.org/google-docs/?QWda5c). Prescribed burns in northwestern and central Spain are planned and managed through the Integral Wildfire Prevention Teams (EPRIF), a national program established in 1998. EPRIF deploys teams of 2-4 fire specialists into high-risk rural regions, where they work with local community members to establish a burn program that suits the region’s needs while also minimizing wildfire risk[^130^](https://www.zotero.org/google-docs/?ZvK4lT). In Catalonia, prescribed burns have been used as a fire management tool since 1998 by the Group of Support to Forest Actions (GRAF), a team of specialized wildland firefighters. Most prescribed burns in Catalonia are conducted for hazardous fuel reduction due to the region’s high wildfire risk posed by Mediterranean climate, Foehn winds, and relatively high woodland growth rate, though burns are also commonly conducted for pastoral burning and habitat management[^129,204^](https://www.zotero.org/google-docs/?RvAYo6).

Overall, 48% of the prescribed burns occurred in the Mediterranean Forests, Woodlands, and Scrub biome, while 52% occurred in the Temperate Broadleaf and Mixed Forests biome. Northwestern Spain (Galicia and Asturias) is dominated by the Temperate Broadleaf and Mixed Forests biome, and 83% of all burns in this biome occur in this region. 82% of prescribed burns here are conducted in broadleaf deciduous forests, which are dominated by native hardwoods such as English Oak (*Quercus robur*), Pyrenean oak (*Quercus pyrenaica*), and Sweet chestnut (*Castanea sativa*)[^205,206^](https://www.zotero.org/google-docs/?pr3x8y). In contrast, 77% of all burns conducted in the Mediterranean Forests, Woodlands, and Scrub biome occur in Catalonia. Of these burns, 30% occur in needleleaf evergreen forests, 29% in cropland, and 24% in shrubland or sparse vegetation.

Prescribed burns tended to occur most frequently in late winter/early spring, with 50% of all burns occurring in either February or March. Fall burning accounted for 15% of burns. These seasons tend to correspond with mild weather, with moderate rainfall and before the onset of spring Foehn winds in the Mediterranean. Burns tended to occur least frequently in summer months, except for burns on croplands, which occurred most frequently in July and August. Burns in Galicia occurred more frequently in the spring than in the fall or winter, when traditional pastoral burnings typically occur.

## Sweden

Prescribed burning in Sweden was used widely in the 1950s-1960s for silvicultural purposes, as a method of forest regeneration. Its mainstream adoption can be credited to Joel Wretlind, a forest manager who conducted some of the first scientific experiments on prescribed burning in the 1920s[^207^](https://www.zotero.org/google-docs/?VQ5MWf). At the time, forest health had been severely degraded by fire suppression policies implemented in the mid-1800s and high-grading practices, which resulted in the loss of stand heterogeneity, limited forest regeneration, and the conversion of Scots pine (*Pinus sylvestris*) stands into Norway spruce (*Picea abies*). Burns initially targeted previously logged stands of *Pinus sylvestris*, whose failure to regenerate following logging was an increasing concern in forests across the country[^130,208^](https://www.zotero.org/google-docs/?VXqJ5F).

While mechanical soil preparation eventually replaced prescribed burning in the 1970s as the preferred method of site preparation, the general recognition of the need to reintroduce fire into the Swedish landscape to protect biodiversity in the boreal ecosystem remained: prescribed burning is still used by forestry companies for site preparation in conjunction with conservation purposes, with the Forest Stewardship Council requiring the application of prescribed burning to 5% of all annual clear-cut area on dry-mesic forest land for certification[^209^](https://www.zotero.org/google-docs/?bYuSQC); and in 2015, Life Taiga was established by the EU to conduct burns in protected areas across Sweden in order to increase structural habitat diversity. Conservation goals are generally centered around creating a heterogenous forest structure, increasing deadwood (upon which saproxylic insect species depend), and strengthening populations of fire-prone species[^130^](https://www.zotero.org/google-docs/?wd8epD).

Life Taiga burns were conducted in 14 provinces all over Sweden, with 74% (99 fires) occurring in the Boreal Forests/Taiga biome and the remainder occurring in the Temperate Broadleaf and Mixed Forests biome (24%, 35 fires). Across biomes, fires were predominantly conducted in needleleaf evergreen forests or mixed broadleaf and needleleaf evergreen forests (74%, 99 burns). Many of these burns target pine-dominated or mixed pine and spruce stands to maintain pine stand health or combat spruce encroachment, as this led to more open stands that better supported wildlife such as birds and insects[^208,210^](https://www.zotero.org/google-docs/?twPH04). Additional effort was made to establish dialogue with the Saami indigenous people, who practice reindeer husbandry. Fire, especially at low return intervals, can diminish reindeer lichen for decades, an important source of winter forage. Thus, it is important to consider this ecological effect[^207,210^](https://www.zotero.org/google-docs/?AqKIRn).

However, it should be noted that GlobalRx does not include burns conducted on commercial forest lands, which account for more area burned than that occurring only on natural reserves. Ref ([^211^](https://www.zotero.org/google-docs/?Rrd9om)), which examined data from 2011-2015, found that forestry companies were responsible for 85% of prescribed fires, covering 5280 Ha, nearly double that of the burned area of Life Taiga.

The burning season for Life Taiga spanned from May to mid-September, with 97% (129 fires) of fires occurring within that window. 81% (108 fires) of fires occur between May and July. This period corresponds with the snow free season and weather conditions in which the ground is dry enough to sustain fire[^207^](https://www.zotero.org/google-docs/?QMXAzS).

## Thailand

The use of fire for swidden agriculture and resource management dates back in Thailand for at least a millenia. Lowland and highland ethnic groups practiced different forms of swiddening on various forest types, resulting in secondary succession of varying distributions of grasses, shrubs and thorny species, and trees[^212^](https://www.zotero.org/google-docs/?n7M4ff). In addition to swiddening, fire is also used to manage food resources such as mushrooms and edible plants, hunting, the cultivation and management of important non-edible natural resources, such as grasses used for basketry and roofing, and to promote the germination of important timber species such as teak. Fire management, including the use of fire breaks, backfires, and fuel management have been a part of the traditional and cultural knowledge of the forest communities[^213^](https://www.zotero.org/google-docs/?1PT6XE). However, economic development and the introduction of commercial farming starting in the 1960s, combined with population growth and a ban on commercial timber harvesting starting in 1989, have resulted in a shift from subsistence practices to less sustainable practices, including decreasing fallow periods, increased deforestation for agricultural expansion, plantation monocultures, and poorly managed fire use[^212–214^](https://www.zotero.org/google-docs/?GrNhbz). Additionally, national forest and conservation policies, which stipulate state control over all forested lands and limit or even forbid occupation and use of forest resources, have displaced and marginalized many forest communities[^215^](https://www.zotero.org/google-docs/?3k1QlY). This has created land tenure conflicts and insecurities which further exacerbate forest fire activity as villagers no longer feel incentive to protect the land or combat wildfires[^213^](https://www.zotero.org/google-docs/?vXGnie).

Nearly all fires are started by people living near forested areas, primarily for agriculture, harvesting non-timber forest products, and hunting[^216^](https://www.zotero.org/google-docs/?BqQhFx). As such, the role of community participation in forest and fire management has been recognized internationally and nationally since the 1980s. The Community Forestry Act, initiated in 1990 by the Royal Forestry Department and finally enacted in 2019 after 30 years of revision, was the first attempt to federally recognize community forest rights[^217^](https://www.zotero.org/google-docs/?cJXr1J), and allows communities to develop their own fire protection and conservation plans in compliance with governmental regulations while still being able to use and benefit from forest resources[^213,217^](https://www.zotero.org/google-docs/?EUXUCV). Since 2019, over 11,000 community forests covering just over 1 million ha have been registered in over 17,000 villages all over the country, including but not limited to Chiang Mai, Lamphun, Mae Hong Son, Chasoengsao, and Ubon Ratchatani[^213,218^](https://www.zotero.org/google-docs/?KuQxgE).

All 174 of the burn records occur in the northeast of the country, where the majority of forest fire activity also occurs. This region is predominantly mixed deciduous, pine, and dry dipterocarp forest, a deciduous overstory and grasses that provide ample fuel to support surface fires. Many of the native Dipterocarpus tree and understory species found in these forests exhibit fire adaptations, including thick bark, hard seed coats, and resprouting capability. Fire also plays a key role in pine forest extent and in the regeneration of important timber species such as teak[^219,220^](https://www.zotero.org/google-docs/?BJmwWL). Fires typically occur during the long dry season, which generally occurs between January and April[^221,222^](https://www.zotero.org/google-docs/?jYujZo) and also overlaps with the period during which farmers commonly burn agricultural residue[^223,224^](https://www.zotero.org/google-docs/?J0cju5).

## United Kingdom

Fire in the United Kingdom is the most prominent in the English uplands, located primarily in the northern and western portions of Great Britain, where it has long been used to shape the landscape for up to thousands of years dating back to the Mesolithic era. The extent and prevalence of certain ecosystems, namely moorlands and heathlands dominated by heather (*Calluna vulgaris*), are thought to be anthropogenic, a result of centuries-long burning practices used to clear forests and woodlands. Historically, fire has been used to improve forage for sheep farming in peatlands and grasslands, while rotational burning in heathlands and moorlands has been used for the past 200 years to maintain the different successional stages of heather necessary to sustain red grouse populations for game hunting. Within forests and woodlands, fire management and research was primarily concerned with fire risk in thicket and pre-thicket conifer plantations in the 1960s-70s, until interest died off as plantations matured and timber value declined[^225^](https://www.zotero.org/google-docs/?H0CWVI).

Today, burning continues to be conducted primarily for sheep grazing and to maintain game populations of red grouse primarily in private estates[^226^](https://www.zotero.org/google-docs/?T7rrBC). Ref ([^225^](https://www.zotero.org/google-docs/?QRz3ei)) note that many experienced gamekeepers on private estates may have detailed and localized knowledge of intensely but well-managed heather moorlands, but these records are not in the public domain or recorded by agencies. Burning in national parks is also conducted to manage wildlife habitat and biodiversity[^227,228^](https://www.zotero.org/google-docs/?qxDz6A). Heather burning in the UK is subject to the Muirburn Code in Scotland and the Heather and Grass Burning Act in Wales and England. The legal burning season generally spans from October or November through March or April to protect wildlife during nesting season[^229^](https://www.zotero.org/google-docs/?ShWiZ4).

Burning for game management within national parks and protected areas has increased substantially in the last 50 years[^226,230^](https://www.zotero.org/google-docs/?crELzl), which have significant implications for water quality, biodiversity, and carbon storage[^231^](https://www.zotero.org/google-docs/?iFauwQ). Rotational burning of heather in moorlands is generally recommended in 10-25 year intervals to prevent the buildup of senescent vegetation, which can be woody and extremely flammable[^225,232^](https://www.zotero.org/google-docs/?mWuxOy). However, labor shortages, especially in Scotland, often lead to fire return intervals beyond this recommendation, thus increasing wildfire risk[^130,225^](https://www.zotero.org/google-docs/?B4HH5k). Contrarily, too-frequent burning in poorly managed sites may result in the loss of the shrub cover and invasion of grasses such as *Molinia caerulea*. Burning near and within peatlands and soils also raises concerns over carbon storage: losses may be significant if ignitions lead to peat smoldering. However, low intensity prescribed burns can be beneficial for *Sphagnum* moss, an important species for peat formation, and promote decomposition. Additionally, the passage of a more intense wildfire as a result of not burning may lead to erosion and more severe carbon storage loss, both through the direct combustion of peat and through changes to water table that affect the balance between aerobic and anaerobic decomposition[^225,233^](https://www.zotero.org/google-docs/?C4DDTk).

## United States

The highest concentration of prescribed burns occurred in the Southeastern US^^[[1]](#footnote-0)^^, in areas encompassed by the Atlantic Plain and southern portion of the Appalachian Highlands. This region is predominantly of the Temperate Broadleaf & Mixed Forests or Temperate Conifer Forests biomes. Presently, the majority of wildlands in the Southeast are privately owned, and thus the use of prescribed fire is readily facilitated by the presence of prescribed burning associations (PBAs). Additionally, state-level legislation such as the 1990 Prescribed Burning Act in Florida protects landowners’ right to conduct prescribed burns by mitigating concerns about liability, which is commonly noted as the top barrier to conducting burns[^234,235^](https://www.zotero.org/google-docs/?pt5Jw2).

Prescribed burning was generally more culturally accepted in the southeast relative to other parts of the US, as the region was largely colonized by immigrants from rural western England, Scotland, and Ireland, where prescribed burning for improving forage in open range herding was already commonplace[^236^](https://www.zotero.org/google-docs/?sLIIsf). Thus, woodland burning was commonly used by cattle breeders and farmers in the early 1900s, which helped maintain the high-frequency (2-3 year fire return interval) fire regime that had previously been maintained by the native peoples prior to their decimation by settlers[^237–240^](https://www.zotero.org/google-docs/?G68cVf). However, between the mid-19th and 20th centuries, lumber companies, foresters, and government agencies turned public sentiment against fire use, initiating a massive public campaign through the American Forestry Association and Forest Service against controlled burning. These efforts were fueled in part by “research efforts” whose conclusions perpetuated classist and racist myths that motives for fire use were for “boredom and ritualistic tradition”[^236^](https://www.zotero.org/google-docs/?9pxh5n).

Prescribed burning was first reintroduced to the region in an effort to increase bobwhite quail populations, an important game species[^236,241^](https://www.zotero.org/google-docs/?IgZnm2). The vast majority of Southeast burns were conducted in either needleleaf evergreen or broadleaf forests, or a mixture thereof. Prescribed burning is commonly used in conservation efforts in longleaf pine (Pinus palustris) ecosystems found in the South Atlantic coastal areas, which include Georgia, Florida, and Alabama, the lowlands of Mississippi and Louisiana, and Texas. In the 19th and 20th centuries, these regions were subjected to fire suppression, timber overharvesting, agricultural expansion, and increasing invasive hog populations, resulting in the loss of longleaf pine ecosystems and a decline in native biodiversity[^242^](https://www.zotero.org/google-docs/?leGAmr). However, prescribed fire has been shown to benefit these ecosystems and has been used to restore habitat of keystone species such as the gopher tortoise[^243,244^](https://www.zotero.org/google-docs/?tXsoAj). 6.2 million hectares of longleaf pine sites, located in “Significant Geographic Areas (SGA)” encompassing protected areas, were burned from 2011-2021 by members of the Longleaf Partnership Council. Hazardous fuel reduction is sometimes an objective of these burns[^245,246^](https://www.zotero.org/google-docs/?lf1HOm).

In the Central US Great Plains^^[[2]](#footnote-1)^^ region, grasslands have been the dominant vegetation for the last 5000-8000 years, with the prevalence of woody plants, particularly Ashe and Virginian juniper (Juniperus ashei, Juniperus virginiana), being closely tied with anthropogenic fire and burning by Plains Indians to facilitate movement and buffalo hunting[^247^](https://www.zotero.org/google-docs/?pr2fyi). However, the forcible displacement of Plains Indians, fragmentation of the landscape for settlement and agriculture, overgrazing from the overstocking of domestic livestock, federal fire suppression policies, and human-mediated dispersal and planting of juniper trees from the 1850s-1930s led to widespread fire exclusion that resulted in radical losses of grasslands[^248,249^](https://www.zotero.org/google-docs/?muJPBW). In the 1990s, grassroots movements to address the degradation of grasslands and the potential of prescribed fire to restore them led to the formation of the first prescribed burning associations (PBAs). PBAs consist of groups of private landowners and other interested people who form partnerships to pool their knowledge, equipment, and other resources to conduct prescribed fires[^250^](https://www.zotero.org/google-docs/?FBC11f).

PBAs are now commonplace in the Southeast and Great Plains regions, with over 100 across 18 states as of 2022, the majority of which are concentrated in the Plains region[^251^](https://www.zotero.org/google-docs/?3dymkm). PBAs help to facilitate prescribed burns, particularly on private land, by providing training, resources, and even liability insurance for burns. In the Plains region, killing or preventing juniper encroachment was the most important objective, though burns were also commonly conducted for livestock production, wildlife management, rangeland maintenance[^252^](https://www.zotero.org/google-docs/?d4rpqY).

Much of the western US is characterized by low elevation mixed conifer forests that historically experienced a frequent, low-intensity fire regime that was maintained by ignitions from both Native Americans and lightning. However, colonization and the introduction of commercial logging resulted in widespread fire exclusion in these forests, first from removal and displacement of the Native peoples, and then through the implementation of stringent fire suppression policies. The result was the massive accumulation of surface and ladder fuel loads and increases in forest density and fuel continuity that today enable fires to burn more extensively and at a higher severity relative to the pre-settlement period[^253–255^](https://www.zotero.org/google-docs/?p5ibm3).

Prescribed burning was introduced to these forests beginning in the 1960s, originally under the premise of ecological restoration in Giant Sequoia forests[^256,257^](https://www.zotero.org/google-docs/?86iM5a). Today, prescribed burns are primarily conducted for hazardous fuel reduction and ecological restoration, especially in areas near the WUI[^258–260^](https://www.zotero.org/google-docs/?LIbq3C). Burns typically target ladder and surface fuels, with the goal of decreasing the severity of a subsequent wildfire[^260,261^](https://www.zotero.org/google-docs/?jVdHb5). Burns are typically conducted from the fall through the winter and into early spring (September-May), in months outside of the typical wildfire season (June-August)[^262^](https://www.zotero.org/google-docs/?YJJlpl).

Some prescribed burns in the Western US occur in Deserts and Xeric Shrublands, predominantly in pinyon-juniper (P-J) woodlands. P-J woodland ecosystems can form a range of different tree, shrub, and grass complexes, from savanna grassland types to closed canopy forests[^263^](https://www.zotero.org/google-docs/?woWh0k). Dramatic increases in tree density have been observed across all region types in the past century, possibly due to fire suppression[^264^](https://www.zotero.org/google-docs/?KRLrep). Burns were conducted primarily for hazardous fuel reduction in shrubland and forest types, which have moderately dense canopy cover[^265^](https://www.zotero.org/google-docs/?GvBi70). In P-J shrubland and grassland types, prescribed fire may also restore understory communities of shrubs, grasses, and forbs by reducing tree competition[^266^](https://www.zotero.org/google-docs/?dQMzFS).

85% of all prescribed burns in the US are conducted at the state or local levels, and many efforts in the Southeast, Midwest, and increasingly the West are supported by prescribed burning associations (PBAs) or prescribed burning councils (PBCs). The objectives of PBAs and PBCs differ by state, but they generally provide a platform by which landowners can obtain training, pool their knowledge and equipment, and also obtain hands-on training on the use of prescribed fire[^250^](https://www.zotero.org/google-docs/?8LKSsD). PBA support can greatly reduce social and financial barriers to conducting prescribed burns, including liability and staffing[^267^](https://www.zotero.org/google-docs/?ZqLlmT).

Like many regions of North America, fire was used by certain Indigenous groups across Alaska for thousands of years, particularly in more fire-prone areas such as the boreal forests in Gwich’in territory in the northeast of the state. Fire was used for clearing underbrush to facilitate movement and hunting, to influence the movement of game such as moose, to kill standing timber for caribou fencing, and to combat pests such as mosquitoes[^268^](https://www.zotero.org/google-docs/?alTBD7). Much of Interior Alaska, which is dominated by boreal forests of spruce (black spruce, *Picea mariana*; white spruce, *Picea glauca*) and hardwood mixes (poplar, *Populus balsamifera*; birch, *Betula neoalaskana*; and aspen, *Populus tremuloides*), has experienced a mixed-severity fire regime, with fires that are carried by ground fuels but frequently move into the canopy[^269^](https://www.zotero.org/google-docs/?CMUR95). Black spruce, which has resinous needles and low hanging branches, is particularly prone to crown fire[^270^](https://www.zotero.org/google-docs/?iCV4fF). Fire in Alaska is largely governed by climate and teleconnections such as the Pacific Decadal Oscillation, and is especially sensitive to temperature[^271^](https://www.zotero.org/google-docs/?VkaN80). However, like other regions of the US, the fire regime has also been affected by colonization: settlers decimated the Indigenous population, altering the extent of Indigenous burning practices particularly in the east, while influxes of white miners and settlers during the gold rushes of the 1890s drastically increased the incidence of fires[^272,273^](https://www.zotero.org/google-docs/?tNOauV). Additionally, the construction and operation of the Alaska Railroad beginning in 1915 also increased the incidence of fires[^272^](https://www.zotero.org/google-docs/?8ingex)).

Fire suppression beginning in the 1960s has also had a marked effect on Alaska’s fire regime, though this effect is primarily concentrated in regions designated in the 1980s as “active suppression zones,” where damage to life and property are greatest. Active suppression zones cover approximately 17% of the Interior Alaska, while nearly ⅔ of this region is allowed a “natural” fire regime, wherein fires are monitored but generally allowed to burn[^273^](https://www.zotero.org/google-docs/?QSNrQv). The role of fire in Alaska’ ecosystems was first recognized in the 1950s for the management of moose browse, and has since been recognized for its importance the management of other animal species’ habitats, maintaining early successional plant communities, nutrient cycling, and mosaics that support plant and animal diversity[^272^](https://www.zotero.org/google-docs/?vi7XA8). Prescribed burning has been used for a combination of hazardous fuel reduction and habitat management for moose and grouse, especially in mixed broadleaf forests[^274,275^](https://www.zotero.org/google-docs/?GJ2Z7M). Many hazardous fuel reduction burns, often stand-replacing, are focused on constructing fuel breaks and mitigating fire risk among beetle-killed trees in spruce forests[^274,276,277^](https://www.zotero.org/google-docs/?XeDuJE). However, ref ([^278^](https://www.zotero.org/google-docs/?o5lJOg)) also notes that federal prescribed burning policies, which rely on generalized narratives on fire suppression and fuel accumulation developed predominantly in the Western US, may be inappropriate for large remote parts of Alaska that were relatively unaffected by fire suppression and also experience a historically different fire regime. Additionally, these policies, which often advocate for low to moderate severity burns in regions that have not historically burned in that manner, may adversely affect local ecology. Thus, it is important to consider local, place-based knowledge and the complexities of different ecosystems[^278^](https://www.zotero.org/google-docs/?SybeQ8).

# Bibliography

[1. NPWS Fire History - Wildfires and Prescribed Burns - SEED.](https://www.zotero.org/google-docs/?Tq4xaF)

[2. Darwin Centre of Bushfire Research. Northern Australian Fire Information (NAFI) Prescribed Fire Lines. Dataset accessed upon request [Jay Evans, pers. comm.] (2021).](https://www.zotero.org/google-docs/?Tq4xaF)

[3. Queensland Parks and Wildlife Service. Fire history - Queensland Parks and Wildlife Service. corporateName=The State of Queensland; jurisdiction=Queensland.](https://www.zotero.org/google-docs/?Tq4xaF)

[4. South Australia Department for Environment and Water. Bushfires and Prescribed Burns History.](https://www.zotero.org/google-docs/?Tq4xaF)

[5. Department of Natural Resources and Environment Tasmania. Fire History.](https://www.zotero.org/google-docs/?Tq4xaF)

[6. Department of Energy, Environment and Climate Action. Fire History Records of Fires across Victoria.](https://www.zotero.org/google-docs/?Tq4xaF)

[7. Department of Biodiversity, C. and A. DBCA Fire History (DBCA-060).](https://www.zotero.org/google-docs/?Tq4xaF)

[8. IBAMA/PREVFOGO ((Brazilian Institute of the Environment and Renewable Natural Resources/National Center to Prevent and Combat Forest Fires). Record of prescribed burns on public land. Dataset accessed upon request [Lara Steil, Ricardo Barreto, Rodrigo Falleiro and Rosa Maria Cuesta, pers. comm. (2021).](https://www.zotero.org/google-docs/?Tq4xaF)

[9. Parks Canada. Record of prescribed burns in Banff National Park. Dataset accessed upon request [Jane Park, pers. comm.] (2020).](https://www.zotero.org/google-docs/?Tq4xaF)

[10. Natural Resources Canada. Canadian Wildland Fire Information System | Download data.](https://www.zotero.org/google-docs/?Tq4xaF)

[11. Parks Canada. Record of prescribed burns on national park land. Dataset accessed upon request [Emma Zerr, pers. comm.] (2020).](https://www.zotero.org/google-docs/?Tq4xaF)

[12. East-Pyrenees Prescribed Burn Team & National Institute of Agronomic Research (INRAE). Record of prescribed burns on public land. Dataset accessed upon request [Eric Rigolot, pers. comm.] (2021).](https://www.zotero.org/google-docs/?Tq4xaF)

[13. German Federal Real Estate Administration. Record of prescribed burns on public land. Dataset accessed upon request [Gernot Rucker, pers. comm.] (2021).](https://www.zotero.org/google-docs/?Tq4xaF)

[14. Italian Society of Silviculture and Forest Ecology, Fire Management. Record of prescribed burns on public land. Dataset accessed upon request [David Ascoli, pers. comm.] (2021).](https://www.zotero.org/google-docs/?Tq4xaF)

[15. Yamashita, Y. & Hokkaido University. Record of prescribed burns on public land from regional authorities. Dataset accessed upon request [Youhei Yamashita pers. comm.] (2021).](https://www.zotero.org/google-docs/?Tq4xaF)

[16. National Forestry Commission (CONAFOR). Records from the federal prescribed burn program. [Accessed upon request by Cesar A. Robles-Gutierrez, pers. comm.].](https://www.zotero.org/google-docs/?Tq4xaF)

[17. Institute for Nature Conservation and Forests (ICNF). Record of prescribed burns on public land. Dataset accessed upon request [Paulo Fernandes, pers. comm.] (2023).](https://www.zotero.org/google-docs/?Tq4xaF)

[18. Wildfires Monitoring Information System of the Federal Forestry Agency (ISDM-Rosleskhoz). Record of prescribed burns in Russia. Dataset accessed upon request [Elena Kukavskaya, pers. comm.] (2021).](https://www.zotero.org/google-docs/?Tq4xaF)

[19. South Africa National Parks (SANParks). Record of prescribed burns in Kruger National Park. Dataset accessed upon request [Tercia Strydom, pers. comm.] (2021).](https://www.zotero.org/google-docs/?Tq4xaF)

[20. South Africa National Parks (SANParks). Record of prescribed burns in Garden Route National Park. Dataset accessed upon request [Johan Baard, pers. comm.] (2021).](https://www.zotero.org/google-docs/?Tq4xaF)

[21. South Africa National Parks (SANParks). Record of prescribed burns in Table Mountain National Park. Dataset accessed upon request [Chad Cheney, pers. comm.] (2021).](https://www.zotero.org/google-docs/?Tq4xaF)

[22. Government of Andalucia. Record of prescribed burns on public land. Dataset accessed upon request [F. Senra-Rivero, H. Alfaro-Fernandez and J. Becerra, pers. comm.] (2021).](https://www.zotero.org/google-docs/?Tq4xaF)

[23. Principality of Asturias. Record of prescribed burns on public land. Dataset accessed upon request [A. Lopez-Valverde, pers. comm.] (2021).](https://www.zotero.org/google-docs/?Tq4xaF)

[24. Government of Galicia. Record of prescribed burns on public land. Dataset accessed upon request [Servizo de Coordinacion de Medios, Direccion Xeral de Defensa do Monte, pers. comm.] (2021).](https://www.zotero.org/google-docs/?Tq4xaF)

[25. Generalitat de Catalunya. Cremes prescrites executades per bombers. *Departament d’Interior* http://interior.gencat.cat/ca/serveis/informacio-geografica/bases-cartografiques/cremes-prescrites-dels-bombers/ (2021).](https://www.zotero.org/google-docs/?Tq4xaF)

[26. LifeTaiga Project. Record of prescribed burns during the LifeTaiga Science Project (EU Commission). Dataset accessed upon request [Niclas Bergius, Julia Carlsson, pers. comm.] (2021).](https://www.zotero.org/google-docs/?Tq4xaF)

[27. Thailand Ministry of Natural Resources and Environment, Department of National Parks, Wildlife and Plants Conservation (Forest Fire Control Division). Record of prescribed burns on national park land. Dataset accessed upon request [Veerachai Tanpipat, pers. comm.] (2022).](https://www.zotero.org/google-docs/?Tq4xaF)

[28. Forestry England. Record of prescribed burns in New Forest National Park. Dataset accessed upon request [Lisa Macher, pers. comm.] (2021).](https://www.zotero.org/google-docs/?Tq4xaF)

[29. Department of the Interior & Forest Service. Monitoring Trends in Burn Severity.](https://www.zotero.org/google-docs/?Tq4xaF)

[30. Forest Service & US Department of Agriculture. Fire and tree mortality database (FTM) (2nd Edition).](https://www.zotero.org/google-docs/?Tq4xaF)

[31. IFTDSS. https://iftdss.firenet.gov/landing_page/.](https://www.zotero.org/google-docs/?Tq4xaF)

[32. Welty, J. & Jeffries, M. Combined wildland fire datasets for the United States and certain territories, 1800s-Present. U.S. Geological Survey https://doi.org/10.5066/P9ZXGFY3 (2021).](https://www.zotero.org/google-docs/?Tq4xaF)

[33. Swain, D. L. *et al.* Climate change is narrowing and shifting prescribed fire windows in western United States. *Commun Earth Environ* **4**, 1–14 (2023).](https://www.zotero.org/google-docs/?Tq4xaF)

[34. Raumann, C. G. & Soulard, C. E. Sierra Nevada Ecoregion. in *Status and Trends of Land Change in the Western United STates - 1973-2000* (2012).](https://www.zotero.org/google-docs/?Tq4xaF)

[35. Griffith, G. E. *et al. Ecoregions of California*. *Open-File Report* https://pubs.usgs.gov/publication/ofr20161021 (2016) doi:10.3133/ofr20161021.](https://www.zotero.org/google-docs/?Tq4xaF)

[36. Baijnath-Rodino, J. A. *et al.* Historical seasonal changes in prescribed burn windows in California. *Science of The Total Environment* **836**, 155723 (2022).](https://www.zotero.org/google-docs/?Tq4xaF)

[37. Quinn-Davidson, L. & Stackhouse, J. Prescribed Fire Liability in California.](https://www.zotero.org/google-docs/?Tq4xaF)

[38. Morgan, G. W. *et al.* Prescribed burning in south-eastern Australia: history and future directions. *Australian Forestry* **83**, 4–28 (2020).](https://www.zotero.org/google-docs/?Tq4xaF)

[39. Australian Bureau Of Agricultural Resource Economics And Sciences (ABARES). Australian forest profiles 2019: Eucalypt. Preprint at https://doi.org/10.25814/5D9167D34241F (2019).](https://www.zotero.org/google-docs/?Tq4xaF)

[40. Attiwill, P. M. Ecological disturbance and the conservative management of eucalypt forests in Australia. *Forest Ecology and Management* **63**, 301–346 (1994).](https://www.zotero.org/google-docs/?Tq4xaF)

[41. McKemey, M., Ens, E., Rangers, Y. M., Costello, O. & Reid, N. Indigenous Knowledge and Seasonal Calendar Inform Adaptive Savanna Burning in Northern Australia. *Sustainability* **12**, 995 (2020).](https://www.zotero.org/google-docs/?Tq4xaF)

[42. Altangerel, K. & Kull, C. A. The prescribed burning debate in Australia: conflicts and compatibilities. *Journal of Environmental Planning and Management* (2013).](https://www.zotero.org/google-docs/?Tq4xaF)

[43. Gott, B. Aboriginal fire management in south-eastern Australia: aims and frequency. *Journal of Biogeography* **32**, 1203–1208 (2005).](https://www.zotero.org/google-docs/?Tq4xaF)

[44. McArthur, A. G. (Alan G. *Fire Behaviour in Eucalypt Forests / A.G. McArthur.* (Australia Forestry and Timber Bureau, 1967).](https://www.zotero.org/google-docs/?Tq4xaF)

[45. NSW Sate of the Environment. *NSW State of the Environment 2021*. https://www.soe.epa.nsw.gov.au/about-the-report/how-to-use-this-report (2021).](https://www.zotero.org/google-docs/?Tq4xaF)

[46. State of Victoria Department of Environment, Land, Water and Planning. *Code of Practice for Bushfire Management on Public Land*. https://www.ffm.vic.gov.au/__data/assets/pdf_file/0006/21300/Code-of-Practice-for-Bushfire-Management-on-Public-Land-2012-amended-2022.pdf (2022).](https://www.zotero.org/google-docs/?Tq4xaF)

[47. State Government of Victoria. Strategic Bushfire Management Planning. *Safer Together* https://www.safertogether.vic.gov.au/strategic-bushfire-management-planning (2024).](https://www.zotero.org/google-docs/?Tq4xaF)

[48. Forestry Australia. PRESCRIBED BURNING. *Forestry Australia* https://www.forestry.org.au/prescribed-burning/.](https://www.zotero.org/google-docs/?Tq4xaF)

[49. Burrows, N. & McCaw, L. Prescribed burning in southwestern Australian forests. *Frontiers in Ecology and the Environment* **11**, e25–e34 (2013).](https://www.zotero.org/google-docs/?Tq4xaF)

[50. Abbott, I. Aboriginal fire regimes in south-west Western Australia: evidence from historical documents. in *Fire in ecosystems of south-west Western Australia: impacts and management* 119–146 (Backhuys Publishers, 2003).](https://www.zotero.org/google-docs/?Tq4xaF)

[51. Prober, S. M., Yuen, E., O’Connor, M. H. & Schultz, L. Ngadju kala: Australian Aboriginal fire knowledge in the Great Western Woodlands. *Austral Ecology* **41**, 716–732 (2016).](https://www.zotero.org/google-docs/?Tq4xaF)

[52. Bliege Bird, R., Bird, D. W., Codding, B. F., Parker, C. H. & Jones, J. H. The “fire stick farming” hypothesis: Australian Aboriginal foraging strategies, biodiversity, and anthropogenic fire mosaics. *Proceedings of the National Academy of Sciences* **105**, 14796–14801 (2008).](https://www.zotero.org/google-docs/?Tq4xaF)

[53. Burrows, N., Burbidge, A., Fuller, P. J. & Behn, G. Evidence of altered fire regimes in the Western Desert regime of Australia. *Conservation Science Western Australia* **5**, 272–284 (2006).](https://www.zotero.org/google-docs/?Tq4xaF)

[54. Bird, D. W., Bird, R. B., Codding, B. F. & Taylor, N. A Landscape Architecture of Fire: Cultural Emergence and Ecological Pyrodiversity in Australia’s Western Desert. *Current Anthropology* **57**, S65–S79 (2016).](https://www.zotero.org/google-docs/?Tq4xaF)

[55. Howard, T., Burrows, N., Smith, T., Daniel, G. & McCaw, L. A framework for prioritising prescribed burning on public land in Western Australia. *Int. J. Wildland Fire* **29**, 314–325 (2020).](https://www.zotero.org/google-docs/?Tq4xaF)

[56. Department of Biodiversity, Conservation and Attractions. DBCA Fire Management Strategy. https://www.dbca.wa.gov.au/management/fire (2023).](https://www.zotero.org/google-docs/?Tq4xaF)

[57. Burrows, N., Ward, B. & Robinson, A. Fire behaviour in spinifex fuels on the Gibson Desert Nature Reserve, Western Australia. *Journal of Arid Environments* **20**, 189–204 (1991).](https://www.zotero.org/google-docs/?Tq4xaF)

[58. Russell-Smith, J. & Bowman, D. M. J. S. Conservation of monsoon rainforest isolates in the Northern Territory, Australia. *Biological Conservation* **59**, 51–63 (1992).](https://www.zotero.org/google-docs/?Tq4xaF)

[59. Lewis, H. T. Ecological and Technological Knowledge of Fire: Aborigines Versus Park Rangers in Northern Australia. *American Anthropologist* **91**, 940–961 (1989).](https://www.zotero.org/google-docs/?Tq4xaF)

[60. Yibarbuk, D. *et al.* Fire ecology and Aboriginal land management in central Arnhem Land, northern Australia: a tradition of ecosystem management. *Journal of Biogeography* **28**, 325–343 (2001).](https://www.zotero.org/google-docs/?Tq4xaF)

[61. Northern Territory Government. Fire management projects. https://depws.nt.gov.au/bushfire-information-and-management/aboriginal-carbon-industry-strategy (2023).](https://www.zotero.org/google-docs/?Tq4xaF)

[62. Northern Territory Department of Environment and Natural Resources. Aboriginal Carbon Industry Strategy.](https://www.zotero.org/google-docs/?Tq4xaF)

[63. Ramos-Neto, M. B. & Pivello, V. R. Lightning Fires in a Brazilian Savanna National Park: Rethinking Management Strategies. *Environmental Management* **26**, 675–684 (2000).](https://www.zotero.org/google-docs/?Tq4xaF)

[64. Mistry, J. Fire in the cerrado (savannas) of Brazil: an ecological review. *Progress in Physical Geography: Earth and Environment* **22**, 425–448 (1998).](https://www.zotero.org/google-docs/?Tq4xaF)

[65. Simon, M. F. *et al.* Recent assembly of the Cerrado, a neotropical plant diversity hotspot, by in situ evolution of adaptations to fire. *Proceedings of the National Academy of Sciences* **106**, 20359–20364 (2009).](https://www.zotero.org/google-docs/?Tq4xaF)

[66. Coutinho, L. M. Ecological Effects of Fire in Brazilian Cerrado. in *Ecology of Tropical Savannas* (eds. Huntley, B. J. & Walker, B. H.) 273–291 (Springer, Berlin, Heidelberg, 1982). doi:10.1007/978-3-642-68786-0_13.](https://www.zotero.org/google-docs/?Tq4xaF)

[67. Coutinho, L. M. Fire in the Ecology of the Brazilian Cerrado. in *Fire in the Tropical Biota: Ecosystem Processes and Global Challenges* (ed. Goldammer, J. G.) 82–105 (Springer, Berlin, Heidelberg, 1990). doi:10.1007/978-3-642-75395-4_6.](https://www.zotero.org/google-docs/?Tq4xaF)

[68. Pivello, V. R. The Use of Fire in the Cerrado and Amazonian Rainforests of Brazil: Past and Present. *fire ecol* **7**, 24–39 (2011).](https://www.zotero.org/google-docs/?Tq4xaF)

[69. Uhl, C. & Kauffman, J. B. Deforestation, Fire Susceptibility, and Potential Tree Responses to Fire in the Eastern Amazon. *Ecology* **71**, 437–449 (1990).](https://www.zotero.org/google-docs/?Tq4xaF)

[70. Cochrane, M. A. *et al.* Positive Feedbacks in the Fire Dynamic of Closed Canopy Tropical Forests. *Science* **284**, 1832–1835 (1999).](https://www.zotero.org/google-docs/?Tq4xaF)

[71. Sanford, R. L., Saldarriaga, J., Clark, K. E., Uhl, C. & Herrera, R. Amazon Rain-Forest Fires. *Science* **227**, 53–55 (1985).](https://www.zotero.org/google-docs/?Tq4xaF)

[72. Balch, J. K., Nepstad, D. C. & Curran, L. M. Pattern and process: Fire-initiated grass invasion at Amazon transitional forest edges. in *Tropical Fire Ecology: Climate Change, Land Use, and Ecosystem Dynamics* (ed. Cochrane, M. A.) 481–502 (Springer, Berlin, Heidelberg, 2009). doi:10.1007/978-3-540-77381-8_17.](https://www.zotero.org/google-docs/?Tq4xaF)

[73. Welch, J. R. & Coimbra Jr., C. E. A. Indigenous fire ecologies, restoration, and territorial sovereignty in the Brazilian Cerrado: The case of two Xavante reserves. *Land Use Policy* **104**, 104055 (2021).](https://www.zotero.org/google-docs/?Tq4xaF)

[74. Mistry, J. *et al.* Indigenous Fire Management in the cerrado of Brazil: The Case of the Krahô of Tocantíns. *Human Ecology* **33**, 365–386 (2005).](https://www.zotero.org/google-docs/?Tq4xaF)

[75. Padoch, C. & Pinedo-Vasquez, M. Saving Slash-and-Burn to Save Biodiversity. *Biotropica* **42**, 550–552 (2010).](https://www.zotero.org/google-docs/?Tq4xaF)

[76. Thrupp, L. A., Hecht, S. & Browder, J. O. *The Diversity and Dynamics of Shifting Cultivation: Myths, Realities, and Policy Implications*. (World Resources Institute, Washington, DC, 1997).](https://www.zotero.org/google-docs/?Tq4xaF)

[77. Aragão, L. E. O. C. & Shimabukuro, Y. E. The Incidence of Fire in Amazonian Forests with Implications for REDD. *Science* **328**, 1275–1278 (2010).](https://www.zotero.org/google-docs/?Tq4xaF)

[78. Aragão, L. E. O. C. *et al.* Interactions between rainfall, deforestation and fires during recent years in the Brazilian Amazonia. *Philosophical Transactions of the Royal Society B: Biological Sciences* **363**, 1779–1785 (2008).](https://www.zotero.org/google-docs/?Tq4xaF)

[79. Cano-Crespo, A., Oliveira, P. J. C., Boit, A., Cardoso, M. & Thonicke, K. Forest edge burning in the Brazilian Amazon promoted by escaping fires from managed pastures. *Journal of Geophysical Research: Biogeosciences* **120**, 2095–2107 (2015).](https://www.zotero.org/google-docs/?Tq4xaF)

[80. Scaramuzza, C. A. de M. *et al.* LAND-USE AND LAND-COVER MAPPING OF THE BRAZILIAN CERRADO BASED MAINLY ON LANDSAT-8 SATELLITE IMAGES. *Revista Brasileira de Cartografia* **69**, (2017).](https://www.zotero.org/google-docs/?Tq4xaF)

[81. Garrett, R. D. *et al.* Forests and Sustainable Development in the Brazilian Amazon: History, Trends, and Future Prospects. *Annual Review of Environment and Resources* **46**, 625–652 (2021).](https://www.zotero.org/google-docs/?Tq4xaF)

[82. Garcia, A. S. & Ballester, M. V. R. Land cover and land use changes in a Brazilian Cerrado landscape: drivers, processes, and patterns. *Journal of Land Use Science* **11**, 538–559 (2016).](https://www.zotero.org/google-docs/?Tq4xaF)

[83. Schmidt, I. B. & Eloy, L. Fire regime in the Brazilian Savanna: Recent changes, policy and management. *Flora* **268**, 151613 (2020).](https://www.zotero.org/google-docs/?Tq4xaF)

[84. Eloy, L., Aubertin, C., Toni, F., Lúcio, S. L. B. & Bosgiraud, M. On the margins of soy farms: traditional populations and selective environmental policies in the Brazilian Cerrado. *The Journal of Peasant Studies* **43**, 494–516 (2016).](https://www.zotero.org/google-docs/?Tq4xaF)

[85. Fearnside, P. M. Deforestation in Brazilian Amazonia: History, Rates, and Consequences. *Conservation Biology* **19**, 680–688 (2005).](https://www.zotero.org/google-docs/?Tq4xaF)

[86. Kirby, K. R. *et al.* The future of deforestation in the Brazilian Amazon. *Futures* **38**, 432–453 (2006).](https://www.zotero.org/google-docs/?Tq4xaF)

[87. Urzedo, D. & Chatterjee, P. The Colonial Reproduction of Deforestation in the Brazilian Amazon: Violence Against Indigenous Peoples for Land Development. *Journal of Genocide Research* **23**, 302–324 (2021).](https://www.zotero.org/google-docs/?Tq4xaF)

[88. Conceição, K. V. *et al.* Government policies endanger the indigenous peoples of the Brazilian Amazon. *Land Use Policy* **108**, 105663 (2021).](https://www.zotero.org/google-docs/?Tq4xaF)

[89. Begotti, R. A. & Peres, C. A. Rapidly escalating threats to the biodiversity and ethnocultural capital of Brazilian Indigenous Lands. *Land Use Policy* **96**, 104694 (2020).](https://www.zotero.org/google-docs/?Tq4xaF)

[90. Carmenta, R., Vermeylen, S., Parry, L. & Barlow, J. Shifting Cultivation and Fire Policy: Insights from the Brazilian Amazon. *Hum Ecol* **41**, 603–614 (2013).](https://www.zotero.org/google-docs/?Tq4xaF)

[91. Carmenta, R., Zabala, A., Daeli, W. & Phelps, J. Perceptions across scales of governance and the Indonesian peatland fires. *Global Environmental Change* **46**, 50–59 (2017).](https://www.zotero.org/google-docs/?Tq4xaF)

[92. Bilbao, B., Mistry, J., Millán, A. & Berardi, A. Sharing Multiple Perspectives on Burning: Towards a Participatory and Intercultural Fire Management Policy in Venezuela, Brazil, and Guyana. *Fire* **2**, 39 (2019).](https://www.zotero.org/google-docs/?Tq4xaF)

[93. Eloy, L., A. Bilbao, B., Mistry, J. & Schmidt, I. B. From fire suppression to fire management: Advances and resistances to changes in fire policy in the savannas of Brazil and Venezuela. *The Geographical Journal* **185**, 10–22 (2019).](https://www.zotero.org/google-docs/?Tq4xaF)

[94. Schmidt, I. B. *et al.* Fire management in the Brazilian savanna: First steps and the way forward. *Journal of Applied Ecology* **55**, 2094–2101 (2018).](https://www.zotero.org/google-docs/?Tq4xaF)

[95. Aldrich, S., Walker, R., Simmons, C., Caldas, M. & Perz, S. Contentious Land Change in the Amazon’s Arc of Deforestation. *Annals of the Association of American Geographers* **102**, 103–128 (2012).](https://www.zotero.org/google-docs/?Tq4xaF)

[96. Rajão, R. & Vurdubakis, T. On the Pragmatics of Inscription: Detecting Deforestation in the Brazilian Amazon. *Theory, Culture & Society* **30**, 151–177 (2013).](https://www.zotero.org/google-docs/?Tq4xaF)

[97. Eloy, L., Schmidt, I. B., Borges, S. L., Ferreira, M. C. & dos Santos, T. A. Seasonal fire management by traditional cattle ranchers prevents the spread of wildfire in the Brazilian Cerrado. *Ambio* **48**, 890–899 (2019).](https://www.zotero.org/google-docs/?Tq4xaF)

[98. Parks Canada Agency, G. of C. Prescribed fire – Parks Canada - Prescribed fire. https://parks.canada.ca/nature/science/conservation/feu-fire/dirige-prescribed (2024).](https://www.zotero.org/google-docs/?Tq4xaF)

[99. ontario.ca. Prescribed burns | ontario.ca. http://www.ontario.ca/page/prescribed-burn (2024).](https://www.zotero.org/google-docs/?Tq4xaF)

[100. alberta.ca. Prescribed fire | Alberta.ca. https://www.alberta.ca/prescribed-fire (2024).](https://www.zotero.org/google-docs/?Tq4xaF)

[101. gov.bc.ca. Search Results for “prescribed fire” – BC Wildfire Service. https://blog.gov.bc.ca/bcwildfire/?s=prescribed+fire (2024).](https://www.zotero.org/google-docs/?Tq4xaF)

[102. Weber, M. G. & Taylor, S. W. The use of prescribed fire in the management of Canada’s forested lands. *THE FORESTRY CHRONICLE* **68**, 11 (1992).](https://www.zotero.org/google-docs/?Tq4xaF)

[103. Faivre, N. *et al.* Prescribed burning of harvested boreal black spruce forests in eastern Canada: effect on understory vegetation. *Can. J. For. Res.* **46**, 876–884 (2016).](https://www.zotero.org/google-docs/?Tq4xaF)

[104. Arno, S., Parsons, D. & Keane, R. Mixed-severity fire regimes in the northern Rocky Mountains: consequences of fire exclusion and options for the future. (2000).](https://www.zotero.org/google-docs/?Tq4xaF)

[105. Barrett, S. W. & Arno, S. F. Indian Fires in the Northern Rockies. 15.](https://www.zotero.org/google-docs/?Tq4xaF)

[106. Smith, C. M. *et al.* Whitebark pine and white pine blister rust in the Rocky Mountains of Canada and northern Montana. *Can. J. For. Res.* **38**, 982–995 (2008).](https://www.zotero.org/google-docs/?Tq4xaF)

[107. Wilson, B., Jouvet, S., Stuart-Smith, J. & Walker, R. Forest Structure Twenty Years After the First Whitebark Pine Prescribed Burn in Banff National Park. *Research and Management of High-Elevation Five-Needle Pines in Western North America* (2022).](https://www.zotero.org/google-docs/?Tq4xaF)

[108. Sachro, L. L., Strong, W. L. & Gates, C. C. Prescribed burning effects on summer elk forage availability in the subalpine zone, Banff National Park, Canada. *Journal of Environmental Management* **77**, 183–193 (2005).](https://www.zotero.org/google-docs/?Tq4xaF)

[109. Hirsch, K. & Pengelly, I. *Fuel Reduction in Lodgepole Pine Stands in Banff National Park*. (2000).](https://www.zotero.org/google-docs/?Tq4xaF)

[110. McRae, D. J., Lynham, T. J. & Frech, R. J. Understory prescribed burning in red pine and white pine. *THE FORESTRY CHRONICLE* **70**, 7 (1994).](https://www.zotero.org/google-docs/?Tq4xaF)

[111. Stocks, B. J. *et al.* Large forest fires in Canada, 1959–1997. *Journal of Geophysical Research: Atmospheres* **107**, FFR 5-1-FFR 5-12 (2002).](https://www.zotero.org/google-docs/?Tq4xaF)

[112. Erni, S., Arseneault, D., Parisien, M.-A. & Bégin, Y. Spatial and temporal dimensions of fire activity in the fire-prone eastern Canadian taiga. *Global Change Biology* **23**, 1152–1166 (2017).](https://www.zotero.org/google-docs/?Tq4xaF)

[113. Rowe, J. S. & Scotter, G. W. Fire in the boreal forest. *Quaternary Research* **3**, 444–464 (1973).](https://www.zotero.org/google-docs/?Tq4xaF)

[114. Christianson, A. C. *et al.* Centering Indigenous Voices: The Role of Fire in the Boreal Forest of North America. *Curr Forestry Rep* **8**, 257–276 (2022).](https://www.zotero.org/google-docs/?Tq4xaF)

[115. Lewis, H. T. *A Time for Burning*. (Boreal Institute for Northern Studies, University of Alberta, Edmonton, Alta., Canada, 1982).](https://www.zotero.org/google-docs/?Tq4xaF)

[116. Brown, J. K. & Smith, J. K. *Wildland Fire in Ecosystems: Effects of Fire on Flora*. RMRS-GTR-42-V2 https://www.fs.usda.gov/treesearch/pubs/4554 (2000) doi:10.2737/RMRS-GTR-42-V2.](https://www.zotero.org/google-docs/?Tq4xaF)

[117. Anderson, H. G. & Bailey, A. W. Effects of annual burning on grassland in the aspen parkland of east-central Alberta. *Can. J. Bot.* **58**, 985–996 (1980).](https://www.zotero.org/google-docs/?Tq4xaF)

[118. Nelson, J. G. & England, R. E. Some Comments on the Causes and Effects of Fire in the Northern Grasslands Area of Canada and the Nearby United States, Ca. 1750–1900. *The Canadian Geographer / Le Géographe canadien* **15**, 295–306 (1971).](https://www.zotero.org/google-docs/?Tq4xaF)

[119. Higgins, K. F. *Interpretation and Compendium of Historical Fire Accounts in the Northern Great Plains*. (U.S. Department of the Interior, Fish and Wildlife Service, 1986).](https://www.zotero.org/google-docs/?Tq4xaF)

[120. Driver, E. A. Fire on Grasslands – Friend or Foe? *Blue Jay* **45**, (1987).](https://www.zotero.org/google-docs/?Tq4xaF)

[121. Roy-Denis, C. Fire for Well-Being: Use of Prescribed Burning in the Northern Alberta Boreal Forest. *Earth Common Journal* **5**, 40–50 (2015).](https://www.zotero.org/google-docs/?Tq4xaF)

[122. Canadian Prairies Prescribed Fire Exchange. *Canadian Prairies Prescribed Fire Exchange* https://www.grasslandfire.ca.](https://www.zotero.org/google-docs/?Tq4xaF)

[123. Van Wagner, C. E. Fire and red pine. (1970).](https://www.zotero.org/google-docs/?Tq4xaF)

[124. Flannigan, M. Fire Regime and the Abundance of Red Pine. *International Journal of Wildland Fire* **3**, 241–247 (1993).](https://www.zotero.org/google-docs/?Tq4xaF)

[125. Hoffman, K. M. *et al.* The right to burn: barriers and opportunities for Indigenous-led fire stewardship in Canada. *FACETS* **7**, 464–481 (2022).](https://www.zotero.org/google-docs/?Tq4xaF)

[126. Dickson-Hoyle, S. & John, C. *Elephant Hill: Secwépemc Leadership and Lessons Learned from the Collective Story of Wildfire Recovery*. https://www.srssociety.com/docs/elephant_hill_-_secw%C3%A9pemc_leadership_and_lessons_learned.pdf (2021).](https://www.zotero.org/google-docs/?Tq4xaF)

[127. Coughlan, M. R. *Fire Use, Landscape Transition, and the Socioecological Strategies of Households in the French Western Pyrenees*. https://esploro.libs.uga.edu/esploro/outputs/9949334467902959 (2013).](https://www.zotero.org/google-docs/?Tq4xaF)

[128. Faerber, J. Prescribed Range Burning in the Pyrenees : From a Traditional Practice to a Modern Management Tool. in vol. 38 12–22 (International Forest Fire News, 2011).](https://www.zotero.org/google-docs/?Tq4xaF)

[129. Fernandes, P. *et al.* Prescribed burning in the European Mediterranean Basin. in 230–248 (2022).](https://www.zotero.org/google-docs/?Tq4xaF)

[130. *Best Practices of Fire Use: Prescribed Burning and Suppression Fire Programmes in Selected Case-Study Regions in Europe*. (European Forest Institute, Joensuu, 2010).](https://www.zotero.org/google-docs/?Tq4xaF)

[131. Goldammer, J. G. & Page, H. Fire History of Central Europe: Implications for Prescribed Burning in Landscape Management and Nature Conservation. (2000).](https://www.zotero.org/google-docs/?Tq4xaF)

[132. Valkó, O. & Deák, B. Increasing the potential of prescribed burning for the biodiversity conservation of European grasslands. *Current Opinion in Environmental Science & Health* **22**, 100268 (2021).](https://www.zotero.org/google-docs/?Tq4xaF)

[133. GFMC Team. Application of Prescribed Burning in Pine Stands in Germany to Reduce Wildfire Hazard. *International Forest Fire News* (2009).](https://www.zotero.org/google-docs/?Tq4xaF)

[134. Niemeyer, T., Niemeyer, M., Mohamed, A., Fottner, S. & Härdtle, W. Impact of prescribed burning on the nutrient balance of heathlands with particular reference to nitrogen and phosphorus. *Applied Vegetation Science* **8**, 183–192 (2005).](https://www.zotero.org/google-docs/?Tq4xaF)

[135. Hochkirch, A. & Adorf, F. Effects of prescribed burning and wildfires on Orthoptera in Central European peat bogs. *Environmental Conservation* **34**, 225–235 (2007).](https://www.zotero.org/google-docs/?Tq4xaF)

[136. Federal Environment Ministry. *Federal Nature Conservation Act*. (2009).](https://www.zotero.org/google-docs/?Tq4xaF)

[137. Agnoletti, M., Piras, F., Venturi, M. & Santoro, A. Cultural values and forest dynamics: The Italian forests in the last 150 years. *Forest Ecology and Management* **503**, 119655 (2022).](https://www.zotero.org/google-docs/?Tq4xaF)

[138. Ascoli, D. & Bovio, G. Prescribed burning in Italy: issues, advances and challenges. *iForest - Biogeosciences and Forestry* **6**, 79 (2013).](https://www.zotero.org/google-docs/?Tq4xaF)

[139. Ascoli, D., Moris, J. V., Marchetti, M. & Sallustio, L. Land use change towards forests and wooded land correlates with large and frequent wildfires in Italy. (2021) doi:10.12899/asr-2264.](https://www.zotero.org/google-docs/?Tq4xaF)

[140. Kirschner, J. A. *et al.* Governance drivers hinder and support a paradigm shift in wildfire risk management in Italy. *Reg Environ Change* **24**, 13 (2024).](https://www.zotero.org/google-docs/?Tq4xaF)

[141. Ascoli, D. *et al.* Prescribed burning and browsing to control tree encroachment in southern European heathlands. *Forest Ecology and Management* **289**, 69–77 (2013).](https://www.zotero.org/google-docs/?Tq4xaF)

[142. Borghesio, L. Effects of fire on the vegetation of a lowland heathland in North-western Italy. in *Herbaceous Plant Ecology: Recent Advances in Plant Ecology* (ed. Van der Valk, A. G.) 359–367 (Springer Netherlands, Dordrecht, 2009). doi:10.1007/978-90-481-2798-6_30.](https://www.zotero.org/google-docs/?Tq4xaF)

[143. Nota, G. *et al.* Sheep Grazing and Wildfire: Disturbance Effects on Dry Grassland Vegetation in the Western Italian Alps. *Agronomy* **11**, 6 (2021).](https://www.zotero.org/google-docs/?Tq4xaF)

[144. Nakagoshi, N. Forest fire and management in pine forest ecosystem in Japan. *Hikobia* **13**, 301–311 (2001).](https://www.zotero.org/google-docs/?Tq4xaF)

[145. Paletto, A., SERENO, C. & Furuido, H. Historical evolution of forest management in Europe and in Japan. *Bulletin of the Tokyo University Forests* **119**, (2008).](https://www.zotero.org/google-docs/?Tq4xaF)

[146. Masato Y., Dan I. & Yoshinobu H. Floristic characteristics of a floodplain tall-grass vegetation managed by winter burning in central Japan. *Vegetation Science* **30**, 1–15 (2013).](https://www.zotero.org/google-docs/?Tq4xaF)

[147. Cross Currents. Wakakusayama Mountain Burning (Nara City, Nara). http://www.crosscurrents.hawaii.edu/content.aspx?lang=eng&site=japan&theme=cal&subtheme=FESTIVAL&unit=JCAL035 (2003).](https://www.zotero.org/google-docs/?Tq4xaF)

[148. Japan National Tourism Organization. Akiyoshidai Plateau | Travel Japan - Japan National Tourism Organization (Official Site). *Travel Japan* https://www.japan.travel/en/spot/1979/ (2024).](https://www.zotero.org/google-docs/?Tq4xaF)

[149. japan-guide. Sengokuhara Pampas Grass Field. https://www.japan-guide.com/e/e5218.html (2024).](https://www.zotero.org/google-docs/?Tq4xaF)

[150. Sanbesan Kouiki Tourism Shinko Kyougikai. Nishinohara Field Burning | Mt. Sanbe Area Tourist Information - A Sacred Mountain in the Daisen-Oki National Park. https://www.sanbesan.jp/en/touristspot/4668/ (2024).](https://www.zotero.org/google-docs/?Tq4xaF)

[151. Nagata, Y. K. & Ushimaru, A. Traditional burning and mowing practices support high grassland plant diversity by providing intermediate levels of vegetation height and soil pH. *Applied Vegetation Science* **19**, 567–577 (2016).](https://www.zotero.org/google-docs/?Tq4xaF)

[152. Jun, K. & Mihoko, K. Analysis of effects of burning in grasslands with quantifying succession stages by life-history traits in Kirigamine, central Japan. *Journal of Ecology and Environment* **36**, 101–112 (2013).](https://www.zotero.org/google-docs/?Tq4xaF)

[153. Kamada, M. & Nakagoshi, N. Influence of cultural factors on landscapes of mountainous farm villages in western Japan. *Landscape and Urban Planning* **37**, 85–90 (1997).](https://www.zotero.org/google-docs/?Tq4xaF)

[154. Koyama, A., Koyanagi, T. F., Akasaka, M., Takada, M. & Okabe, K. Combined burning and mowing for restoration of abandoned semi-natural grasslands. *Applied Vegetation Science* **20**, 40–49 (2017).](https://www.zotero.org/google-docs/?Tq4xaF)

[155. Rodríguez-Trejo, D. A. Fire Regimes, Fire Ecology, and Fire Management in Mexico. *ambi* **37**, 548–556 (2008).](https://www.zotero.org/google-docs/?Tq4xaF)

[156. Dalle, S. P., Pulido, M. T. & de Blois, S. Balancing shifting cultivation and forest conservation: lessons from a ‘sustainable landscape’ in southeastern Mexico. *Ecological Applications* **21**, 1557–1572 (2011).](https://www.zotero.org/google-docs/?Tq4xaF)

[157. Fulé, P. Z., Ramos-Gómez, M., Cortés-Montaño, C. & Miller, A. M. Fire regime in a Mexican forest under indigenous resource management. *Ecological Applications* **21**, 764–775 (2011).](https://www.zotero.org/google-docs/?Tq4xaF)

[158. Miller, A. M. & Chambers, C. L. BIRDS OF HARVESTED AND UNHARVESTED PINE-OAK FORESTS, CHIHUAHUA, MEXICO. *swna* **52**, 271–283 (2007).](https://www.zotero.org/google-docs/?Tq4xaF)

[159. Nigh, R. & Diemont, S. A. The Maya milpa: fire and the legacy of living soil. *Frontiers in Ecology and the Environment* **11**, e45–e54 (2013).](https://www.zotero.org/google-docs/?Tq4xaF)

[160. Rodríguez-Trejo, D. A., Martínez-Hernández, P. A., Ortiz-Contla, H., Chavarría-Sánchez, M. R. & Hernández-Santiago, F. The Present Status of Fire Ecology, Traditional Use of Fire, and Fire Management in Mexico and Central America. *fire ecol* **7**, 40–56 (2011).](https://www.zotero.org/google-docs/?Tq4xaF)

[161. Schumacher, M., Durán-Díaz, P., Kurjenoja, A. K., Gutiérrez-Juárez, E. & González-Rivas, D. A. Evolution and Collapse of Ejidos in Mexico—To What Extent Is Communal Land Used for Urban Development? *Land* **8**, 146 (2019).](https://www.zotero.org/google-docs/?Tq4xaF)

[162. Guevara-Hernández, F. *et al.* Traditional fire use, governance and social dynamics in a Biosphere Reserve of Chiapas, Mexico. *La Pensée* **75**, (2013).](https://www.zotero.org/google-docs/?Tq4xaF)

[163. Sheridan, RachelA. S., Fulé, P., Lee, M. & Nielsen, E. Identifying Social-ecological Linkages to Develop a Community Fire Plan in Mexico. *Conservat Soc* **13**, 395 (2015).](https://www.zotero.org/google-docs/?Tq4xaF)

[164. Monzón-Alvarado, C. M. & Keys, E. Synergistic vulnerabilities: climate variability and fire management policy increase farming challenges in southeastern Mexico. *Reg Environ Change* **17**, 489–500 (2017).](https://www.zotero.org/google-docs/?Tq4xaF)

[165. Mathews, A. S. Power/Knowledge, Power/Ignorance: Forest Fires and the State in Mexico. *Hum Ecol* **33**, 795–820 (2005).](https://www.zotero.org/google-docs/?Tq4xaF)

[166. Drury, S. A. & Veblen, T. T. Spatial and temporal variability in fire occurrence within the Las Bayas Forestry Reserve, Durango, Mexico. *Plant Ecol* **197**, 299–316 (2008).](https://www.zotero.org/google-docs/?Tq4xaF)

[167. Lawrence, T. J., Stedman, R. C., Morreale, S. J. & Taylor, S. R. Rethinking Landscape Conservation: Linking Globalized Agriculture to Changes to Indigenous Community-Managed Landscapes. *Tropical Conservation Science* **12**, 1940082919889503 (2019).](https://www.zotero.org/google-docs/?Tq4xaF)

[168. Pérez-Salicrup, D. R. *et al.* Institutional coordination of prescribed and controlled burns in Mexico. *Revista mexicana de ciencias forestales* **9**, 252–270 (2018).](https://www.zotero.org/google-docs/?Tq4xaF)

[169. Naveh, Z. The evolutionary significance of fire in the mediterranean region. *Plant Ecol* **29**, 199–208 (1975).](https://www.zotero.org/google-docs/?Tq4xaF)

[170. Pausas, J. G. & Vallejo, V. R. The role of fire in European Mediterranean ecosystems. in *Remote Sensing of Large Wildfires* (ed. Chuvieco, E.) 3–16 (Springer Berlin Heidelberg, Berlin, Heidelberg, 1999). doi:10.1007/978-3-642-60164-4_2.](https://www.zotero.org/google-docs/?Tq4xaF)

[171. Nunes, L. J. R., Meireles, C. I. R., Pinto Gomes, C. J. & Almeida Ribeiro, N. M. C. Historical Development of the Portuguese Forest: The Introduction of Invasive Species. *Forests* **10**, 974 (2019).](https://www.zotero.org/google-docs/?Tq4xaF)

[172. Reboredo, F. & Pais, J. Evolution of forest cover in Portugal: A review of the 12th–20th centuries. *Journal of Forestry Research* **25**, 249–256 (2014).](https://www.zotero.org/google-docs/?Tq4xaF)

[173. Fernandes, P. & Botelho, H. Analysis of the prescribed burning practice in the pine forest of northwestern Portugal. *Journal of Environmental Management* **70**, 15–26 (2004).](https://www.zotero.org/google-docs/?Tq4xaF)

[174. Garcia-Gonzalo, J. *et al.* Modelling wildfire risk in pure and mixed forest stands in Portugal. *Allgemeine Forst und Jagdzeitung* **183**, 238–248 (2012).](https://www.zotero.org/google-docs/?Tq4xaF)

[175. Alegria, C., Roque, N., Albuquerque, T., Fernandez, P. & Ribeiro, M. M. Modelling Maritime Pine (Pinus pinaster Aiton) Spatial Distribution and Productivity in Portugal: Tools for Forest Management. *Forests* **12**, 368 (2021).](https://www.zotero.org/google-docs/?Tq4xaF)

[176. Fernandes, P. M. & Botelho, H. S. A review of prescribed burning effectiveness in fire hazard reduction. *Int. J. Wildland Fire* **12**, 117–128 (2003).](https://www.zotero.org/google-docs/?Tq4xaF)

[177. Pyne, S. Wild Hearth: A Prolegomenon to the Cultural Fire History of Northern Eurasia. in *Fire in Ecosystems of Boreal Eurasia* (eds. Goldammer, J. G. & Furyaev, V.) (Springer Science & Business Media, 2013).](https://www.zotero.org/google-docs/?Tq4xaF)

[178. Vinokurova, L., Solovyeva, V. & Filippova, V. When Ice Turns to Water: Forest Fires and Indigenous Settlements in the Republic of Sakha (Yakutia). *Sustainability* **14**, 4759 (2022).](https://www.zotero.org/google-docs/?Tq4xaF)

[179. *Prescribed Burning in Russia and Neighbouring Temperate-Boreal Eurasia: A Publication of the Global Fire Monitoring Center (GFMC)*. (Kessel, Remagen-Oberwinter, 2013).](https://www.zotero.org/google-docs/?Tq4xaF)

[180. Shorohova, E., Sinkevich, S., Kryshen, A. & Vanha-Majamaa, I. Variable retention forestry in European boreal forests in Russia. *Ecol Process* **8**, 34 (2019).](https://www.zotero.org/google-docs/?Tq4xaF)

[181. Hall, J. V., Loboda, T. V., Giglio, L. & McCarty, G. W. A MODIS-based burned area assessment for Russian croplands: Mapping requirements and challenges. *Remote Sensing of Environment* **184**, 506–521 (2016).](https://www.zotero.org/google-docs/?Tq4xaF)

[182. *Forest Code of the Russian Federation*. *580* (2019).](https://www.zotero.org/google-docs/?Tq4xaF)

[183. Baikal Basin Information Center. 053. Timber stock of the main groups of forest forming tree species map — English. http://bic.iwlearn.org/en/atlas/atlas/53-timber-stock-of-the-main-groups-of-forest-forming-tree-species-map.](https://www.zotero.org/google-docs/?Tq4xaF)

[184. Kukavskaya, E. A., Buryak, L. V., Shvetsov, E. G., Conard, S. G. & Kalenskaya, O. P. The impact of increasing fire frequency on forest transformations in southern Siberia. *Forest Ecology and Management* **382**, 225–235 (2016).](https://www.zotero.org/google-docs/?Tq4xaF)

[185. Vaganov, E. A., Buzykin, A. I. & Evdokimenko, M. D. Forest Resources of the Lake Baikal Region and Perspectives of their Industrial Exploitation. in *Sustainable Development of the Lake Baikal Region* (eds. Koptyug, V. A. & Uppenbrink, M.) 211–216 (Springer, Berlin, Heidelberg, 1996). doi:10.1007/978-3-642-61429-3_24.](https://www.zotero.org/google-docs/?Tq4xaF)

[186. Global Forest Watch. Irkutsk, Russia Interactive Forest Map. https://www.globalforestwatch.org/map/country/RUS/18/?category=forest-change&dashboardPrompts=eyJzaG93UHJvbXB0cyI6dHJ1ZSwicHJvbXB0c1ZpZXdlZCI6WyJzdWJzY3JpYmVUb0FyZWEiXSwic2V0dGluZ3MiOnsib3BlbiI6ZmFsc2UsInN0ZXBJbmRleCI6MCwic3RlcHNLZXkiOiIifSwib3BlbiI6dHJ1ZSwic3RlcHNLZXkiOiJkb3dubG9hZERhc2hib2FyZFN0YXRzIn0%3D&map=eyJjZW50ZXIiOnsibGF0Ijo2MC4xMDg2NzA0NjMwNDkzNiwibG5nIjo4MS4xOTk0NTc1MzMzMTU0MX0sImJhc2VtYXAiOnsidmFsdWUiOiJwbGFuZXQiLCJjb2xvciI6IiIsIm5hbWUiOiJwbGFuZXRfbWVkcmVzX3Zpc3VhbF8yMDIyLTExX21vc2FpYyIsImltYWdlVHlwZSI6InZpc3VhbCJ9LCJjYW5Cb3VuZCI6ZmFsc2UsImRhdGFzZXRzIjpbeyJkYXRhc2V0IjoidHJlZS1jb3Zlci1sb3NzLWJ5LWRvbWluYW50LWRyaXZlciIsIm9wYWNpdHkiOjEsInZpc2liaWxpdHkiOnRydWUsImxheWVycyI6WyJ0cmVlLWNvdmVyLWxvc3MtYnktZG9taW5hbnQtZHJpdmVyIl19LHsiZGF0YXNldCI6InBvbGl0aWNhbC1ib3VuZGFyaWVzIiwibGF5ZXJzIjpbImRpc3B1dGVkLXBvbGl0aWNhbC1ib3VuZGFyaWVzIiwicG9saXRpY2FsLWJvdW5kYXJpZXMiXSwiYm91bmRhcnkiOnRydWUsIm9wYWNpdHkiOjEsInZpc2liaWxpdHkiOnRydWV9XX0%3D&mapMenu=eyJkYXRhc2V0Q2F0ZWdvcnkiOiJmb3Jlc3RDaGFuZ2UifQ%3D%3D&mapPrompts=eyJvcGVuIjp0cnVlLCJzdGVwc0tleSI6InJlY2VudEltYWdlcnkiLCJzdGVwc0luZGV4IjowfQ%3D%3D&showMap=true.](https://www.zotero.org/google-docs/?Tq4xaF)

[187. Swetnam, T. W. Fire and Climate History in the Central Yenisey Region, Siberia. in *Fire in Ecosystems of Boreal Eurasia* (eds. Goldammer, J. G. & Furyaev, V. V.) 90–104 (Springer Netherlands, Dordrecht, 1996). doi:10.1007/978-94-015-8737-2_6.](https://www.zotero.org/google-docs/?Tq4xaF)

[188. Narita, D., Gavrilyeva, T. & Isaev, A. Impacts and management of forest fires in the Republic of Sakha, Russia: A local perspective for a global problem. *Polar Science* **27**, 100573 (2021).](https://www.zotero.org/google-docs/?Tq4xaF)

[189. Kirillina, K., Shvetsov, E. G., Protopopova, V. V., Thiesmeyer, L. & Yan, W. Consideration of anthropogenic factors in boreal forest fire regime changes during rapid socio-economic development: case study of forestry districts with increasing burnt area in the Sakha Republic, Russia. *Environ. Res. Lett.* **15**, 035009 (2020).](https://www.zotero.org/google-docs/?Tq4xaF)

[190. Pettus, A. *Agricultural Fires and Arctic Climate Change*. https://www.catf.us/resource/agricultural-fires-and-arctic-climate-change/ (2009).](https://www.zotero.org/google-docs/?Tq4xaF)

[191. Korontzi, S., McCarty, J., Loboda, T., Kumar, S. & Justice, C. Global distribution of agricultural fires in croplands from 3 years of Moderate Resolution Imaging Spectroradiometer (MODIS) data. *Global Biogeochemical Cycles* **20**, (2006).](https://www.zotero.org/google-docs/?Tq4xaF)

[192. Bands, D. P. Prescribed Burning in Cape Fynbos Catchments. in *Proceedings of the Symposium on the Environmental Consequences of Fire and Fuel Management in Mediterranean Ecosystems, August 1-5, 1977, Palo Alto, California* (Department of Agriculture, Forest Service, 1977).](https://www.zotero.org/google-docs/?Tq4xaF)

[193. Kraaij, T. & Van Wilgen, B. W. Drivers, ecology, and management of fire in fynbos. in *Fynbos: Ecology, Evolution, and Conservation of a Megadiverse Region* (OUP Oxford, 2014).](https://www.zotero.org/google-docs/?Tq4xaF)

[194. van Wilgen, B. W. Fire management in species-rich Cape fynbos shrublands. *Frontiers in Ecology and the Environment* **11**, e35–e44 (2013).](https://www.zotero.org/google-docs/?Tq4xaF)

[195. Van Wilgen, B. W. The evolution of fire management practices in savanna protected areas in South Africa. *S Afr J Sci* **105**, 343 (2010).](https://www.zotero.org/google-docs/?Tq4xaF)

[196. Van Wilgen, B. W., Govender, N. & Biggs, H. C. The contribution of fire research to fire management: a critical review of a long-term experiment in the Kruger National Park, South Africa. *Int. J. Wildland Fire* **16**, 519–530 (2007).](https://www.zotero.org/google-docs/?Tq4xaF)

[197. Lohmann, D., Tietjen, B., Blaum, N., Joubert, D. F. & Jeltsch, F. Prescribed fire as a tool for managing shrub encroachment in semi-arid savanna rangelands. *Journal of Arid Environments* **107**, 49–56 (2014).](https://www.zotero.org/google-docs/?Tq4xaF)

[198. Turpie, J. *et al. TOWARDS A POLICY ON INDIGENOUS BUSH ENCROACHMENT*. https://www.dffe.gov.za/sites/default/files/reports/indigenousbushencroachment.pdf (2019).](https://www.zotero.org/google-docs/?Tq4xaF)

[199. Dye, P. & Versfeld, D. Managing the hydrological impacts of South African plantation forests: An overview. *Forest Ecology and Management* **251**, 121–128 (2007).](https://www.zotero.org/google-docs/?Tq4xaF)

[200. Morris, A. R. Changing use of species and hybrids in South African forest plantations. *Southern Forests: a Journal of Forest Science* **84**, 193–205 (2022).](https://www.zotero.org/google-docs/?Tq4xaF)

[201. South African National Biodiversity Institute. Acacia stricta. https://www.sanbi.org/resources/infobases/invasive-alien-plant-alert/acacia-stricta/, https://www.sanbi.org/resources/infobases/invasive-alien-plant-alert/acacia-stricta/ (2018).](https://www.zotero.org/google-docs/?Tq4xaF)

[202. Vadell, E., de-Miguel, S. & Pemán, J. Large-scale reforestation and afforestation policy in Spain: A historical review of its underlying ecological, socioeconomic and political dynamics. *Land Use Policy* **55**, 37–48 (2016).](https://www.zotero.org/google-docs/?Tq4xaF)

[203. Pausas, J. G. & Fernández-Muñoz, S. Fire regime changes in the Western Mediterranean Basin: from fuel-limited to drought-driven fire regime. *Climatic Change* **110**, 215–226 (2012).](https://www.zotero.org/google-docs/?Tq4xaF)

[204. Moreno, M. V., Conedera, M., Chuvieco, E. & Pezzatti, G. B. Fire regime changes and major driving forces in Spain from 1968 to 2010. *Environmental Science & Policy* **37**, 11–22 (2014).](https://www.zotero.org/google-docs/?Tq4xaF)

[205. Corbelle-Rico, E., Crecente-Maseda, R. & Santé-Riveira, I. Multi-scale assessment and spatial modelling of agricultural land abandonment in a European peripheral region: Galicia (Spain), 1956–2004. *Land Use Policy* **29**, 493–501 (2012).](https://www.zotero.org/google-docs/?Tq4xaF)

[206. Gómez-García, E. Estimating the changes in tree carbon stocks in Galician forests (NW Spain) between 1972 and 2009. *Forest Ecology and Management* **467**, 118157 (2020).](https://www.zotero.org/google-docs/?Tq4xaF)

[207. Cogos, S., Roturier, S. & Östlund, L. The origins of prescribed burning in Scandinavian forestry: the seminal role of Joel Wretlind in the management of fire-dependent forests. *Eur J Forest Res* **139**, 393–406 (2020).](https://www.zotero.org/google-docs/?Tq4xaF)

[208. Hermanson, V. C. Prescribed Burning in Sweden. (2020).](https://www.zotero.org/google-docs/?Tq4xaF)

[209. Natur, E. *The Contribution of FSC Certification to Biodiversity in Swedish Forests*. https://www.se.fsc.org/sites/default/files/2022-02/The%20contribution%20of%20FSC%20certification%20to%20biodiversity%20in%20Swedish%20forests_0.pdf (2013).](https://www.zotero.org/google-docs/?Tq4xaF)

[210. Life Taiga. Fire brings new life to woods. (2021).](https://www.zotero.org/google-docs/?Tq4xaF)

[211. Ramberg, E., Strengbom, J. & Granath, G. Coordination through databases can improve prescribed burning as a conservation tool to promote forest biodiversity. *Ambio* **47**, 298–306 (2018).](https://www.zotero.org/google-docs/?Tq4xaF)

[212. Schmidt-Vogt, D. Secondary Forests in Swidden Agriculture in the Highlands of Thailand. *Journal of Tropical Forest Science* (2001).](https://www.zotero.org/google-docs/?Tq4xaF)

[213. Makarabhirom, P., Ganz, D. & Onprom, S. Community involvement in fire management: cases and recommendations for community-based fire management in Thailand. in *Communities in flames: proceedings of an international conference on community involvement in fire management* (2002).](https://www.zotero.org/google-docs/?Tq4xaF)

[214. Schmidt-Vogt, D. *et al.* An Assessment of Trends in the Extent of Swidden in Southeast Asia. *Hum Ecol* **37**, 269–280 (2009).](https://www.zotero.org/google-docs/?Tq4xaF)

[215. Lakanavichian, S. Trends in forest ownership, forest resources tenure and institutional arrangements: are they contributing to better forest management and poverty reduction? Case study from Thailand. in *Forestry Policy and Institutions Working Paper 14* (Rome, 2006).](https://www.zotero.org/google-docs/?Tq4xaF)

[216. Junpen, A., Garivait, S. & Bonnet, S. Estimating emissions from forest fires in Thailand using MODIS active fire product and country specific data. *Asia-Pacific J Atmos Sci* **49**, 389–400 (2013).](https://www.zotero.org/google-docs/?Tq4xaF)

[217. RECOFTC. *Thailand’s Community Forest Act: Analysis of the Legal Framework and Recommendations*. (2021).](https://www.zotero.org/google-docs/?Tq4xaF)

[218. Thammanu, S., Han, H., Ekanayake, E. M. B. P., Jung, Y. & Chung, J. The Impact on Ecosystem Services and the Satisfaction Therewith of Community Forest Management in Northern Thailand. *Sustainability* **13**, 13474 (2021).](https://www.zotero.org/google-docs/?Tq4xaF)

[219. Goldammer, J. G. & Peñafiel, S. R. Fire in the Pine-Grassland Biomes of Tropical and Subtropical Asia. in *Fire in the Tropical Biota: Ecosystem Processes and Global Challenges* (ed. Goldammer, J. G.) 45–62 (Springer, Berlin, Heidelberg, 1990). doi:10.1007/978-3-642-75395-4_4.](https://www.zotero.org/google-docs/?Tq4xaF)

[220. Goldammer, J. G. & Wanthongchai, K. Fire Management in South Asia’s Dry Forests: Colonial Approaches, Current Problems and Perspectives. in *Tropical Forestry Change in a Changing World* (Kasetsart University Faculty of Forestry, Kasetsart University, Bangkok, Thailand, 2008).](https://www.zotero.org/google-docs/?Tq4xaF)

[221. Wanthongchai, K., Bauhus, J. & Goldammer, J. G. Nutrient losses through prescribed burning of aboveground litter and understorey in dry dipterocarp forests of different fire history. *CATENA* **74**, 321–332 (2008).](https://www.zotero.org/google-docs/?Tq4xaF)

[222. Kutintara, U. STRUCTURE OF THE DRY DIPTEROCARP FOREST. (Colorado State University, 1975).](https://www.zotero.org/google-docs/?Tq4xaF)

[223. Arunrat, N., Pumijumnong, N. & Sereenonchai, S. Air-Pollutant Emissions from Agricultural Burning in Mae Chaem Basin, Chiang Mai Province, Thailand. *Atmosphere* **9**, 145 (2018).](https://www.zotero.org/google-docs/?Tq4xaF)

[224. Pasukphun, N. ENVIRONMENTAL HEALTH BURDEN OF OPEN BURNING IN NORTHERN THAILAND: A REVIEW. *PSRU Journal of Science and Technology* **3**, 11–28 (2018).](https://www.zotero.org/google-docs/?Tq4xaF)

[225. Davies, M. G., Gray, A., Hamilton, A. & Legg, C. J. The future of fire management in the British uplands. *International Journal of Biodiversity Science & Management* **4**, 127–147 (2008).](https://www.zotero.org/google-docs/?Tq4xaF)

[226. Yallop, A. R. *et al.* The extent and intensity of management burning in the English uplands. *Journal of Applied Ecology* **43**, 1138–1148 (2006).](https://www.zotero.org/google-docs/?Tq4xaF)

[227. Ground nesting birds in the New Forest. (2024).](https://www.zotero.org/google-docs/?Tq4xaF)

[228. Smith, B. M. *et al.* Resolving a heated debate: The utility of prescribed burning as a management tool for biodiversity on lowland heath. *Journal of Applied Ecology* **60**, 2040–2051 (2023).](https://www.zotero.org/google-docs/?Tq4xaF)

[229. Worrall, F., Clay, G. D., Marrs, R. & Reed, M. S. *Impacts of Burning Management on Peatlands*. https://www.iucn-uk-peatlandprogramme.org/sites/www.iucn-uk-peatlandprogramme.org/files/images/Review%20Impacts%20of%20Burning%20on%20Peatlands,%20June%202011%20Final.pdf (2010).](https://www.zotero.org/google-docs/?Tq4xaF)

[230. Douglas, D. J. T. *et al.* Vegetation burning for game management in the UK uplands is increasing and overlaps spatially with soil carbon and protected areas. *Biological Conservation* **191**, 243–250 (2015).](https://www.zotero.org/google-docs/?Tq4xaF)

[231. Harper, A. R., Doerr, S. H., Santin, C., Froyd, C. A. & Sinnadurai, P. Prescribed fire and its impacts on ecosystem services in the UK. *Science of The Total Environment* **624**, 691–703 (2018).](https://www.zotero.org/google-docs/?Tq4xaF)

[232. *The Heather and Grass Burning (England) Regulations 2007*. vol. 2003 (King’s Printer of Acts of Parliament, 2007).](https://www.zotero.org/google-docs/?Tq4xaF)

[233. Farage, P., Ball, A., McGenity, T. J., Whitby, C. & Pretty, J. Burning management and carbon sequestration of upland heather moorland in the UK. *Soil Res.* **47**, 351–361 (2009).](https://www.zotero.org/google-docs/?Tq4xaF)

[234. Diaz, J., Fawcett, J. E. & Weir, J. R. The Value of Forming a Prescribed Burn Association (PBA). (2021).](https://www.zotero.org/google-docs/?Tq4xaF)

[235. Brenner, J. & Wade, D. Florida’s Revised Prescribed Fire Law: Protection For Responsible Burners. *Pages 132-136 in K.E.M. Galley, R.C. Klinger, and N.G. Sugihara (eds.). Proceedings of Fire Conference 2000: The First National Congress on Fire Ecology, Prevention, and Management. Miscellaneous Publication No. 13, Tall Timbers Research Station, Tallahassee, FL.* (2022).](https://www.zotero.org/google-docs/?Tq4xaF)

[236. Johnson, A. S. & Hale, P. E. The historical foundations of prescribed burning for wildlife: a southeastern perspective. *In: Ford, W. Mark; Russell, Kevin R.; Moorman, Christopher E., eds. Proceedings: the role of fire for nongame wildlife management and community restoration: traditional uses and new directions. Gen. Tech. Rep. NE-288. Newtown Square, PA: U.S. Dept. of Agriculture, Forest Service, Northeastern Research Station. 11-23.* (2002).](https://www.zotero.org/google-docs/?Tq4xaF)

[237. Stambaugh, M. C., Guyette, R. P. & Marschall, J. M. Longleaf pine (Pinus palustris Mill.) fire scars reveal new details of a frequent fire regime. *Journal of Vegetation Science* **22**, 1094–1104 (2011).](https://www.zotero.org/google-docs/?Tq4xaF)

[238. White, C. R. & Harley, G. L. Historical fire in longleaf pine (inus palustris) forests of south Mississippi and its relation to land use and climate. *Ecosphere* **7**, e01458 (2016).](https://www.zotero.org/google-docs/?Tq4xaF)

[239. Frost, C. C. PRESETTLEMENT FIRE FREQUENCY REGIMES OF THE UNITED STATES: A FIRST APPROXIMATION. (1998).](https://www.zotero.org/google-docs/?Tq4xaF)

[240. Fowler, C. & Konopik, E. The History of Fire in the Southern United States. *Human Ecology Review* **14**, 165–176 (2007).](https://www.zotero.org/google-docs/?Tq4xaF)

[241. Stoddard, H. L. *The Bobwhite Quail: Its Habits, Preservation and Increase*. (Scribner, 1931).](https://www.zotero.org/google-docs/?Tq4xaF)

[242. Jose, S., Jokela, E. J. & Miller, D. L. The Longleaf Pine Ecosystem. in *The Longleaf Pine Ecosystem: Ecology, Silviculture, and Restoration* (eds. Jose, S., Jokela, E. J. & Miller, D. L.) 3–8 (Springer, New York, NY, 2006). doi:10.1007/978-0-387-30687-2_1.](https://www.zotero.org/google-docs/?Tq4xaF)

[243. Mushinsky, H., McCoy, E., Berish, J., Ashton, R. & Wilson, D. Gopherus polyphemus – Gopher Tortoise. *Chelonian Research Monographs* **3**, 350–375 (2006).](https://www.zotero.org/google-docs/?Tq4xaF)

[244. Ashton, K. G., Engelhardt, B. M. & Branciforte, B. S. Gopher Tortoise (Gopherus polyphemus) Abundance and Distribution after Prescribed Fire Reintroduction to Florida Scrub and Sandhill at Archbold Biological Station. *hpet* **42**, 523–529 (2008).](https://www.zotero.org/google-docs/?Tq4xaF)

[245. Bollinger, R. *et al. 2021 ALRI Accomplishment Report*. (2021).](https://www.zotero.org/google-docs/?Tq4xaF)

[246. *Ecological Restoration and Management of Longleaf Pine Forests*. (CRC Press, Boca Raton, 2017). doi:10.1201/9781315152141.](https://www.zotero.org/google-docs/?Tq4xaF)

[247. Roos, C. I., Zedeño, M. N., Hollenback, K. L. & Erlick, M. M. H. Indigenous impacts on North American Great Plains fire regimes of the past millennium. *Proceedings of the National Academy of Sciences* **115**, 8143–8148 (2018).](https://www.zotero.org/google-docs/?Tq4xaF)

[248. Engle, D. M., Coppedge, B. R. & Fuhlendorf, S. D. From the Dust Bowl to the Green Glacier: Human Activity and Environmental Change in Great Plains Grasslands. in *Western North American Juniperus Communities: A Dynamic Vegetation Type* (ed. Van Auken, O. W.) 253–271 (Springer, New York, NY, 2008). doi:10.1007/978-0-387-34003-6_14.](https://www.zotero.org/google-docs/?Tq4xaF)

[249. Twidwell, D. *et al.* The rising Great Plains fire campaign: citizens’ response to woody plant encroachment. *Frontiers in Ecology and the Environment* **11**, e64–e71 (2013).](https://www.zotero.org/google-docs/?Tq4xaF)

[250. Landscape Partnership. Prescribed Burn Associations. https://www.landscapepartnership.org/networks/working-lands-for-wildlife/wildland-fire/prescribed-burning/prescribed-burn-associations/prescribed-burn-associations-page (2024).](https://www.zotero.org/google-docs/?Tq4xaF)

[251. Great Plains Fire Science Exchange. Prescribed Burn Associations. https://kstate.maps.arcgis.com/apps/webappviewer/index.html?id=3eacaaf1a3514d3da2e5215b5dd55f9b (2024).](https://www.zotero.org/google-docs/?Tq4xaF)

[252. Weir, J., Twidwell, D. & Wonkka, C. *Prescribed Burn Association Activity, Needs, and Safety Record: A Survey of the Great Plains*. (2015).](https://www.zotero.org/google-docs/?Tq4xaF)

[253. Swetnam, T. & Baisan, C. Historical Fire Regime Patterns in the Southwestern United States Since AD 1700. *USDA Forest Service General Technical Report RMRS* **GTR 286**, 11–32 (1996).](https://www.zotero.org/google-docs/?Tq4xaF)

[254. McKelvey, K. S. *et al.* An Overview of Fire in the Sierra Nevada. *Sierra Nevada Ecosystem Project: Final report to Congress, vol. II: Assessments and scientific basis for management options* (1996).](https://www.zotero.org/google-docs/?Tq4xaF)

[255. Taylor, A. H. & Skinner, C. N. Spatial Patterns and Controls on Historical Fire Regimes and Forest Structure in the Klamath Mountains. *Ecological Applications* **13**, 704–719 (2003).](https://www.zotero.org/google-docs/?Tq4xaF)

[256. Leopold, A. S., Cain, S. A., Cottam, C. M., Gabrielson, I. N. & Kimball, T. L. *Wildlife Management in the National Parks*. (1963).](https://www.zotero.org/google-docs/?Tq4xaF)

[257. Kilgore, B. M. & Taylor, D. Fire History of a Sequoia-Mixed Conifer Forest. *Ecology* **60**, 129–142 (1979).](https://www.zotero.org/google-docs/?Tq4xaF)

[258. Schoennagel, T., Veblen, T. T. & Romme, W. H. The Interaction of Fire, Fuels, and Climate across Rocky Mountain Forests. *BioScience* **54**, 661–676 (2004).](https://www.zotero.org/google-docs/?Tq4xaF)

[259. Prichard, S. J., Peterson, D. L. & Jacobson, K. Fuel treatments reduce the severity of wildfire effects in dry mixed conifer forest, Washington, USA. *Can. J. For. Res.* **40**, 1615–1626 (2010).](https://www.zotero.org/google-docs/?Tq4xaF)

[260. Prichard, S. J. *et al.* Adapting western North American forests to climate change and wildfires: 10 common questions. *Ecological Applications* **31**, e02433 (2021).](https://www.zotero.org/google-docs/?Tq4xaF)

[261. Hunter, M. E. & Robles, M. D. Tamm review: The effects of prescribed fire on wildfire regimes and impacts: A framework for comparison. *Forest Ecology and Management* **475**, 118435 (2020).](https://www.zotero.org/google-docs/?Tq4xaF)

[262. Knapp, E. E., Estes, B. L. & Skinner, C. N. *Ecological Effects of Prescribed Fire Season: A Literature Review and Synthesis for Managers*. PSW-GTR-224 https://www.fs.usda.gov/treesearch/pubs/33628 (2009) doi:10.2737/PSW-GTR-224.](https://www.zotero.org/google-docs/?Tq4xaF)

[263. Romme, W. H. *et al.* Historical and Modern Disturbance Regimes, Stand Structures, and Landscape Dynamics in Piñon–Juniper Vegetation of the Western United States. *Rangeland Ecology & Management* **62**, 203–222 (2009).](https://www.zotero.org/google-docs/?Tq4xaF)

[264. Miller, R. & Tausch, R. THE ROLE OF FIRE IN JUNIPER AND PINYON WOODLANDS: A DESCRIPTIVE ANALYSIS. *Tall Timbers Research Station Miscellaneous Publication No. 11* **11**, (2001).](https://www.zotero.org/google-docs/?Tq4xaF)

[265. Muldavin, E. & Triepke, F. J. North American Pinyon–Juniper Woodlands: Ecological Composition, Dynamics, and Future Trends. in *Encyclopedia of the World’s Biomes* 516–531 (Elsevier, 2020). doi:10.1016/B978-0-12-409548-9.12113-X.](https://www.zotero.org/google-docs/?Tq4xaF)

[266. Everett, R. L. Plant Response to Fire in the Pinyon-Juniper Zone. in *Proceedings, Pinyon-Juniper Conference, Reno, NV, January 13-16, 1986* (U.S. Department of Agriculture, Forest Service, Intermountain Forest and Range Experiment Station, 1987).](https://www.zotero.org/google-docs/?Tq4xaF)

[267. Kobziar, L. N., Godwin, D., Taylor, L. & Watts, A. C. Perspectives on Trends, Effectiveness, and Impediments to Prescribed Burning in the Southern U.S. *Forests* **6**, 561–580 (2015).](https://www.zotero.org/google-docs/?Tq4xaF)

[268. Natcher, D. C. *et al.* Factors Contributing to the Cultural and Spatial Variability of Landscape Burning by Native Peoples of Interior Alaska. *Ecology and Society* **12**, (2007).](https://www.zotero.org/google-docs/?Tq4xaF)

[269. DeWilde, L. & Chapin, F. S. Human Impacts on the Fire Regime of Interior Alaska: Interactions among Fuels, Ignition Sources, and Fire Suppression. *Ecosystems* **9**, 1342–1353 (2006).](https://www.zotero.org/google-docs/?Tq4xaF)

[270. Fryer, J. L. Picea mariana. In: Fire Effects Information System. https://www.fs.usda.gov/database/feis/plants/tree/picmar/all.html (2014).](https://www.zotero.org/google-docs/?Tq4xaF)

[271. Duffy, P. A., Walsh, J. E., Graham, J. M., Mann, D. H. & Rupp, T. S. Impacts of Large-Scale Atmospheric–Ocean Variability on Alaskan Fire Season Severity. *Ecological Applications* **15**, 1317–1330 (2005).](https://www.zotero.org/google-docs/?Tq4xaF)

[272. Todd, S. K. & Jewkes, H. A. WILDLAND FIRE IN ALASKA: A history of organized fire suppression and management in the last frontier. *Agricultural and Forestry Experiment Station Bulletin* **114**, (2006).](https://www.zotero.org/google-docs/?Tq4xaF)

[273. Chapin, F. S. *et al.* Increasing Wildfire in Alaska’s Boreal Forest: Pathways to Potential Solutions of a Wicked Problem. *BioScience* **58**, 531–540 (2008).](https://www.zotero.org/google-docs/?Tq4xaF)

[274. Vanderlinden, L. A. Applying Stand Replacement Prescribed Fire in Alaska. in *The Use of Fire in Forest Restoration: A General Session at the Annual Meeting of the Society for Ecological Restoration, September 14-16, 1995* 78–80 (Intermountain Forest and Range Experiment Station, Forest Service, U.S. Department of Agriculture, Seattle, WA, 1996).](https://www.zotero.org/google-docs/?Tq4xaF)

[275. Haggstrom, D. Alaska Wildlife Habitat Enhancement. (1999).](https://www.zotero.org/google-docs/?Tq4xaF)

[276. Little, J. M., Jandt, R. R., Drury, S., Molina, A. & Lane, B. Evaluating the effectiveness of fuel treatments in Alaska - Final Report to the Joint Fire Science Program. *JFSP Project No. 14-5-01-27* (2018).](https://www.zotero.org/google-docs/?Tq4xaF)

[277. Goodman, L. F. & Hungate, B. A. Managing forests infested by spruce beetles in south-central Alaska: Effects on nitrogen availability, understory biomass, and spruce regeneration. *Forest Ecology and Management* **227**, 267–274 (2006).](https://www.zotero.org/google-docs/?Tq4xaF)

[278. Ray, L., Kolden, C. & Chapin III, F. A Case for Developing Place-Based Fire Management Strategies from Traditional Ecological Knowledge. *Ecology and Society* **17**, (2012).](https://www.zotero.org/google-docs/?Tq4xaF)

1. Defined as the regions in Alabama, Arkansas, Delaware, Florida, Georgia, Indiana, Kentucky, Louisiana, Maryland, Mississippi, Missouri, New Jersey, North Carolina, Ohio, Oklahoma, South Carolina, Tennessee, Texas, Virginia, West Virginia falling within the Temperate Broadleaf & Mixed Forests or Temperate Conifer Forests biomes. [↑](#footnote-ref-0)
2. Defined as the regions in Texas, New Mexico, Oklahoma, Missouri, Kansas, Colorado, Nebraska, Iowa, Illinois, Indiana, Wyoming, South Dakota, North Dakota, Minnesota, Montana falling within the Temperate Grasslands, Savannas & Shrublands biome, as well as all of Michigan, Wisconsin, and Minnesota. [↑](#footnote-ref-1)
